# Maximizing Active Site Density in Tin Single‐Site Catalysts for Selective Carbon Dioxide to Formate Electroreduction

**DOI:** 10.1002/anie.7872408

**Published:** 2026-08-27

**Authors:** Katharina Potemkin, Soressa Abera Chala, Chia‐Yu Chang, Keseven Lakshmanan, Meng‐Che Tsai, Tobias Rios Studer, Elnaz Ebrahimi, Nico Achenbach, Rongji Liu, Johannes Biskupek, Ute Kaiser, Bing Joe Hwang, Carsten Streb

**Affiliations:** ^1^ Department of Chemistry Johannes Gutenberg University Mainz Mainz Germany; ^2^ Sustainable Electrochemical Energy Development (SEED) Center National Taiwan University of Science and Technology Taipei Taiwan; ^3^ Department of Greenergy National University of Tainan Tainan Taiwan; ^4^ Central Facility of Electron Microscopy For Materials Science Electron Microscopy Group of Material Science, Ulm University Ulm Germany; ^5^ National Synchrotron Radiation Research Center (NSRRC) Hsinchu Taiwan

**Keywords:** CO_2_ electroreduction, coordination environment engineering, formate electrosynthesis, high‐density tin single‐site catalysts, operando spectroscopy

## Abstract

The electrocatalytic conversion of CO_2_ into value‐added chemicals is governed by catalyst structure‐reactivity relationships that remain incompletely understood, particularly regarding how coordination environment and active‐site density influence product selectivity. Herein, we report a mechanochemically assisted synthesis of high‐density tin single‐site catalysts, in which atomically dispersed Sn centers are stabilized at loadings of approximately 7.5 wt.%, exceeding the typical ∼2 wt.% limit reported for Sn single‐site systems without detectable aggregation. By tailoring the nitrogen‐rich precursor chemistry, catalysts with distinct coordination environments and controlled site densities were obtained, providing a platform to elucidate how these two factors govern the CO_2_ reduction pathway. Operando spectroscopy combined with density functional theory reveals that the local coordination environment governs intrinsic activity and selectivity by stabilizing key intermediates along the *OCHO pathway, whereas the mechanochemical process primarily enhances the density of electrochemically accessible Sn sites. The optimized catalyst achieves formate Faradaic efficiencies above 90% over a wide current‐density range of 50–500 mA cm^−2^ in a gas‐fed flow cell, reaching 92.5% at 100 mA cm^−2^, and maintains stable operation for over 100 h at 100 mA cm^−2^. This work provides mechanistic insights and design principles for selective and high‐rate CO_2_ electroreduction based on single‐site catalysts.

## Graphical abstract

High‐loading tin single‐site catalysts (>7.5 wt.% Sn) are synthesized using a nitrogen‐enriched carbon scaffold and mechanochemically assisted pyrolysis to stabilize atomically dispersed Sn. The resulting Sn/N‐C‐M catalyst enables highly selective CO_2_‐to‐formate conversion (>90% FE across 50–500 mA cm^−2^) with long‐term durability and *operando*‐verified structural stability.

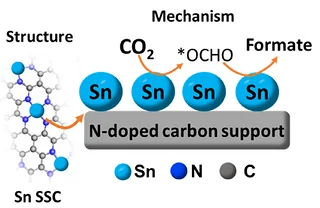

## 1 Introduction

The worldwide use of fossil fuels has led to an accumulation of greenhouse gases, particularly CO_2_, which are the primary drivers of climate change and global warming [1, 2]. Developing environmentally friendly and efficient technologies to reduce CO_2_ emissions, mitigate fossil resource depletion, and transition toward renewable energy is therefore an urgent global priority [3, 4]. The electrochemical CO_2_ reduction reaction (CO_2_RR) powered by green electricity offers a sustainable route to convert CO_2_ into carbon‐based fuels and chemical feedstocks. Recent advances in CO_2_RR have led to the production of various gaseous and liquid products [5, 6]. Among these products, some of the C_1_ products such as carbon monoxide (CO) and formate (HCOO^−^) are of great interest for the chemical and pharmaceutical industries [7, 8, 9, 10]. Specifically, the carbon‐neutral production of formate by CO_2_ electroreduction is a key process, as formate/formic acid is often used as an energy carrier in fuel cells and as a liquid hydrogen storage material [11, 12].

To date, various electrocatalysts have been developed for the electroreduction of CO_2_‐to‐formate, including catalysts based on transition metals and main‐group metals (e.g., Sn, Bi, Cu, Pb, In), as well as precious metals such as Pd [13, 14, 15, 16]. Sn‐based catalysts are especially promising due to their unique electronic structure, yet they often suffer from insufficient activity and selectivity, particularly at high‐rate catalysis [17, 18]. Previous strategies to enhance Sn catalysts have focused on doping nanostructured Sn metals, oxides, or sulfides with heteroatoms to tune active‐site electronic density, modulate intermediate binding energies, and improve CO_2_RR performance [18, 19, 20, 21]. However, a critical bottleneck is the complex Sn coordination environment, the limited number of accessible active surface sites, and the complex structural features of (often amorphous) bulk catalysts. These hurdles make it difficult to build an atomic‐level understanding of structure‐function relationships, so that knowledge‐based materials development remains a major challenge.

The electrocatalytic activity and selectivity are largely dependent on the surface structure of the catalyst, including morphology [22], particle size [23], nature/number of active sites, as well as the reaction conditions, e.g., type and pH value of the electrolyte [18, 24]. The electronic properties of the local coordination environment of the support materials also influence the catalytic activity and selectivity of Sn‐based catalysts [24]. Nitrogen‐doped carbons are particularly appealing supports, as N doping enhances electronic conductivity and adsorption properties [25]. In addition, pyridinic‐N atoms can act as an active site for CO_2_‐to‐CO conversion [26], while the incorporation of HER‐active metal species such as Ni [27] or Fe [28] transforms pyridinic‐N into highly active M‐N moieties for CO_2_‐to‐CO conversion. Despite these advances, the interaction between N‐doped carbon matrices and p‐block metals such as Sn, particularly in the context of CO_2_‐to‐formate electrocatalysis, remains underexplored. Thus, atomic‐level understanding of how coordination environment and active‐site density govern reaction pathways remains lacking.

Single‐site catalysts (SSCs), with atomically dispersed metal sites in well‐defined coordination environments, provide a unique platform to establish direct structure‐function relationships in heterogeneous catalysis. A long‐standing challenge in single‐site catalysis is the simultaneous achievement of high active‐site densities and atomic dispersion, as aggregation at elevated loadings limits both practical catalytic performance and systematic investigation of structure‐function relationships. While Fe‐, Co‐, and Ni‐based SSCs have been extensively studied for CO_2_‐to‐CO conversion [27, 28, 29, 30, 31], Sn‐based SSCs are particularly attractive for selective CO_2_‐to‐formate electroreduction. However, their development is hindered by two interconnected limitations: (i) typically low achievable Sn loadings due to aggregation during high‐temperature synthesis, and (ii) incomplete understanding of how coordination environment and site density influence formate‐selective pathways. As a result, most reported Sn single‐site systems are limited to sub‐2 wt.% loadings [30, 32, 33], restricting both mechanistic studies and practical catalytic performance.

Herein, we address these challenges by constructing Sn single‐site catalysts with high densities of electrochemically accessible active sites and tunable local coordination environments through a mechanochemically assisted, nitrogen‐rich precursor engineering strategy. This approach enables stabilization of atomically dispersed Sn species at loadings exceeding 7.5 wt.% while preserving structural integrity. The resulting model catalyst series provides a platform to disentangle how local coordination environment and electrochemically accessible site density jointly govern the CO_2_ reduction pathway. Combined experimental and theoretical investigations reveal that the local Sn coordination motif regulates the stabilization of *OCHO intermediate and thus the intrinsic activity and selectivity toward formate formation, whereas the enhanced overall catalytic performance primarily originates from the increased density of electrochemically accessible Sn sites. These findings establish a direct structure‐selectivity relationship for Sn‐based CO_2_RR catalysts and provide design principles for high‐rate, selective CO_2_ electroreduction.

## 2 Results

### 2.1 Synthesis and Characterization

Figure 1 shows the synthesis of Sn‐based SSCs via a mechanochemically assisted pyrolysis route. Initially, a nitrogen‐doped carbon support was prepared by the pyrolysis of ZIF‐8 under an argon atmosphere at 950°C for 3 h, followed by acid leaching to remove residual Zn^2+^. Subsequently, Sn incorporation was achieved by refluxing N‐C with tin(II) chloride in methanol under nitrogen gas‐protected conditions, yielding an intermediate (**i‐Sn/N‐C**), in which Sn species are coordinated within the nitrogen‐containing carbon matrix. The main challenge in synthesizing SSCs via pyrolysis of ZIF‐8 is the limited nitrogen functionality of the carbon matrix, which can hinder the stabilization of isolated metal sites and promote metal aggregation. In our design, nitrogen‐rich precursors, including melamine, dicyandiamide, and thiourea, were introduced to increase nitrogen functionalities and tailor the coordination environment of Sn species during thermal treatment. The resulting **i‐Sn/N‐C** intermediate was mechanochemically mixed with melamine/dicyandiamide/thiourea via ball milling, followed by activation by pyrolysis under argon at 800°C for 3 h to obtain **Sn/N‐C‐M**, **Sn/N‐C‐D**, and **Sn/N‐C‐T** (where M, D, and T denote melamine, dicyandiamide, and thiourea, respectively). A reference sample without additional nitrogen precursor (**Sn/N‐C**) was also prepared for comparison. The incorporation of nitrogen‐rich precursors modifies the density and configuration of nitrogen functionalities in the carbon matrix, which can serve as anchoring sites for Sn species during thermal treatment.

**FIGURE 1:**
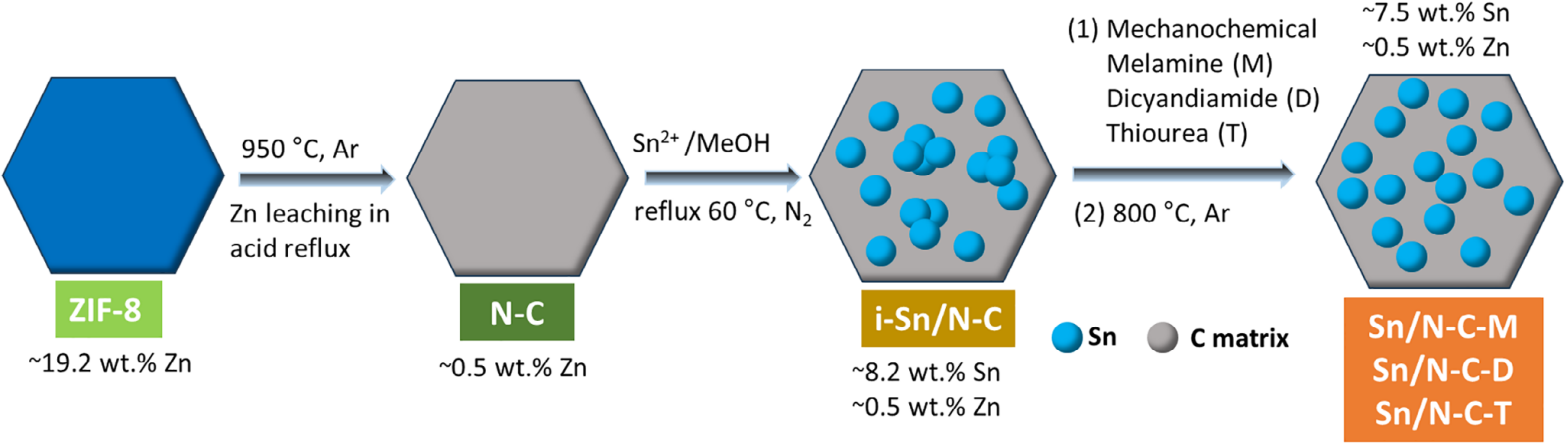
Synthesis route to atomically dispersed Sn‐N_x_ single‐site catalysts. ZIF‐8 was pyrolyzed at 950°C to form an N‐doped carbon matrix containing Zn‐N_x_ sites, followed by Zn^2+^ leaching and Sn^2+^ coordination to obtain the intermediate **i‐Sn/N‐C**. Subsequent mechanochemically assisted pyrolysis at 800°C with nitrogen‐rich precursors activated the catalyst, yielding atomically dispersed Sn‐N_x_ sites embedded within the N‐C framework.

The detailed morphology and elemental composition of the catalysts and **N‐C** support were analyzed using SEM (Figures 2a and SI, Figures S1,S5). All catalysts inherited the rhombic dodecahedral shape of the ZIF‐8‐derived **N‐C** precursors, except that the shape of some particles became uneven and showed some shrinkage, probably due to the mechanochemical/pyrolysis treatment. The TEM and high‐resolution TEM images (Figures 2b,c and S6) reveal predominantly amorphous structure of **Sn/N‐C‐M** without discernible crystalline domains. Consistently, selected area electron diffraction (SAED) patterns (Figure 2d) show diffuse rings characteristic of low‐crystallinity carbon materials. The high‐angle annular dark‐field scanning TEM (HAADF‐STEM) images of **Sn/N‐C‐M** (Figure 2e) and the corresponding energy‐dispersive X‐ray spectroscopy (EDS) mapping confirm uniform spatial distribution of Sn, N, and C throughout the polyhedral particles. Related TEM/HAADF‐STEM images of the Sn/N‐C‐D, Sn/N‐C‐T, and Sn/N‐C are shown in the SI, Figures S7–S12. Note that the introduction of nitrogen‐rich precursors and the mechanochemical process had little effect on particle morphology, while promoting an increase in the nitrogen content of the carbon matrix, which facilitated the exposure of a high density of atomically distributed reactive Sn sites and electronic conductivity. Powder X‐ray diffraction patterns (Figure 2f) show two broad diffraction peaks at ∼24° and 44°, corresponding to the (002) and (100) planes of the low crystalline graphitic domains [34, 35]. No diffraction peaks associated with metallic Sn or crystalline Sn phases are detected in any of the samples, indicating the absence of long‐range ordered Sn phases. Raman spectra (Figure S13) display characteristic D and G bands, associated with disordered and graphitic carbon, respectively. The **Sn/N‐C‐M** sample exhibits a relatively higher I_D_/I_G_ ratio (see SI, Figure S13), suggesting a more defect‐rich carbon structure, which is consistent with increased heteroatom incorporation. This result indicates the presence of an increased number of defect sites in **Sn/N‐C‐M** [35], which can act as anchoring sites for a stable binding of atomically isolated Sn sites.

**FIGURE 2:**
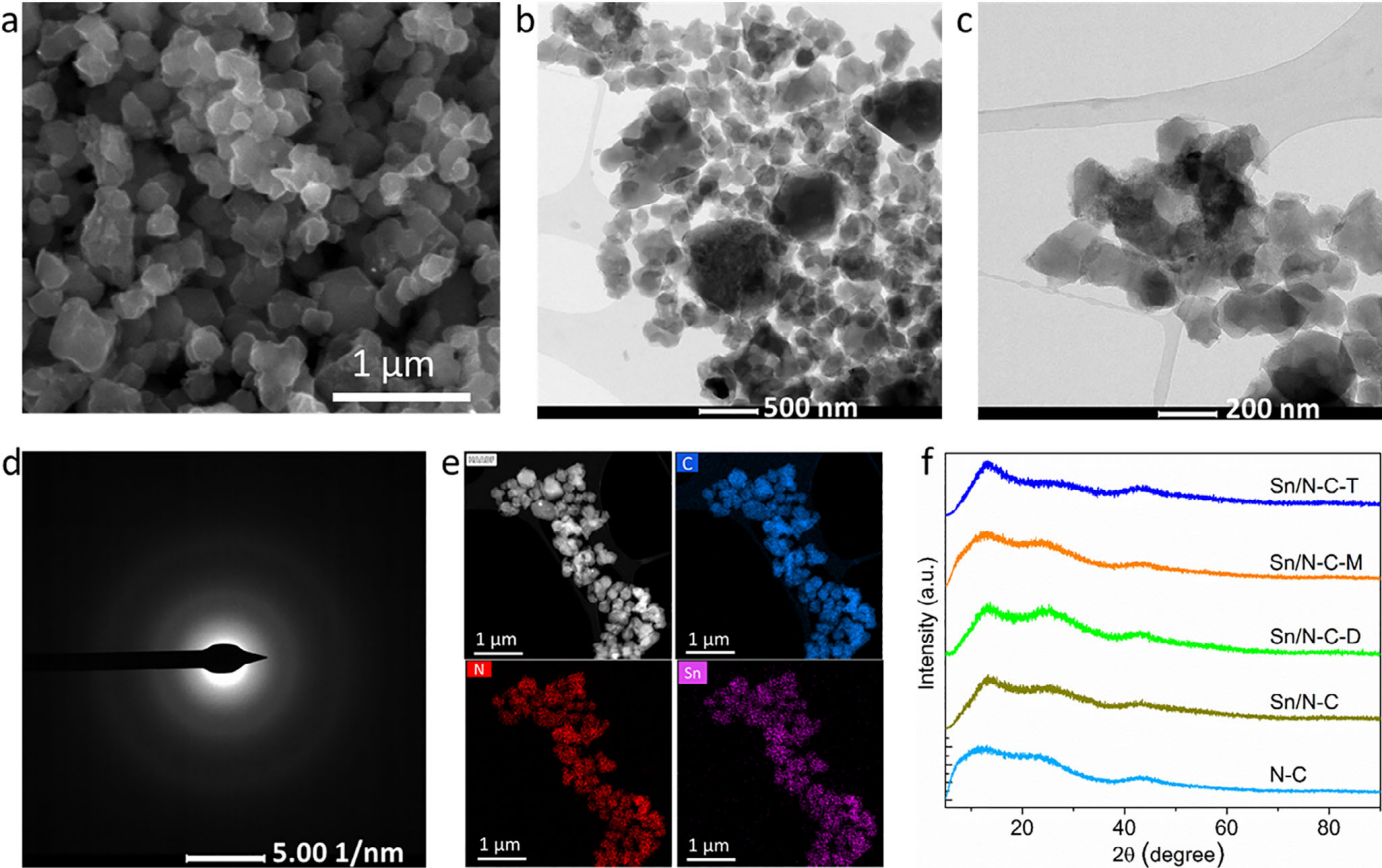
(a) SEM image, (b, c) TEM images at different magnifications, (d) selected area diffraction (SAED) image acquired from the ensemble shown in (c), (e) HAADF‐STEM image and the corresponding EDS elemental mappings of **Sn/N‐C‐M**. (f) XRD patterns of the as‐prepared Sn SSCs samples and **N‐C** support.

X‐ray photoelectron spectroscopy (XPS) was performed to investigate the elemental composition of the surface and the oxidation state of the Sn sites of the catalysts. The Sn 3d XPS spectra of the **Sn/N‐C‐M**, **Sn/N‐C‐D**, **Sn/N‐C‐T** and **Sn/N‐C** catalysts (Figure S14a) were deconvoluted into two peaks at ca. 495.4 and 487.0 eV, assigned to Sn 3d_3/2_ and Sn 3d_5/2_, respectively [18, 32]. This result confirms that Sn is present in the +2 oxidation state in all samples.

The S 2p spectra (Figure S14b) demonstrate the successful incorporation of sulfur into the **Sn/N‐C‐T** sample. The N 1s spectra (Figure S15) reveal contributions from pyridinic (398.4 eV), pyrrolic (400.4 eV), graphitic (401.6 eV), and oxidized (403.2 eV) nitrogen species [36, 37, 38]. An additional feature centered at approximately 398.6 eV is also observed. However, owing to the small binding‐energy difference relative to pyridinic nitrogen and the intrinsic peak broadening of N‐doped carbon materials, this feature cannot be unambiguously assigned solely based on N 1s XPS. It is therefore described as being consistent with Sn‐associated nitrogen functionalities rather than as definitive evidence for a specific Sn‐N coordination motif [39, 40]. The relative proportion of pyridinic nitrogen increases in the order **Sn/N‐C** < **Sn/N‐C‐T** < **Sn/N‐C‐D** < **Sn/N‐C‐M** (Table S1), reflecting the influence of nitrogen‐rich precursors. It should be noted that the pyridinic/pyrrolic ratios obtained from XPS represent the overall nitrogen speciation within the carbon matrix and do not directly identify the local coordination environment of the atomically dispersed Sn centers.

Such nitrogen functionalities may nevertheless influence the formation and stabilization of Sn‐N_x_ moieties within the carbon framework [41]. Inductively coupled plasma optical emission spectroscopy confirms relatively high Sn loadings of 7.5–8.2 wt.% across the catalyst series (Table S2), exceeding typical values reported for Sn‐based SSCs [30, 32, 33, 42]. It is worth noting that the atomically dispersed Sn content in most Sn‐based SSCs reported in the literature is typically 2 wt.% or less, and remains low due to the limited density of nitrogen species/metal anchoring sites in the support materials. These results demonstrate that the Sn incorporation strategy employed here enables the stabilization of high concentrations of Sn species within the carbon matrix. The introduction of additional nitrogen species through the mechanochemical pyrolysis approach further modifies the local coordination environment of Sn species by providing additional nitrogen functionalities, thereby promoting the formation and stabilization of atomically dispersed Sn sites. Thus, the mechanochemical pyrolysis strategy plays a crucial role in regulating the coordination environment and dispersion of Sn single‐sites rather than increasing the overall Sn loading.

In addition, HAADF‐STEM images (Figures 3a,b and S16–S18) reveal a high density of discrete bright spots uniformly distributed across the carbon matrix in all samples, consistent with atomically dispersed Sn rather than clusters or nanoparticles.

**FIGURE 3:**
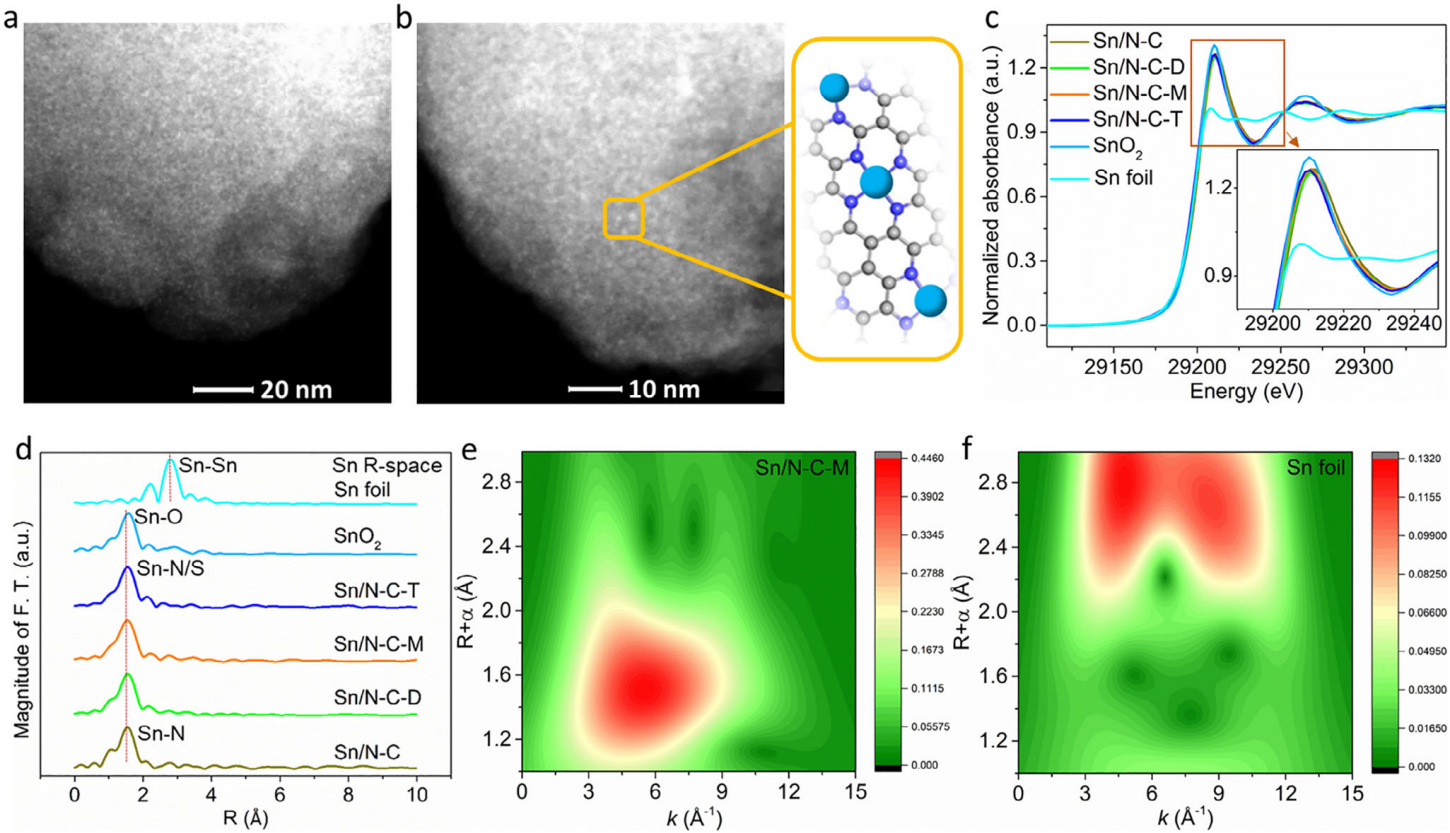
(a, b) Aberration‐corrected HAADF‐STEM images. (c) Normalized Sn K‐edge XANES spectra of **Sn/N‐C**, **Sn/N‐C‐M**, **Sn/N‐C‐D**, **Sn/N‐C‐T** catalysts, and Sn foil and SnO_2_ as reference samples. (d) Magnitude of the Fourier transforms of the EXAFS spectra of **Sn/N‐C**, **Sn/N‐C‐M**, **Sn/N‐C‐D**, **Sn/N‐C‐T** catalysts, and reference samples. (e, f) Wavelet transform EXAFS of the *k* ^2^‐weighted *k‐*space for **Sn/N‐C‐M** catalyst and Sn foil.

To further probe the local coordination environments of Sn, synchrotron‐based X‐ray absorption spectroscopy (XAS) was performed. The Sn K‐edge X‐ray absorption near edge structure (XANES) spectra (Figure 3c) indicate an average oxidation state between metallic Sn and SnO_2_, consistent with Sn^2+^ species, in agreement with XPS analysis. Fourier‐transformed extended X‐ray absorption fine structure (EXAFS) spectra (Figure 3d) show a dominant peak at 1.53 Å, corresponding to Sn coordination with light atoms (N, C, O, or S) [30, 43], and no detectable Sn‐Sn scattering at longer radial distances, indicating the absence of metallic clustering, which is consistent with the HAADF‐STEM results. The corresponding oscillation curves in the Sn K‐space (Figure S19) exhibit lower amplitudes compared to SnO_2_, but higher amplitudes compared to Sn foil. This difference likely arises from variations in the coordination environment of the Sn atoms and electronic effects induced by the surrounding atoms. Also, the wavelet transform (WT) analysis of the Sn K‐edge EXAFS oscillations was performed to further demonstrate the atomic dispersion of Sn species, which has the property of strong resolution in both *k‐* and *R‐*spaces [35]. The WT analysis (Figures 3e and S20) supports this observation, showing only a low‐k intensity maximum associated with Sn‐light atom coordination, whereas the high‐k feature associated with Sn‐Sn scattering observed for metallic Sn is absent [44, 45]. Quantitative EXAFS fitting analysis (Figures S21 and Table S3) was performed using plausible first‐shell coordination models. Among the models considered, the Sn‐N coordination model provides the best agreement with the experimental spectra, yielding coordination numbers close to four for **Sn/N‐C‐M**, **Sn/N‐C‐D**, and **Sn/N‐C**, whereas **Sn/N‐C‐T** is best described by a mixed S‐Sn‐N_3_ coordination environment. We note that EXAFS alone cannot unambiguously distinguish N from other light scatterers such as C and O because of their similar backscattering amplitudes. Therefore, the assignment of predominantly Sn‐N coordination is based on the superior fitting quality of the Sn‐N model together with complementary evidence, including the nitrogen‐rich precursor chemistry, the abundant nitrogen functionalities revealed by XPS, the atomic dispersion observed by HAADF‐STEM, the WT‐EXAFS analysis showing no Sn‐Sn contribution, and the absence of evidence for crystalline Sn phases.

### 2.2 Electrocatalytic CO_2_RR Performance

The electrocatalytic CO_2_RR performances of **Sn/N‐C‐M**, **Sn/N‐C‐D**, **Sn/N‐C‐T** and **Sn/N‐C** catalysts were initially evaluated by cyclic voltammetry (CV) and linear sweep voltammetry (LSV) under Ar‐ and CO_2_‐saturated 0.1 M aqueous KHCO_3_ (pH = 6.9) electrolyte in an H‐type cell separated by a Nafion 117 membrane. As shown in SI, Figures S22–S25, all samples under CO_2_‐saturated electrolyte exhibited a higher reduction current density and a shift in onset potential compared to Ar‐saturated electrolyte, implying the occurrence of CO_2_ reduction.

Among the catalyst series, **Sn/N‐C‐M** catalyst displays the highest current density and most favorable onset potential (Figure 4a), reflecting superior overall electrocatalytic performance toward CO_2_ reduction. This enhanced performance is associated with differences in the nitrogen‐rich carbon matrix and the resulting local coordination environment and accessibility of the atomically dispersed Sn sites. These structural variations are expected to modify the electronic structure of the Sn centers and thereby influence the adsorption and conversion of CO_2_ reaction intermediates. Quantitative product analysis was performed by controlled reductive potential electrolysis followed by ^1^H‐NMR (for liquid products) and GC (for gaseous products). Representative ^1^H‐NMR spectra of the CO_2_RR products as well as calibration curves and GC chromatograms over each catalyst are shown in Figures S26–S31. These data were used to calculate the FEs for each product produced at each reductive potential. Figures 4b and S32–S35 summarize the product distribution and potential‐dependent CO_2_RR FE for **Sn/N‐C‐M**, **Sn/N‐C‐D**, **Sn/N‐C‐T** and **Sn/N‐C** catalysts. The main CO_2_RR product on each catalyst is formate, together with a small amount of CO, H_2_, CH_4_, and C_2_/C_2+_ products such as C_2_H_4_, and EtOH. The **Sn/N‐C‐M** catalyst achieves a maximum formate Faradaic efficiency (FE) of 82.5% at −1.3 V vs. RHE, outperforming **Sn/N‐C‐D** (72.5%), **Sn/N‐C‐T** (68.6%), and **Sn/N‐C** (68.1%) (Figure 4b,c). This trend is consistent across the investigated potential range, indicating that the observed differences in catalytic performance are intrinsic to the catalyst structures rather than experimental artifacts. The superior performance of **Sn/N‐C‐M** is further reflected in its higher formate partial current density (−24.5 mA cm^−2^ at −1.3 V vs. RHE, Figure 4d). The origin of this enhanced overall activity is examined in the following section through analyses of the apparent turnover frequency and electrochemical surface area. Catalysts prepared using different nitrogen‐rich precursors exhibit lower formate activity and selectivity, highlighting the importance of precursor‐controlled catalyst structure. Notably, the formation of C_2_/C_2+_ products is significantly suppressed on **Sn/N‐C‐M** (3.0% FE) compared to **Sn/N‐C** (20.2% at −0.9 V vs. RHE, Figure 4e). This behavior may arise from differences in the contribution of defect‐associated sites within the nitrogen‐doped carbon matrix. In **Sn/N‐C**, defect‐rich carbon domains may influence reaction pathway and contribute to the formation of minor C_2_/C_2+_ products, consistent with previous reports showing that N‐doped carbon domains in hybrid electrocatalysts can modulate CO_2_RR intermediates and product selectivity [46]. In contrast, the higher density of atomically dispersed Sn species in **Sn/N‐C‐M** may more effectively suppress carbon‐mediated C_2_/C_2+_ formation, thereby promoting selective formate production.

**FIGURE 4:**
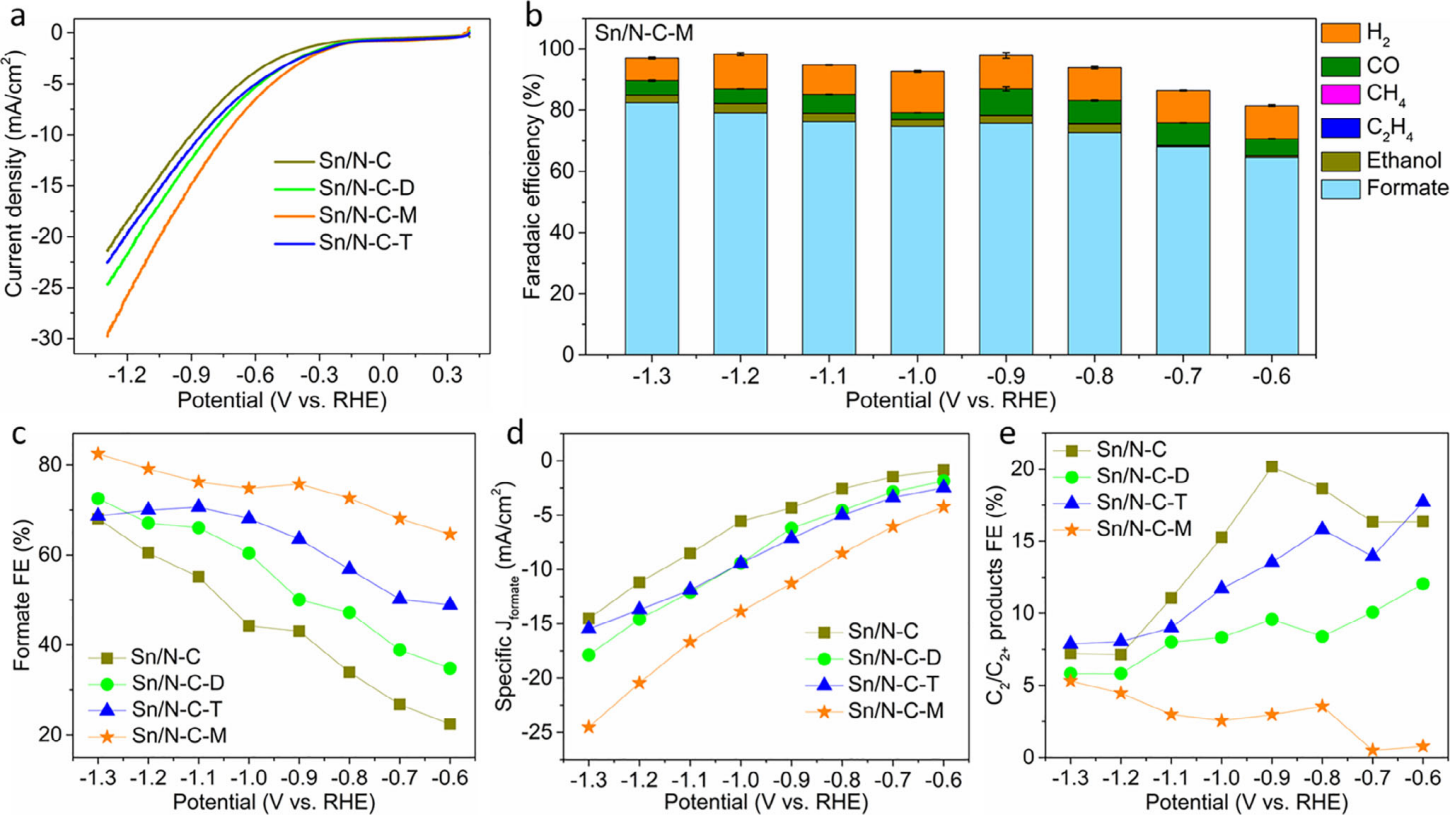
(a) LSV curves obtained for different Sn‐SSCs in CO_2_‐saturated 0.1 M aqueous KHCO_3_ (pH = 6.9) electrolyte. (b) FEs and product distribution of the **Sn/N‐C‐M** catalyst at different controlled potentials in a typical H‐type electrolytic cell. (c) FE of electroreduction of CO_2_‐to‐formate over different Sn‐SSCs at different controlled potentials. (d) Formate partial current density; (e) FE of C_2_/C_2+_ formation for different Sn‐SSCs at different controlled potentials.

The catalytic activity and product distribution of the nitrogen‐doped carbon matrix, derived from ZIF‐8 via pyrolysis followed by Zn removal, was evaluated as a control (see Figures S36–S38). In contrast to Sn‐based catalysts, **N‐C** predominantly produces H_2_ and CO, with CO as the major product, and no detectable formate. This confirms that atomically dispersed Sn sites are essential for selective CO_2_‐to‐formate conversion. Furthermore, the absence of formate formation on **N‐C** indicates that residual Zn species do not contribute measurably to the observed catalytic activity. The apparent turnover frequency (TOF) of formate formation was estimated based on the measured FE, current density, and total Sn content determined by ICP‐OES (Figure 5a). Because this calculation assumes that all Sn atoms are electrochemically accessible, the resulting values represent apparent TOFs, providing a lower limit for the intrinsic turnover frequency. At −1.3 V vs. RHE, **Sn/N‐C‐M** exhibits the highest apparent TOF (1450 h^−1^) followed by **Sn/N‐C‐D** (1057 h^−1^), **Sn/N‐C‐T** (915 h^−1^), and **Sn/N‐C** (860 h^−1^), demonstrating its superior overall catalytic performance. To assess the contribution of electrochemically accessible active‐site density, the electrochemical surface areas (ECSA) was estimated from the double‐layer capacitance (C_dl_) (see Figures S39 and S40a). Although C_dl_‐derived ECSA does not directly quantify the number of active Sn sites, it serves as a useful proxy for the electrochemically accessible catalytic surface. **Sn/N‐C‐M** exhibits a higher C_dl_‐derived ECSA (1367 cm^2^) compared to **Sn/N‐C‐D** (881 cm^2^), **Sn/N‐C‐T** (681 cm^2^), and **Sn/N‐C** (181 cm^2^), indicating a greater electrochemically accessible surface. To further distinguish the contributions of accessible site density and intrinsic catalytic activity, the apparent TOF, the total current density, and formate partial current density were normalized by the ECSA (Figure S40b–e). After normalization, **Sn/N‐C** exhibits the highest apparent activity per electrochemically accessible surface area, whereas **Sn/N‐C‐M** shows a lower ECSA‐normalized activity despite its superior overall performance. These results indicate that the enhanced catalytic performance of **Sn/N‐C‐M** primarily originates from its substantially higher density of electrochemically accessible active sites, which compensates for its lower apparent intrinsic activity per accessible surface area. Isotopic labeling experiments using ^13^CO_2_ were conducted to confirm the origin of formate product. In ^1^H‐NMR spectra, electrolysis under ^13^CO_2_ produces a characteristic doublet corresponding to ^13^C‐coupled formate (Figure 5b), whereas only a singlet is observed under ^12^CO_2_. In addition, no products were detected in the electrolysis carried out under Ar‐saturated electrolyte (Figure S41). These results unambiguously confirm that formate is derived from CO_2_ reduction rather than from catalyst decomposition or residual carbon species. Time‐dependent electrolysis further shows a progressive increase in the formate signal intensity (Figure 5c), while maintaining a Faradaic efficiency above 80% over extended operation (Figure 5d), indicating stable catalytic performance. Continuous electrolysis at −1.3 V vs. RHE for 27 h reveals minimal changes in current density and product selectivity (Figure 5e), demonstrating good operational stability. Post‐reaction characterization using SEM, XRD, and XPS (Figures S42–S44) shows no evidence of nanoparticle formation, structural degradation, or significant changes in Sn oxidation state. These observations support the structural robustness of the atomically dispersed Sn sites under CO_2_RR conditions.

**FIGURE 5:**
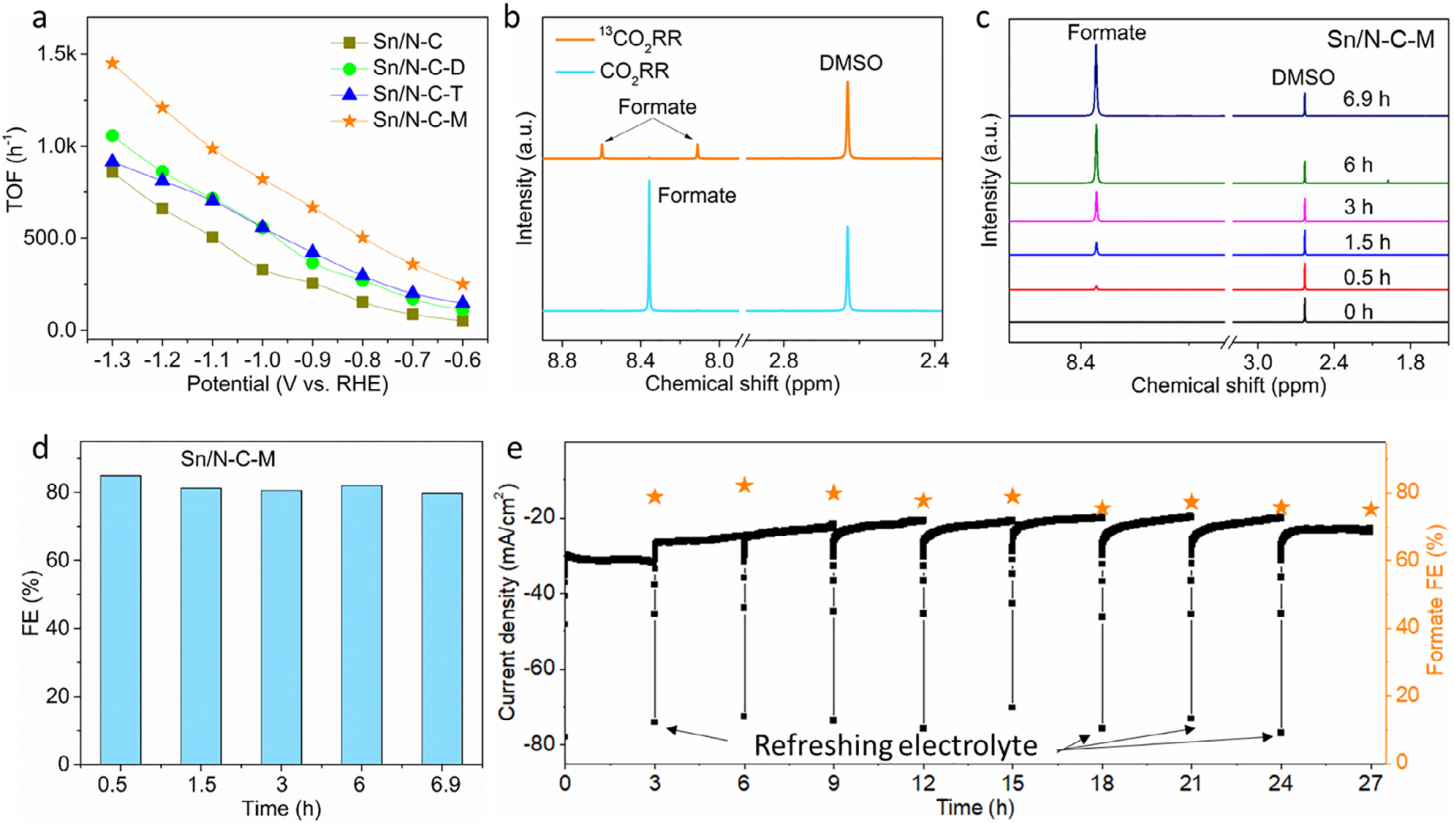
(a) Apparent TOFs for formate formation for different Sn‐SSCs at different controlled potentials. (b) ^1^H‐NMR spectra of isotopically labeled ^13^CO_2_RR and CO_2_RR using **Sn/N‐C‐M** at −1.3 V vs. RHE. Formate signals are weaker in the ^13^CO_2_ spectrum due to the lower concentration of dissolved ^13^CO_2_, as the electrolyte was bubbled with ^13^CO_2_ for 20 min compared to 1 h for CO_2_. (c) ^1^H‐NMR spectra of the electrolyte after CO_2_RR at different sampling times for **Sn/N‐C‐M**, (d) the corresponding FE of formate. (e) The stability test by chronoamperometric measurement (black curve) and the corresponding FE of formate (orange star) using **Sn/N‐C‐M** catalyst at −1.3 V versus RHE in a typical H‐type electrolytic cell.

The electrocatalytic performance of **Sn/N‐C‐M** catalyst was further evaluated in a gas‐fed flow electrolyzer using a gas diffusion electrode (GDE) configuration (Figure 6a). The introduction of the GDE enables the formation of three interphases (gas‐liquid‐solid), facilitating efficient CO_2_ mass transport to the catalyst surface and allowing operation at elevated current densities compared to conventional H‐type cells. As shown in Figure 6b, the LSV curves recorded in the flow cell exhibit current densities exceeding 400 mA cm^−2^ at −1.2 V versus RHE. Controlled‐current electrolysis reveals that **Sn/N‐C‐M** maintains a high Faradaic efficiency for formate (>90%) over a wide current density range (50‐500 mA cm^−2^), reaching a maximum of 92.5% at 100 mA cm^−2^ (Figure 6c). This performance compares favorably with the recently reported Sn‐based catalysts (SI, Table S7) [15, 24, 32, 47, 48, 49, 50, 51, 52, 53, 54, 55, 56, 57, 58, 59]. The corresponding formate partial current density increases with applied current density, reaching 451 mA cm^−2^ at 500 mA cm^−2^. The apparent TOF, estimated from the measured current, formate Faradaic efficiency, and total Sn loading, also increases under these conditions, although these values should be regarded as apparent because they assume that all Sn atoms are electrochemically accessible. Consistent with electrochemical measurements, the ^1^H‐NMR spectra (Figure 6d) show progressively increasing formate signal intensities with increasing current density, confirming efficient formate production under high‐rate operating conditions. The **Sn/N‐C‐M** catalyst also demonstrates stable operation in the flow cell, maintaining formate Faradaic efficiencies above 85% during continuous electrolysis at 100 mA cm^−2^ for 100 h, with minimal variation in cathodic potential (Figure S45). In addition, the catalyst achieves a cathodic energy efficiency of 58.6%, while no detectable formate crossover is observed in the half‐cell configuration using Nafion 117 membrane (Table S4), highlighting efficient product utilization under practical operating conditions. A comparison with previously reported Sn‐based catalysts (Figure 6e and Table S7) [15, 24, 47, 48, 49, 50, 51, 52, 53, 54] indicates that **Sn/N‐C‐M** exhibits competitive performance in terms of formate selectivity and partial current density under gas‐fed conditions. These results highlight the effectiveness of combining atomically dispersed Sn sites with a gas‐diffusion electrode architecture to achieve efficient CO_2_ reduction at high current densities. To further evaluate the operational robustness of the **Sn/N‐C‐M** catalyst under continuous electrolysis conditions, a full‐cell flow electrolyzer equipped with a bipolar membrane (BPM) was assembled using **Sn/N‐C‐M** deposited on a GDE as the cathode and commercial IrO_2_ as the anodic oxygen evolution catalyst. During galvanostatic electrolysis at 100 mA cm^−2^, the full cell electrolyzer maintained stable operation for 100 h with formate remaining the dominant liquid product throughout the testing period (Figure S46). The corresponding full‐cell energy efficiency reached 45.8%, while the formate crossover was limited to 13.7% (Table S4). These results demonstrate efficient energy utilization, limited product crossover, and sustained high‐rate formate production under continuous full‐cell electrolysis, highlighting the practical potential of the **Sn/N‐C‐M** catalyst architecture for CO_2_ electroreduction.

**FIGURE 6:**
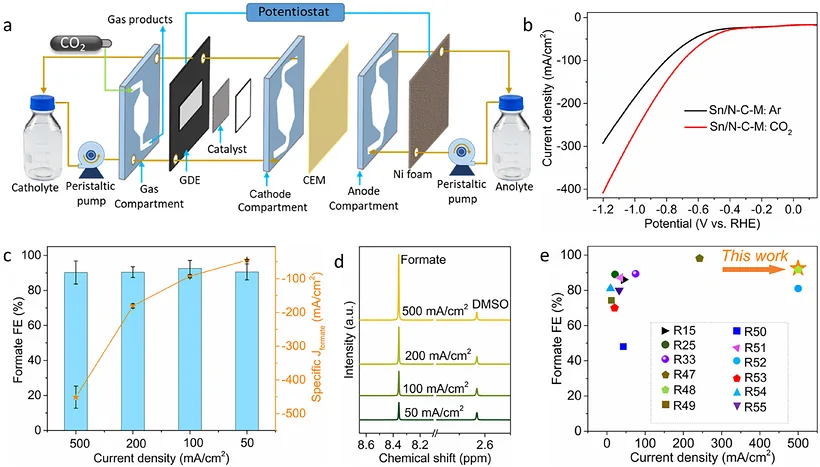
(a) Schematic diagram of a flow electrolytic cell. (b) The LSV curves of **Sn/N‐C‐M** in Ar‐ and CO_2_‐saturated 0.1 M KHCO_3_ in a flow electrolytic cell. (c) FE and partial current density of formate for **Sn/N‐C‐M** at different industrially relevant current densities. (d) ^1^H‐NMR spectra for electrolytes after CO_2_RR at different current densities. (e) Comparison of the electrocatalytic performance of Sn‐based catalysts for the electroreduction of CO_2_ to formate from recent literature. R describes the reference literature in Table S7.

### 2.3 Catalytic Mechanism Studies

To probe the structural evolution of the active sites and catalytic intermediates during the CO_2_RR, in‐situ/operando XAS measurements were conducted. Specifically, Sn K‐edge XANES and EXAFS spectra were collected in a CO_2_‐saturated 0.1 M KHCO_3_ electrolyte under various applied potentials. Operando Sn K‐edge XANES spectra (Figure 7a) show negligible edge shifts under applied potentials, indicating that Sn remains predominantly in the Sn^2+^ state during CO_2_RR. EXAFS analysis (Figure 7b) reveals no emergence of Sn‐Sn coordination, confirming the retention of atomically dispersed Sn species under reaction conditions. A slight decrease in the Sn‐N (or possibly Sn‐O/Sn‐C) scattering intensity is observed, which may be associated with dynamic interactions between adsorbates and active sites or subtle structural rearrangements, while the overall coordination environment remains largely preserved. These observations collectively support the structural robustness and single‐atom nature of the active Sn sites during CO_2_RR.

**FIGURE 7:**
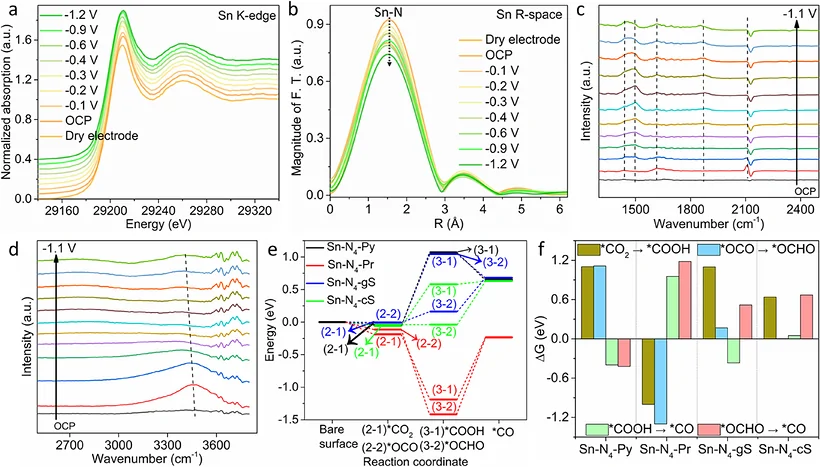
(a) Normalized *operando* Sn K‐edge XANES spectra of **Sn/N‐C‐M** recorded during CO_2_RR. The spectra are vertically offset for clarity. (b) Fourier‐transformed EXAFS spectra collected under the same conditions in a CO_2_‐saturated 0.1 M KHCO_3_ solution. (c, d) In situ ATR‐FTIR spectra of **Sn/N‐C‐M** catalyst in CO_2_‐saturated solution 0.1 M KHCO_3_ electrolyte. (e) Calculated Gibbs free‐energy diagrams for CO_2_ reduction on four proposed Sn‐N_4_ coordination environments, including pyridinic‐N‐coordinated (Sn‐N_4_‐Py) and pyrrolic‐N‐coordinated (Sn‐N_4_‐Pr) motifs representative of **Sn/N‐C‐M**, **Sn/N‐C‐D**, and **Sn/N‐C**, as well as graphitic‐S‐modified (Sn‐N_4_‐gS) and curved‐S‐modified (Sn‐N_4_‐cS) environments characteristic of **Sn/N‐C‐T**. (f) Calculated Gibbs free energies for the key intermediates in the formate and CO formation pathways on the four Sn‐N_4_ models. The formate pathway proceeds through the *OCHO intermediate, whereas the competing CO pathway proceeds through the *COOH intermediate followed by *CO formation.

To further elucidate the electroreduction of CO_2_‐to‐formate and to monitor the intermediate reactions, the synchrotron‐based electrochemical in‐situ ATR‐FTIR studies were performed for the **Sn/N‐C‐M** catalyst. A summary of the ATR‐FTIR band assignments is provided in Table S5. As shown in Figure 7c, the vibrational bands at 1436 and 1500 cm^−1^ are assigned to *OCHO and *HCOO^−^ intermediates, respectively [48, 60], consistent with the dominant formate production. The intensities of the *OCHO and *HCOO^−^ bands were quantified using integrated peak areas after linear baseline subtraction between selected anchor wavenumbers, as described in the Supporting Information. Their increasing intensity with applied potential correlates with enhanced formate formation rates. The vibration peak detected at 1615 cm^−1^ could be assigned to the adsorbed H_2_O molecule according to the previous studies [61]. The infrared band observed in the 1800–1900 cm^−1^ range especially at the potential stepping from −0.5 to −1.1 V vs. RHE, is related to the *CO species bound to the reactive metal sites [62]. The broad feature in the 3300–3600 cm^−1^ region decreases at more negative potentials (Figure 7d) [30], which may reflect changes in interfacial water structure; however, contributions from gas evolution or optical effects cannot be excluded. These results are consistent with a reaction pathway involving *OCHO intermediates on isolated Sn‐N_x_ sites, in agreement with prior studies on Sn‐based CO_2_RR catalysts [63].

The DFT calculations provide a coherent mechanistic picture of how variations in local coordination environment of Sn centers influence the intrinsic reaction energetics of CO_2_ reduction pathways. The structural models representing pyridinic‐N (Sn‐N_4_‐Py), pyrrolic‐N (Sn‐N_4_‐Pr), and S‐modified Sn‐N_4_ motifs were constructed to reflect the coordination environments inferred from spectroscopic characterization of the catalyst series (see Supporting Information of the computational details and Figure S47). The calculated adsorption geometries indicate that CO_2_ binding is sensitive to the local electronic structure of the Sn center. Among the investigated models, the pyrrolic‐N‐coordinated Sn‐N_4_ sites exhibit stronger stabilization of *CO_2_ intermediates, suggesting more effective activation of CO_2_. In contrast, S‐modified Sn‐N_4_ environments moderate the adsorption strength, which may help avoid overly strong binding that could hinder further subsequent proton‐coupled electron transfer steps (Figure 7e). Free‐energy profiles reveal that the *OCHO pathway is thermodynamically more favorable than the competing *COOH pathway across the investigated coordination environments, except for the Sn‐N_4_‐Py model (Figure 7f and Table S6), consistent with the experimentally observed preference for formate formation. Furthermore, the free energy for *OCHO formation depends strongly on the local coordination environment. The Sn‐N_4_‐Pr model exhibits the most favorable *OCHO formation energetics, whereas the Sn‐N_4_‐Py model displays the least favorable reaction pathway. These results indicate that the local coordination environment governs the intrinsic activity of individual Sn sites by modulating the stabilization of key reaction intermediates. Importantly, the DFT calculations describe the intrinsic reactivity of idealized Sn coordination motifs rather than the overall catalytic performance of the experimental catalysts. In contrast, the electrochemical measurements demonstrate that the measured catalytic performance is determined by two independent contributions: the intrinsic activity of the accessible Sn sites and the density of electrochemically accessible active sites. Consistent with the DFT results, the ECSA‐normalized activity follows the trend expected from the calculated *OCHO energetics, whereas the superior overall performance of **Sn/N‐C‐M** originates primarily from its substantially higher density of electrochemically accessible active sites. Thus, the experimental and theoretical results are complementary: the local coordination environment determines the intrinsic reaction energetics, while the mechanochemically assisted synthesis strategy enhances the overall catalytic performance by increasing the density of electrochemically accessible Sn sites. Although the present calculations capture key trends in intermediate stabilization and reaction energetics, it should be noted that the models do not explicitly account for solvent effects, dynamic restructuring, or mass transport limitations present under operating conditions. Nevertheless, the qualitative agreement between calculated intrinsic activity trends and the experimental observations provides mechanistic insight into the respective roles of local coordination environment and electrochemically accessible active‐site density in governing CO_2_ electroreduction on Sn single‐site catalysts.

### 2.4 Mechanistic Discussion

The **Sn/N‐C‐M** catalyst exhibits significantly enhanced activity and selectivity for formate formation compared to **Sn/N‐C‐D**, **Sn/N‐C‐T**, and **Sn/N‐C**, despite comparable Sn loadings (7.5–8.2 wt.%, see Table S2). This observation indicates that catalytic performance is governed not solely by the total metal loading, but instead reflects the combined influence of the local coordination environment and the density of electrochemically accessible active sites. Sn K‐edge EXAFS and XANES analyses confirm the predominance of atomically dispersed Sn‐Nx sites across all samples, while variations in precursor chemistry lead to distinct nitrogen functionalities of the carbon matrix and local coordination motifs. The higher activity of **Sn/N‐C‐M** is accompanied by a substantially larger C_dl_‐derived electrochemically accessible surface area than the reference catalysts (Figure S40a). Although C_dl_ does not directly quantify the number of active sites, the increased ECSA is consistent with a higher density of electrochemically accessible catalytic sites and more effective utilization of the atomically dispersed Sn species. In addition, the nitrogen‐rich carbon matrix may facilitate charge transport and contribute to enhanced reaction kinetics. DFT calculations further demonstrate that variations in the local coordination environment modify the electronic structure of the Sn centers and thereby influence CO_2_ adsorption and the energetics of key reaction intermediates. Thus, the *OCHO pathway is thermodynamically preferred over the competing *COOH pathway, consistent with the experimentally observed formate selectivity. However, the calculated *OCHO formation energies vary among the different coordination motifs, indicating that the local coordination environment governs the intrinsic activity of individual Sn sites. These theoretical results are consistent with the experimentally observed differences in ECSA‐normalized activity, whereas the superior overall catalytic performance of **Sn/N‐C‐M** originates primarily from its markedly higher density of electrochemically accessible active sites. These findings agree with *operando* ATR‐FTIR measurements, which show increasing *OCHO‐related signals under reaction conditions, correlating with formate formation. *Operando* XAS further confirms that the atomically dispersed Sn coordination environment remains stable throughout electrolysis without detectable Sn aggregation or change in oxidation state.

In a gas‐fed flow cell, **Sn/N‐C‐M** maintains high formate selectivity over a wide current density range (50‐500 mA cm^−2^) and exhibits stable operation over extended electrolysis at 100 mA cm^−2^. At 500 mA cm^−2^, the catalyst delivers a formate Faradaic efficiency exceeding 90% with a partial current density of 451 mA cm^−2^ and an energy efficiency of 50.2%. The corresponding apparent turnover frequency reaches 13284 h^−1^_,_ reflecting the outstanding overall catalytic performance achieved through the high density of electrochemically accessible Sn sites. Continuous full‐cell electrolysis in a BPM‐based flow electrolyzer at 100 mA cm^−2^ for 100 h further confirms the structural robustness and long‐term operational stability of the catalyst. These performance metrics place **Sn/N‐C‐M** among the highest‐performing reported Sn single‐site and nanostructured catalysts under comparable electrochemical conditions (see Figure 6e and Table S7) [15, 24, 47, 48, 49, 50, 52, 53, 54]. Collectively, these results demonstrate that high‐rate, selective CO_2_‐to‐formate electroreduction requires simultaneous optimization of the intrinsic reactivity of the Sn coordination environment and the density of electrochemically accessible active sites.

## 3 Conclusion

In summary, we have developed a series of high‐loading Sn single‐site catalysts through a mechanochemically assisted pyrolysis strategy, enabling atomically dispersed Sn centers at metal loadings exceeding 7.5 wt.% without detectable aggregation. Beyond overcoming the loading limitation typically encountered in Sn single‐site catalysts, this catalyst platform enables systematic investigation of how local coordination environment and the density of electrochemically accessible active sites jointly govern the CO_2_ reduction pathway. Combined experimental characterization, operando spectroscopy, and theoretical analyses demonstrate that the local coordination environment regulates the intrinsic reaction energetics by modulating the stabilization of the *OCHO intermediate, whereas the mechanochemical synthesis primarily enhances the density of electrochemically accessible Sn sites, resulting in superior overall catalytic performance. The optimized **Sn/N‐C‐M** catalyst delivers high formate Faradaic efficiency (>90%) over a wide current density range in a gas‐fed flow cell and exhibits sustained stability during extended electrolysis, including continuous full‐cell operation in a BPM‐based flow electrolyzer for 100 h. These findings demonstrate that high‐rate, selective CO_2_‐to‐formate electroreduction requires simultaneous optimization of the intrinsic reactivity of the local Sn coordination environment and the density of electrochemically accessible active sites. More broadly, this work establishes a mechanistic framework for disentangling the respective roles of local coordination environment and accessible active‐site density in single‐site catalysts, providing design principles for the rational development of high‐performance electrocatalysts for selective CO_2_ conversion under industrially relevant operating conditions.

## Author Contributions

S.A.C. and C.S. conceptualized and designed the study. S.A.C. and K.P. carried out the syntheses and characterization. General characterization and electrochemical analyses were performed by S.A.C., K.P., T.R.S., and R.L. In situ ATR‐FTIR and Raman experiments were conducted by K.L. GC and NMR analyses were carried out by S.A.C. and K.P., while N.A. performed elemental analyses. Electron microscopy and associated data interpretation were carried out by E.E., J.B., and U.K. Computational studies were performed by M.C.T. K.L. and C.Y.C. conducted XAS experiments and fitted the data. B.J.H. provided support for synchrotron‐based XAS, in situ ATR‐FTIR experiments, and data validation. S.A.C and C.S. supervised the project, while C.S. acquired funding. All authors contributed to writing the manuscript.

## Conflicts of Interest

The authors declare no conflict of interest.

## Supporting information




**Supporting File**: anie74433‐sup‐0001‐S2.pdf.

## Data Availability

The data that support the findings of this study are openly available in Zenodo at https://zenodo.org, reference number 21517486.
