# Supplementary material for: Maximizing Active Site Density in Tin Single‐Site Catalysts for Selective Carbon Dioxide to Formate Electroreduction
Source: Angew Chem Int Ed Engl. 2026 Aug 27;65(43):e7872408. doi: 10.1002/anie.7872408 (PMC13676412; doi:10.1002/anie.7872408)
Supplement: Supplementary file 1 [file ANIE-65-e7872408-s001.pdf]

Supporting Information
For
Maximizing Active Site Density in Tin Single-Site Catalysts for Selective Carbon Dioxide to Formate Electroreduction

Katharina Potemkin,[a] Soressa Abera Chala,[a]* Chia-Yu Chang,[b] Keseven Lakshmanan,[b] Meng-Che Tsai,[c] Tobias Rios Studer,[a] Elnaz Ebrahimi,[a] Nico Achenbach,[a] Rongji Liu,[a] Johannes Biskupek,[d] Ute Kaiser,[d] Bing Joe Hwang,[b,e]* and Carsten Streb[a]*

[a]Department of Chemistry, Johannes Gutenberg University Mainz, Duesbergweg 10-14, 55128 Mainz, Germany.
[b]Sustainable Electrochemical Energy Development (SEED) Center, National Taiwan University of Science and Technology, Taipei, 106335 Taiwan
[c]Department of Greenergy, National University of Tainan, Tainan, 700301, Taiwan
[d]Central Facility of Electron Microscopy for Materials Science, Electron Microscopy Group of Material Science, Ulm University, Albert-Einstein-Allee 11, 89081 Ulm, Germany
[e]National Synchrotron Radiation Research Center (NSRRC), Hsin-chu 30076, Taiwan

Contact author:
Soressa Abera Chala: soressa-abera.chala@uni-mainz.de
Bing-Joe Hwang: bjh@mail.ntust.edu.tw
Carsten Streb: carsten.streb@uni-mainz.de

Contents

1. Instrumentation 2
2. Chemicals and Experimental Procedure 4
3. Supplementary Figures 9
4. Post-stability characterization 36
5. References 42

1. Instrumentation

Scanning electron microscopy (SEM) images were collected using FEI-Nova NanoSEM 650 equipped with an energy dispersive X-ray spectrometer (EDS) analyzer, operated at an accelerating voltage of 10 kV to investigate the morphology and elemental composition of the samples.

Transmission electron microscopy (TEM) combined with high-angle annular dark-field scanning transmission electron microscopy HAADF-STEM and EDX measurements were performed using a ThermoFisher Talos F200X (scanning) TEM equipped with a windowless SuperX energy dispersion detector (EDX). The catalyst material was dispersed in ethanol (supersonic bath) and drop cast onto holey carbon TEM grids prior to TEM studies. TEM studies were performed using a Thermofisher Talos 200X Scanning (S) TEM operated at 200 kV. Selected area diffraction patterns (SAED) and HRTEM images were captured using a CETA 4K CMOS camera. STEM (annular high angle dark field detector) was performed together with energy dispersive X-ray spectroscopy (EDX) (windowless 4-quadrant SuperX detector) to determine the local elemental composition. The HAADF-STEM measurements were performed on HAADF-STEM, JEOL JEM-ARM200F.

Powder X-ray diffraction (XRD): The crystal structural information of the samples was obtained by XRD (Bruker AXS D8 Advance) equipped with a Cu monochromator using Cu Kα radiation (wavelength λ = 1.5406 Å) operated at 40 kV and 40 mA.

Raman Spectroscopy: The Raman measurements were carried out using a confocal Raman microscope from Protrustech Co. Ltd. with a 50x objective and a laser power of 15 mW with a 633 nm laser beam.

Inductively coupled plasma optical emission spectrometry (ICP-OES): The ICP-OES samples were analyzed using an Agilent 5800 ICP-OES spectrometer equipped with an Agilent SPS4 autosampler (Agilent Technologies). The catalyst samples were dissolved in aqua regia (1:3 molar ratio of nitric acid (69%) and hydrochloric acid (37%)) and diluted by a factor of 100. The instrument was calibrated with a blank (Aristar ICP Nitric Acid Blank, VWR, Leuven, Belgium) and a multi-element standard for ICP-OES (Certipur ICP Multielement Standard Solution IV, Merck, Darmstadt, Germany) diluted to 10, 100 and 1000 µg/L for Sn and Zn in 5% HNO_3. The linearity of the calibration curves (R) was better than 0.9999 and their relative standard deviations were less than 1%.

X-ray photoelectron spectroscopy (XPS): XPS measurements were performed in a PHI Quantera SXM system equipped with monochromatized Al Kα exciting X-rays. The XPS spectra were calibrated using the C1s peak (284.5 eV).

X-ray absorption spectroscopy (XAS) analysis: XAS measurements (EXAFS and XANES) were performed at the 44A beamline of the National Synchrotron Radiation Research Centre (NSRRC), Hsinchu, Taiwan. The data were acquired in transmission mode using quick scanning enhanced X-ray absorption fine structure spectroscopy (QEXAFS) at a monochromator oscillation frequency of 1 Hz. The Sn foil was measured using a reference ion chamber for energy calibration. The samples were prepared as 13 mm pellets. For each sample, 40 mg of the respective samples (**Sn/N-C-M**, **Sn/N-C-D**, **Sn/N-C-T** and **Sn/N-C**, and reference SnO_2) were diluted with 50 mg of cellulose. Sn foil and SnO_2 were used as the references. The edge energy was determined by the maximum of the first derivative of the first XANES peak. Multiple scans were performed for each sample and averaged to obtain a better signal-to-noise ratio.

***In-situ/operando* XAS measurement**: To investigate the local structure, coordination environment, and electronic configuration of the active sites in the **Sn/N-C-M** catalyst under operating conditions, *in-situ*/*operando* XAS measurements were performed. A custom-built electrochemical cell was used, comprising a catalyst-coated carbon fiber paper as the working electrode, a graphite rod counter electrode, and an Ag/AgCl (3 M KCl) reference electrode. The electrolyte was 0.1 M $KHCO_3$ solution, continuously purged with flowing CO_2 to maintain reaction conditions. XAS data were collected at the Sn K-edge in fluorescence mode at the Quick EXAFS beamline (TPS-44A) of the National Synchrotron Radiation Research Centre (NSRRC), Taiwan. Energy calibration was performed using Sn foil as a reference standard. The QEXAFS beamline enabled rapid data acquisition, capturing two spectra per second, allowing for dynamic monitoring of catalyst state changes during electrochemical reactions. To elucidate the coordination structure and oxidation state evolution of the active sites in **Sn/N-C-M**, XAS measurements were conducted for the dry electrode, at open circuit potential (OCP, pre-electrolysis), and during electrolysis under applied potentials ranging from -0.1 V to -1.2 V versus RHE. Spectra were recorded after 15 minutes of potential application to ensure steady-state conditions.

***In-situ* attenuated total reflection Fourier-transform infrared spectroscopy (ATR-FTIR) study**: *In-situ* ATR-FTIR studies were performed in the potential range from 0 to -1.3 V vs. RHE in CO_2-saturated 0.1 M $KHCO_3$ using a flow electrolytic cell. In-situ ATR-FTIR was performed using a **Sn/N-C-M** catalyst coated on a carbon substrate as the working electrode, with an Ag/AgCl reference electrode and nickel foam as the counter electrode. The cathode and anode compartments are separated by a Nafion membrane. The electrodes were prepared with a catalyst ink obtained by dissolving 10 mg of the catalyst in a mixture of isopropanol (950 µl) and Nafion solution (50 µl, 5 wt% in a mixture of lower alcohols) and sonicating for 30 minutes. Subsequently, 200 µl of the ink was dropped onto a carbon substrate (surface area = 2 cm^2). The electrochemical flow cell contains a CO_2-saturated 0.1 M $KHCO_3$ solution as electrolyte. During the measurements, different potentials were applied to the working

electrode, and the spectra were recorded from open-circuit potential (OCP) to -1.3 V versus RHE after 30 min of electrolysis under continuous electrolyte flow.

2. Chemicals and Experimental Procedure

Chemicals:

Zinc nitrate hexahydrate ($Zn(NO_3)_2·6H_2O$), 2-methyl imidazole, tin (II) chloride dihydrate ($SnCl_2·2H_2O$), potassium hydrogen carbonate ($KHCO_3$), methanol (CH_3OH), dimethyl sulfoxide (DMSO), carbon dioxide (CO_2 and $^{13}CO_2$ isotopically labelled), Nafion solution (50 µL, 5 wt.% in a mixture of lower alcohols), and deuterated water (D_2O) were obtained from Sigma-Aldrich. All chemicals used in this study were of analytical grade and were used without further purification. Deionized water (DI) was used in all experiments.

Synthesis of the ZIF-8 precursor. In a typical procedure, 2-methyl imidazole (16 mmol) was dissolved in methanol (15 mL) with stirring. In a separate vessel, $Zn(NO_3)_2·6H_2O$ (4 mmol) were dissolved in 30 ml methanol and sonicated for 15 minutes to obtain a transparent solution. Both solutions were then mixed and stirred for 10 minutes at room temperature. The resulting mixture was transferred to an airtight container and kept at a fixed temperature of 35 °C for 12 h. Finally, the resulting crystalline white precipitates were collected by centrifugation, washed five times with methanol and dried overnight at 42 °C in a vacuum.

Synthesis of the N-C. For the synthesis of N-C, the as-obtained ZIF-8 powders were placed in a quartz boat and kept for 3h at 950 °C in a tube furnace with a heating rate of 5 °C min^{-1} under flowing argon gas. Afterwards, the sample designated as N-C, which contained Zn sites, was obtained by cooling to room temperature. The resulting N-C sample was then leached for 8 h under reflux in a 2 M H_2SO_4 solution (95% H_2SO_4) to effectively remove most of the coordinated Zn sites. Leaching of N-C under aggressive acid reflux conditions was significantly more effective in removing Zn sites than acid leaching at room temperature.

Synthesis of the Sn/N-C, Sn/N-C-M, Sn/N-C-D, and Sn/N-C-T. For the synthesis of Sn/N-C, 100 mg of N-C powder was dispersed in 100 ml of methanol containing 100 mg of $SnCl_2·2H_2O$. This corresponded to a Sn(II) content of about 34 wt.% in the mixture of N-C and Sn. This mixture was refluxed for 8 h under a nitrogen atmosphere with constant stirring and then thoroughly washed with methanol and deionized water to ensure complete removal of the physisorbed Sn ions. This tin-coordinated catalyst (**i-Sn/N-C**) was then dried in a vacuum oven at 42 °C overnight. This **i-Sn/N-C** was then mixed separately with melamine, dicyandiamide (DCDA) and thiourea as additional nitrogen sources in an **i-Sn/N-C**:additive weight ratio of 1:10 and thoroughly ground for 15 minutes before being subjected to the mechanochemical treatment steps by ball milling. Afterwards, the mixed samples were ball-milled at 600 rpm for 2 h using a planetary ball mill (Fritsch Pulverisette 7 Classic) equipped with zirconia jars and zirconium oxide balls (3 mm diameter). The resulting mixtures were then activated at 800 °C for 3 h in a tube furnace under an argon atmosphere with a heating rate of 3 °C min^{-1},

yielding the final **Sn/N-C-M**, **Sn/N-C-D**, and **Sn/N-C-T** samples, respectively. As a control, ball milling of **i-Sn/N-C** was also carried out under identical conditions to produce **Sn/N-C**, without addition of nitrogen sources (melamine, DCDA, and thiourea). The catalysts obtained were used without further treatment for catalytic applications and for characterization.

Preparation of the catalyst ink: the catalyst ink was prepared by dispersing 5 mg of the respective catalyst (**Sn/N-C-M**, **Sn/N-C-D**, **Sn/N-C-T, Sn/N-C** or **N-C**) in a mixture of isopropanol (950 µL) and Nafion solution (50 µL, 5 wt.% in a mixture of lower alcohols) and sonicating for 30 min. Electrodes were prepared by drop-casting 100 µL of the ink on carbon paper (surface area = 1 cm^2). The resulting modified electrodes were air-dried resulting in a catalyst loading of 500 µg cm^{-2}.

Electrocatalytic CO_2 reduction measurement: The electrochemical measurements were carried out at room temperature and ambient pressure in an H-type cell using a Ametek PMC 2000A potentiostat. A three-electrode cell configuration was employed using a carbon paper electrode as working electrodes, saturated calomel (SCE, Hg/Hg_2Cl_2) as reference electrode, and a graphite rod as counter electrode. Anodic and cathodic compartments were separated by a Nafion 117 membrane. Cyclic voltammetry (CV) and linear sweep voltammetry (LSV) measurements were performed in Ar- or CO_2-saturated 0.1 M aqueous $KHCO_3$ (pH 6.9) at a scan rate of 50 and 5 mV s^{-1}, respectively, without IR corrections. All potentials were converted to the reversible hydrogen electrode (RHE) scale according to the Nernst equation:

E (vs. RHE) = E (vs. SCE) + 0.242 V + 0.0591 × pH.

Electrocatalytic CO_2 reduction in a flow cell: The electrocatalytic CO_2RR measurements were also performed on an electrochemical Ametek PMC 2000A3 workstation connected to a flow cell. The CO_2RR performances were recorded in a three-electrode with Ni-foam as the counter electrode and saturated Ag/AgCl as the reference electrode. For electrocatalytic CO_2 reduction measurements in a flow cell and full-cell CO_2RR measurements using a membrane electrode assembly (MEA), catalyst inks were prepared by dispersing 10 mg of the respective catalyst in the same solvent mixture (950 µL isopropanol and 50 µL Nafion solution), followed by sonication. The resulting catalyst ink (400 µL) was then sprayed onto one side of a carbon paper (2 cm× 2 cm) to obtain a catalyst loading of 75.1 µg cm^{-2}. During CO_2RR measurements, high-purity CO_2 gas was continuously fed directly to the catalyst layer through the gas diffusion layer of the GDE at a controlled flow rate, ensuring efficient gas-solid-liquid triple-phase boundary formation. Anodic and cathodic compartments were separated by a Nafion 117 membrane, and a peristaltic pump was used to circulate an aqueous solution of 1 M KOH and 0.1 M $KHCO_3$, respectively, at a rate of 10 ml/min. All potentials applied in this study were converted to the RHE scale using the following equation:

E (vs. RHE) = E (vs. Ag/AgCl) + 0.197 V + 0.0591 × pH.

Full-cell CO_2RR measurements using a membrane electrode assembly (MEA)

To evaluate the operational stability of the catalyst under continuous device-level conditions, electrochemical CO_2 reduction measurements were conducted in a MEA-type flow electrolyzer using a bipolar membrane (BPM) in a two-electrode configuration. The BPM separated the cathodic and anodic compartments while maintaining distinct local pH environments at the two electrodes. The cathode catalyst ink was prepared by dispersing the synthesized catalyst in a mixed solvent containing isopropanol, deionized water, and 5 wt% Nafion solution, followed by ultrasonication for 30 min to obtain a homogeneous suspension. The resulting ink was coated onto a carbon-based gas diffusion layer (GDL) with a catalyst loading of approximately 75.1 μg/cm^2. For full-cell electrolysis, the **Sn/N-C-M** catalyst-coated GDL was used as the cathode, while commercial IrO_2 supported on a gas diffusion electrode served as the anodic oxygen evolution catalyst. A commercial BPM was immersed in deionized water prior to cell assembly and positioned between the cathode and anode compartments with the anion-exchange layer facing the cathode side. During electrolysis, 0.1 M $KHCO_3$ catholyte and 1 M KOH anolyte were continuously circulated at a flow rate of 10 mL/min using a peristaltic pump. Continuous CO_2 electrolysis was performed galvanostatically at a current density of 100 mA cm^{-2} for 100 h under continuous CO_2 flow. Liquid products were collected every 24 h and quantified to determine the Faradaic efficiency for formate production, while the full-cell voltage was monitored throughout the electrolysis period to evaluate operational stability. Formate detected in both the catholyte and anolyte compartments was included in the total Faradaic efficiency calculation to account for crossover through the bipolar membrane.

Product and efficiency analyses: The product analysis and faradaic efficiency of electrocatalytic CO_2 reduction were measured by chronoamperometry at a fixed potential in an H-cell separated by a Nafion 117 membrane and chronopotentiometry at a fixed current density in a flow electrolytic cell. The liquid products were quantified by nuclear magnetic resonance (NMR) spectroscopy (Bruker Avance 400 multicore NMR spectrometer). A 1D sequence solvent suppression method using excitation sculpting with gradients (zgesgp) was used for the liquid product analysis using the following acquisition parameters (NS = 512, TD (1TD and 2TD) = 32768, O1P = 4.702 ppm, O2P = 4.700 ppm, DE = 20 μsec, 1SW and 2SW = 15.6128 ppm, and a relaxation delay between pulses of D1 = 8 s was used to allow complete relaxation of the protons), and the same spectral acquisition parameters were used for all spectra to ensure complete relaxation and quantification. For sample preparation for NMR measurement, a 600 μl electrolyte sample was mixed with 50 μl internal standard solution. The internal standard solution was prepared by mixing 13 ml D_2O with 5.0 μl DMSO, which corresponds to 5.44 mM DMSO in D_2O. Calibration curves were obtained by measuring known formate concentrations and internal standard solutions.

The peaks were quantified by integrating the respective peak areas. The relative peak area was calculated as follows:

$$\text{Relative peak area ratio (formate)} = \frac{\text{Singlet peak area at 8.3 ppm (HCOO}^-\text{)}}{\text{Singlet peak area at 2.51 ppm (DMSO)}}$$

The gaseous products (H_2, CH_4, CO, and C_2H_4) were quantified by gas chromatography (Shimadzu GC2030) equipped with a barrier ionization discharge (BID) detector. High-purity helium (He, 99.999%) was used as carrier gas.

The faradaic efficiencies for a product *i*, FE_i were calculated based on the following equation:

$$\text{FE}_i = \frac{\alpha_i n_i F}{Q} \; x \; 100\%$$

where α_i is the number of electrons required to produce one molecule of *i*, n_i is the number of moles of product *i*, F is the Faraday constant (96485 C mol^{-1}), and Q is the electric charge passed during the experiment. α = 2 for H_2, CO, and formate, 8 for CH_4 and acetate, and 12 for C_2H_4, ethanol.

Determination of the electrochemical double layer capacitance (C_{dl}): The C_{dl} of the samples were estimated from the CV curves. The CV measurement was performed in the same electrochemical cell where no Faraday processes took place. CV curves between 0.78 and 1.06 V were then recorded against RHE at different scan rates (2 - 10 mV s^{-1}). Subsequently, the current densities were extracted at 0.924 V vs. RHE as a function of scan rates, resulting in a slope for each sample. The slope of the plot corresponds to the capacitance of the measured samples.

The half-cell cathodic energy efficiency (EE%) of the formate was estimated using the following equation:[1]

$$\text{EE}_{\text{formate}} (\%) = \frac{(1.23 - E_{formate}) \; x \; FE_{formate}}{1.23 - E_{applied}}$$

Where E_{applied} is the potential used in the experiment, FE_{formate} is the measured FE of formate in percentage, and E_{formate} = -0.196 V vs. RHE for CO_2RR. The overpotential of oxygen evolution is assumed to be zero. The EE was calculated at an applied potential of −1.3 V vs. RHE, corresponding to the optimum potential for CO_2 reduction to formate in the H-type electrolysis cell. For the flow electrolyzer, the EE was evaluated at a current density of 100 mA cm^{-2}, using the corresponding cell operating potential.

The full-cell EE for formate production was calculated according to:

$$\text{EE}_{\text{formate}} (\%) = \frac{(1.23 - E_{formate}) \; x \; FE_{formate}}{V_{cell}}$$

where FE_{formate} is the Faradaic efficiency for formate (expressed as a percentage), and V_{cell} is the experimentally measured full-cell voltage under operating conditions.

Apparent TOF Calculation Details: Apparent TOFs (h^{-1}) for the electroreduction of CO_2 to formate production were determined using the equation below:[2]

$$\text{Formate production rate, } n_{formate} = \frac{Q \text{ x } FE_{formate}}{2F}$$

$$\text{TON} = \frac{n_{formate}}{N}$$

$$\text{N} = (\frac{\text{Weight of Sn on electrode}}{119}) * N_A$$

$$\text{Apparent TOF} = \frac{\text{TON}}{t}$$

$$\text{Apparent TOF} = \frac{J \text{ x } FE_{formate} \text{ x } N_A}{2F \; x \; N}$$

Where N is the number of active sites per cm^2, Q is the total charge, J and FE (formate) are the current density and Faradic efficiency of formate formation, respectively. F is Faraday constant (96485 C mol^{-1}), n is mole of formate product, t is the electrolysis duration, and N_A is Avogadro's constant ($6.022 * 10^{23}$ mol^{-1}). Based on formate formation in H-type electrolysis cell at -1.3 V vs. RHE, FE (formate) is 82.5%; total current density is ~29.73 mA cm^{-2}; loading amount of reactive catalyst on carbon paper is 37.5 µg cm^{-2}; Sn species content in **Sn/N-C-M** is 7.5 wt.%. In a flow electrolytic cell, the optimum FE of formate is 92.5% at the current density of 100 mA cm^{-2}.

Computational details: All DFT calculations in the present work were performed by the Vienna Ab initio Simulation Package (VASP) 5.4.4 code[3] with the Perdew-Burke-Ernzerhof (PBE) functional.[4] The ion-electron interaction was described by the projector augmented wave (PAW) method[5] with a kinetic energy of 500 eV. To simulate CO_2RR to describe the formation of formate and CO on the proposed catalysts, we constructed the model structures deduced by the experimental observations. XPS analysis revealed that pyridinic-N and pyrrolic-N are the dominant nitrogen configurations in the carbon matrix. In addition, the local structures of Sn are examined by XAS analysis indicated that Sn is coordinated by four nitrogen atoms (Sn-N_4). Because Sn remained atomically dispersed while sulfur doping, we constructed two models of Sn-N_4 sites with the different N environments, namely pyridinic-N-coordinated Sn-N_4 (denoted as Sn-N_4-Py) and pyrrolic-N-coordinated Sn-N_4 (denoted as Sn-N_4-Pr). Sulfur-doped structures, including graphitic-S-modified (Sn-N_4-gS) and curved-S-modified (Sn-N_4-cS) models, were also constructed. The optimized structures are shown in **Supplementary Figure S47**. A Monkhorst-Pack k-point mesh with a size of 3 × 3 × 1 and the spin polarization was applied for the calculations. In addition, the DFT-D3 correction method

in Grimme's scheme[6-7] was also used to accurately describe the long-range vdW interactions. All the calculations were carried out until the total energy and force were less than 10^{-5} eV per atom and 0.05 eV Å^{-1}, respectively.

We first investigated the adsorption of CO_2 on the Sn single-site catalyst models. The Gibbs free-energy diagram for CO formation was then constructed based on the following elementary steps of the CO_2 reduction reaction:

$$* + CO_2 \rightarrow {}^*CO_2 \qquad \text{(S1)}$$

$$* + CO_2 \rightarrow {}^*OCO \qquad \text{(S2)}$$

The adsorbed CO_2 can adopt either a C-bound (*CO_2) or O-bound (*OCO) configuration on the single-atom site. These two adsorption configurations initiate two distinct CO_2RR pathways. Protonation of the C-bound intermediate forms *COOH, which is the key intermediate for CO formation. A subsequent proton-coupled electron transfer yields adsorbed *CO:

$$^*CO_2 + H^+ + e^- \rightarrow {}^*COOH \qquad \text{(S3)}$$

In contrast, protonation of the O-bound intermediate forms *OCHO, which is the key intermediate for formate formation. Accordingly, the free-energy diagrams for formate production are based on the *OCHO pathway, whereas those for CO production are based on the *COOH pathway.

$$^*OCO + H^+ + e^- \rightarrow {}^*OCHO \qquad \text{(S4)}$$

Supplementary Figures

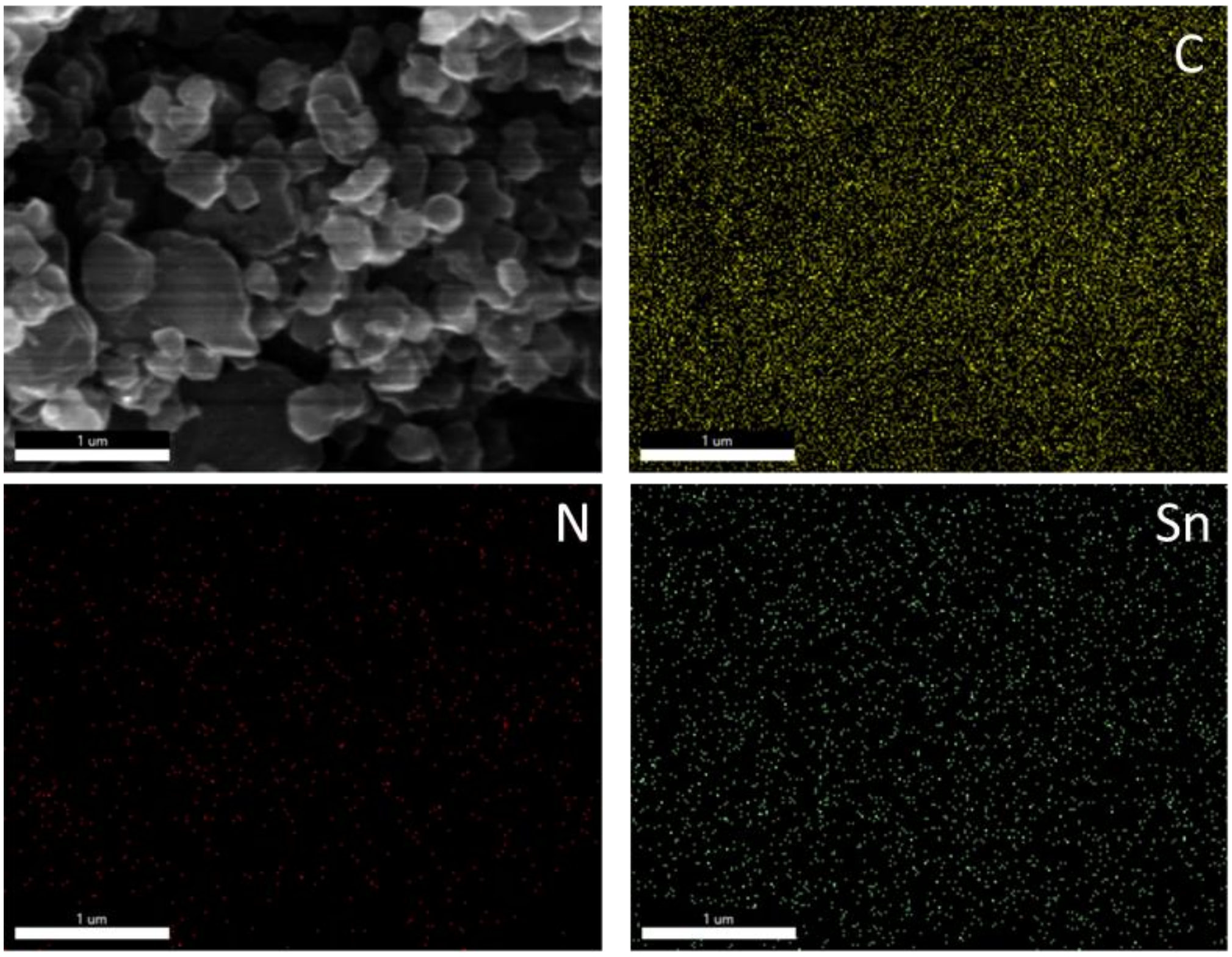

Figure S1. SEM image and the corresponding C, N, and Sn EDS elemental mappings of single-site **Sn/N-C-M** catalyst.

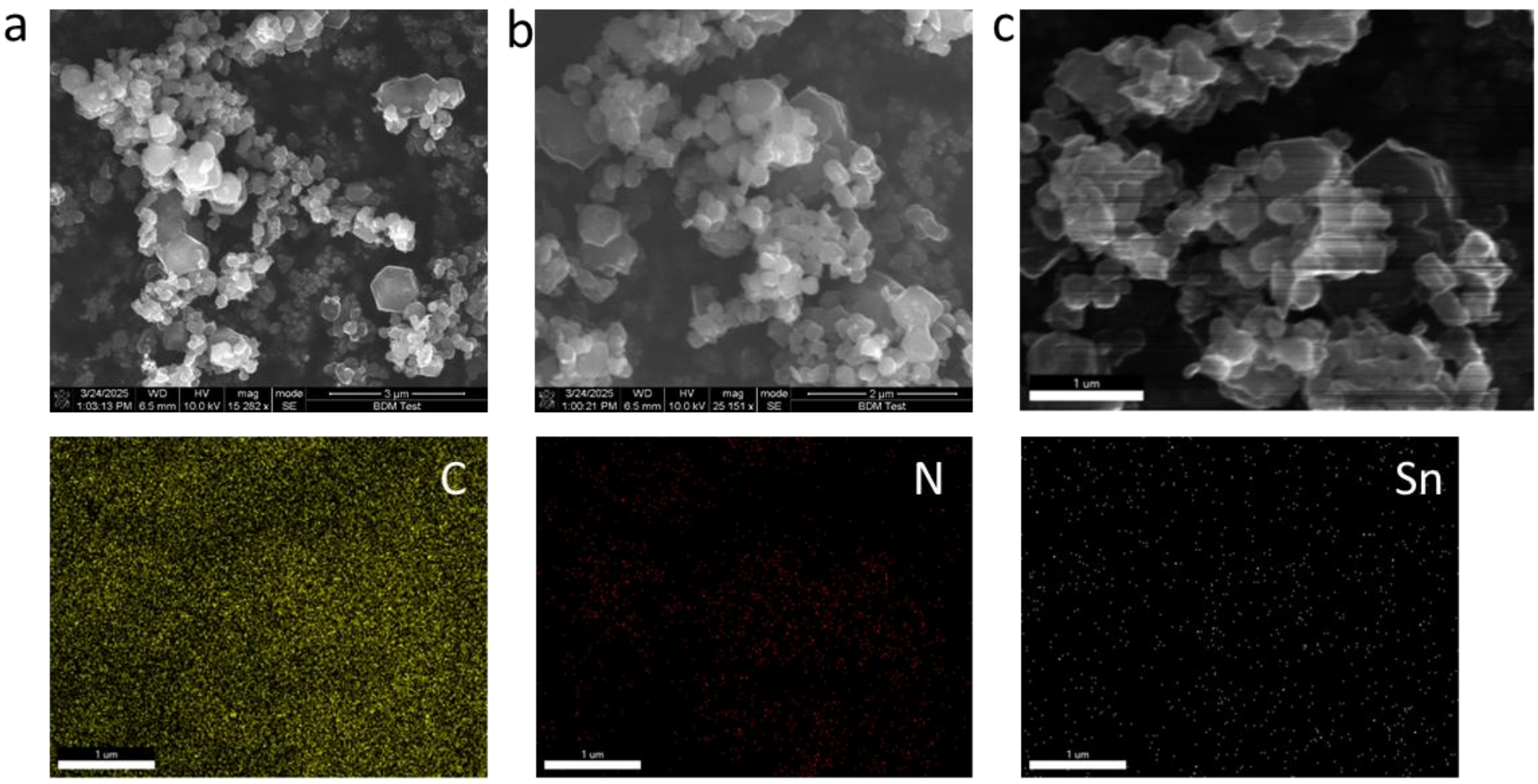

Figure S2. SEM image and the corresponding C, N, and Sn EDS elemental mappings of single-site **Sn/N-C-D** catalyst.

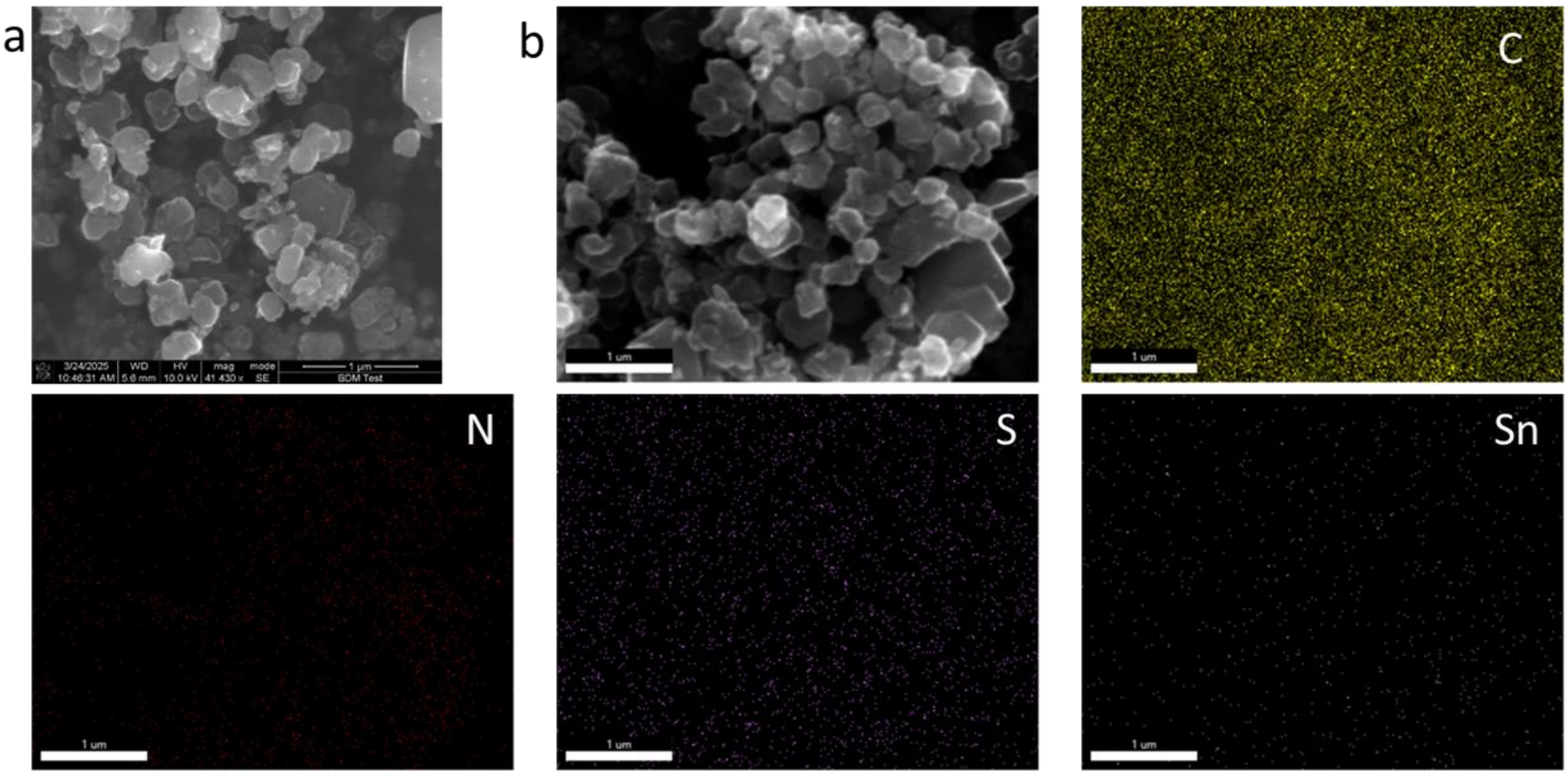

Figure S3. SEM image and the corresponding C, N, S, and Sn EDS elemental mappings of single-site **Sn/N-C-T** catalyst.

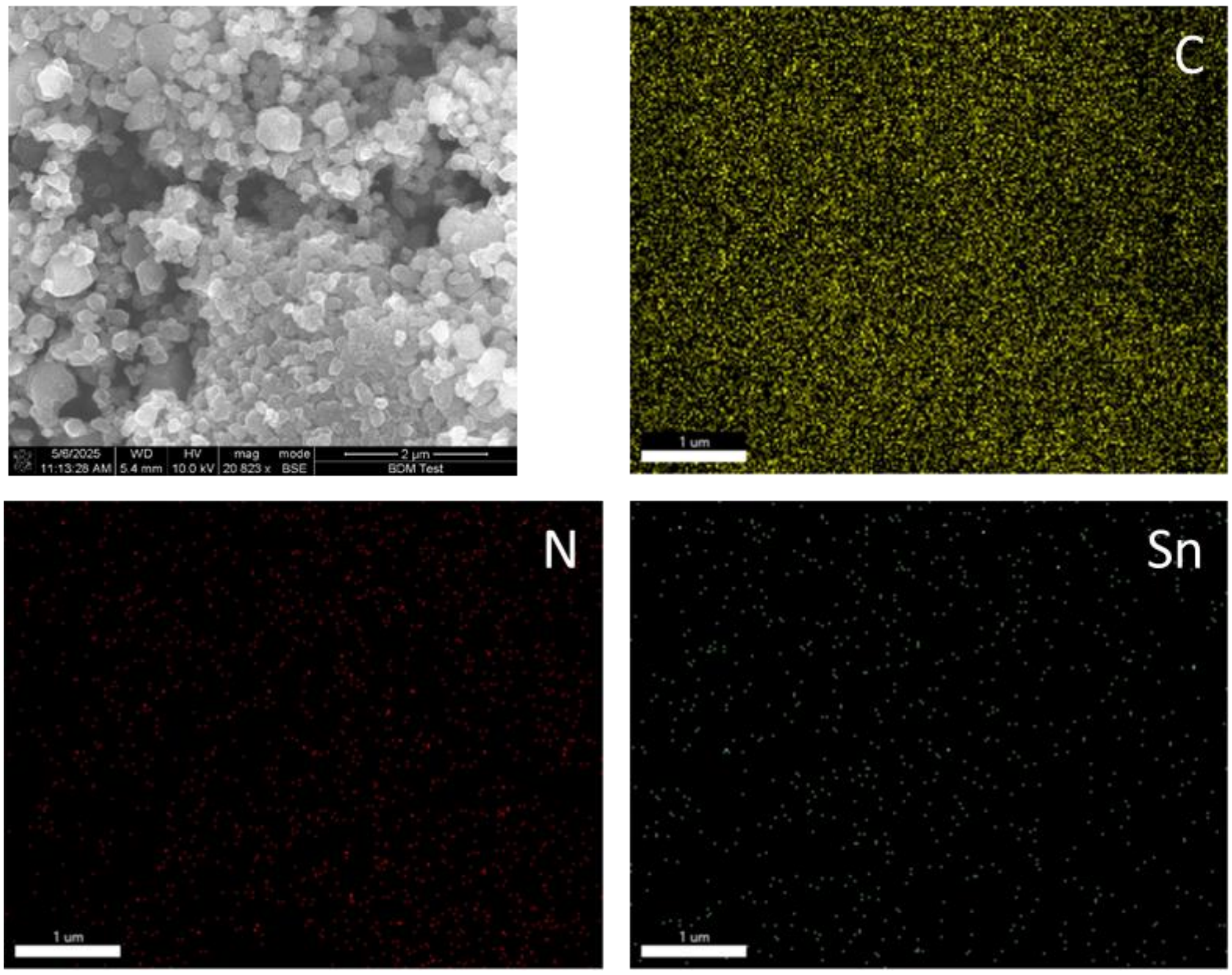

Figure S4. SEM image and the corresponding C, N, and Sn EDS elemental mappings of single-site **Sn/N-C** catalyst.

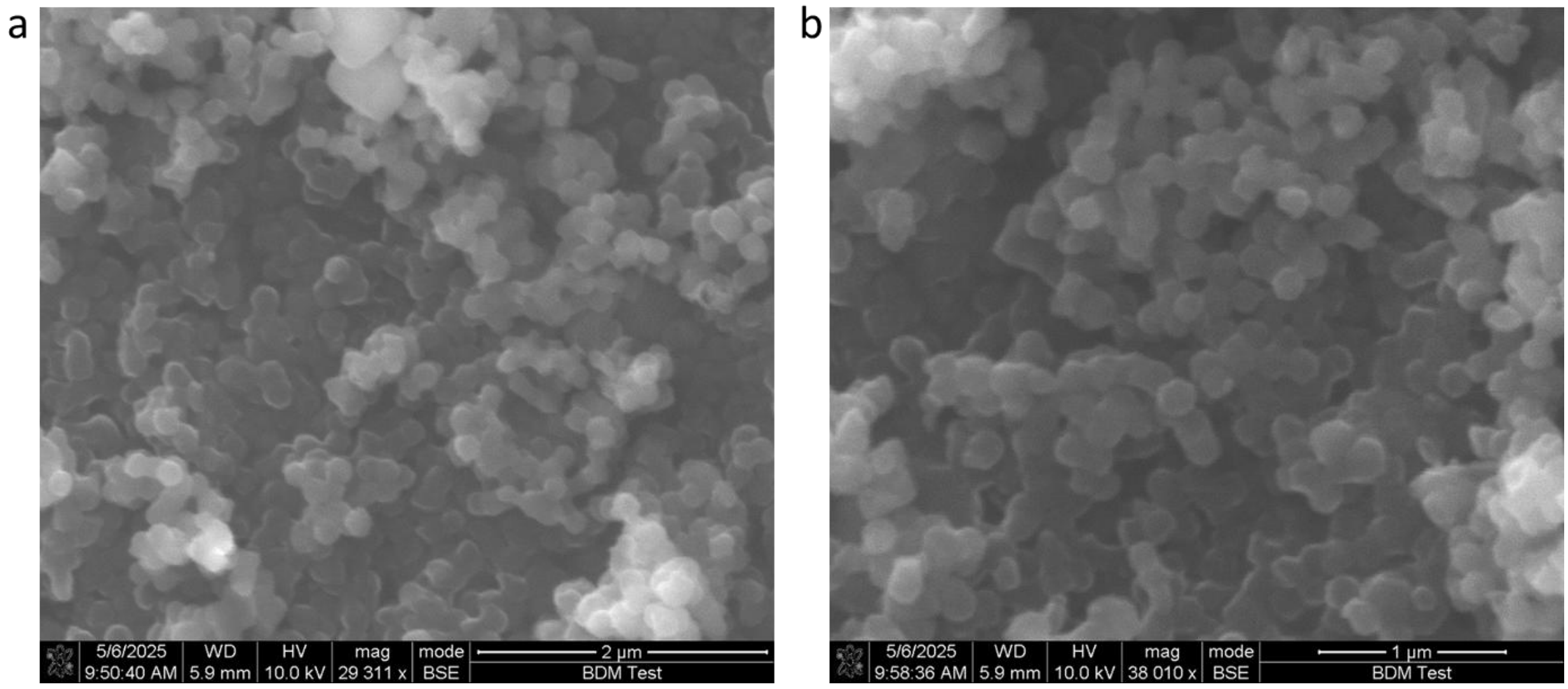

Figure S5. SEM images of the **N-C** support at different magnifications.

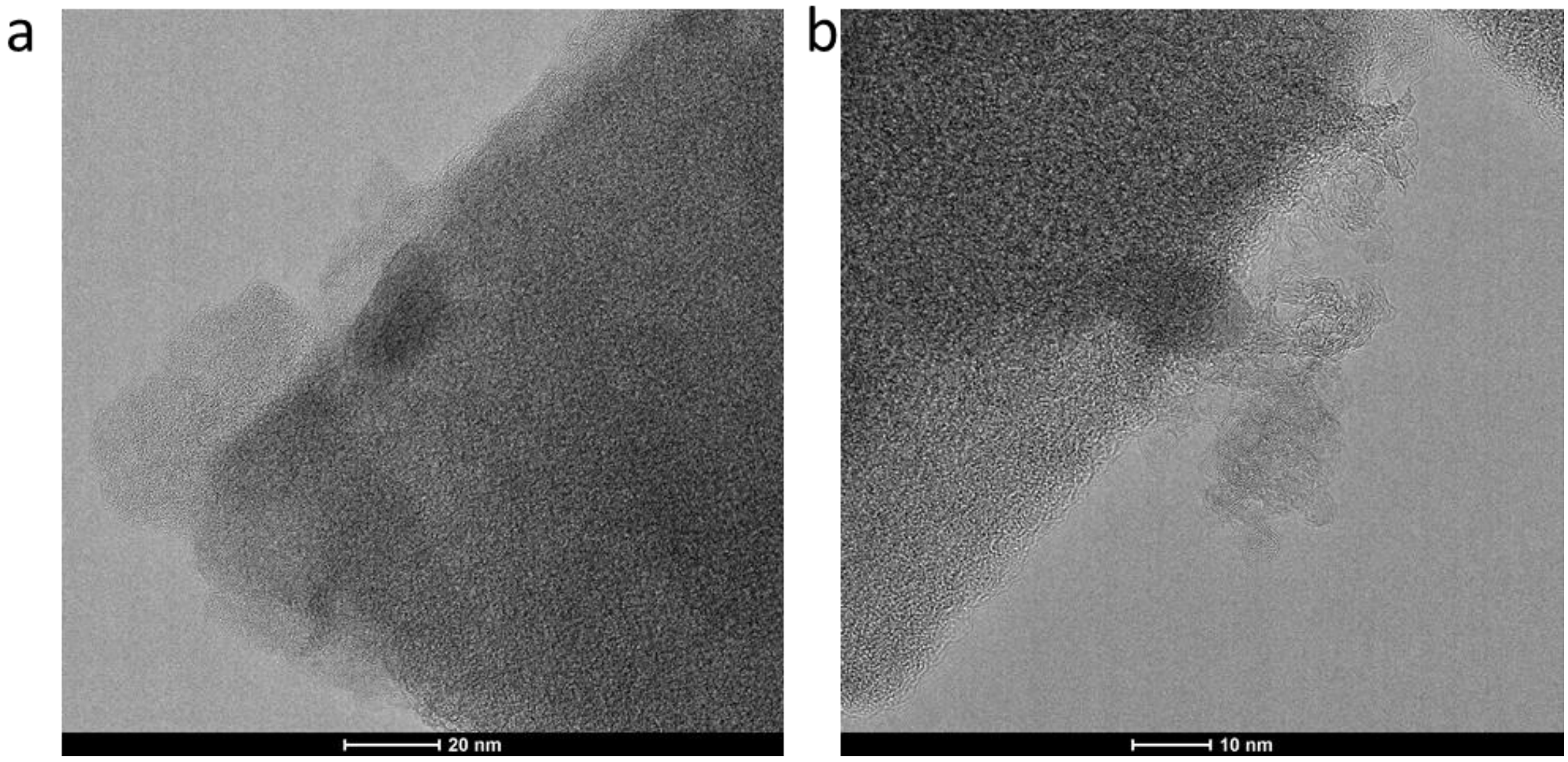

Figure S6. HR-TEM images of the **Sn/N-C-M** at different magnifications showing the amorphous structure.

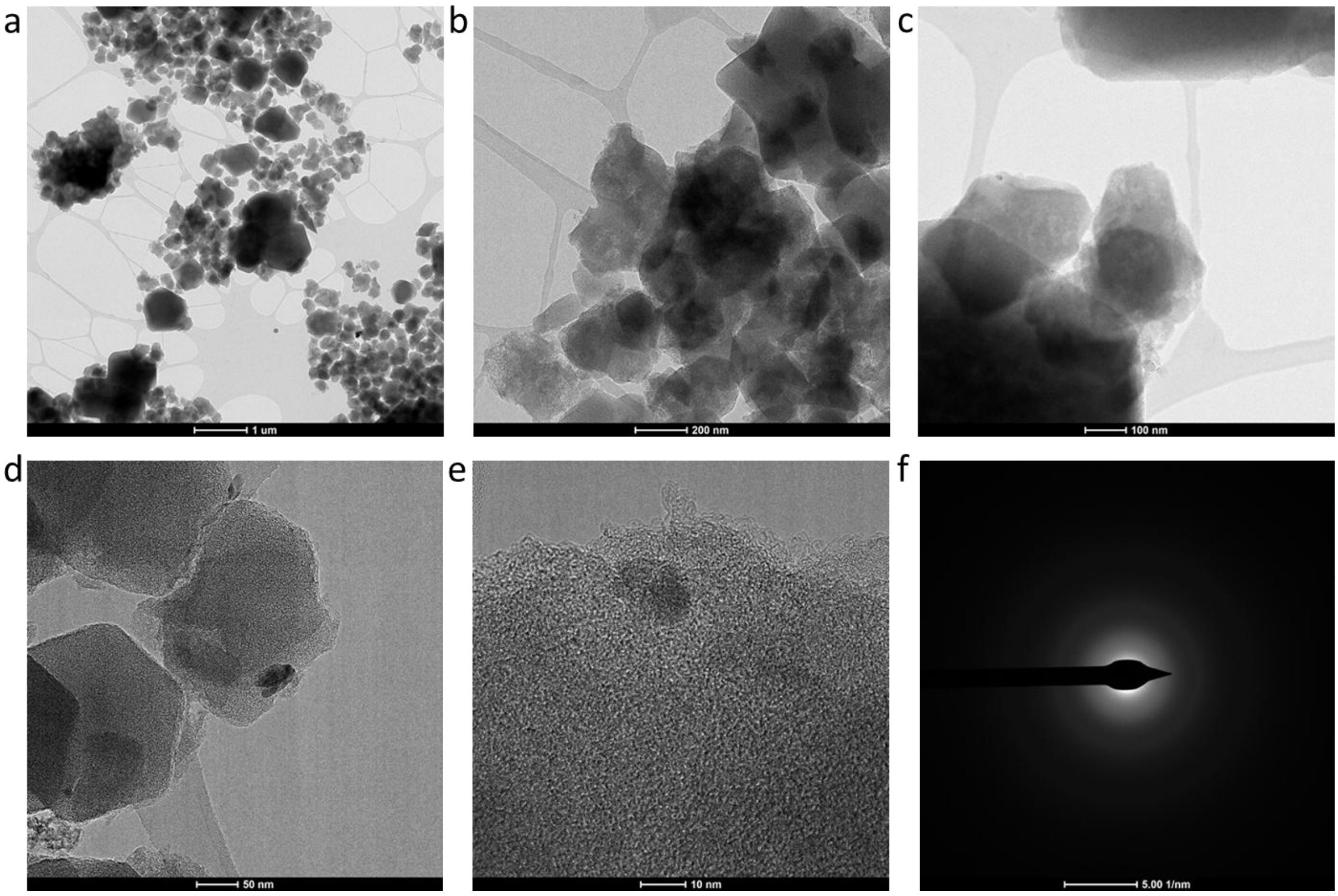

Figure S7. (a, b, c) TEM and (d, e) HR-TEM images of the **Sn/N-C-D** at different magnifications together with a SAED pattern (f).

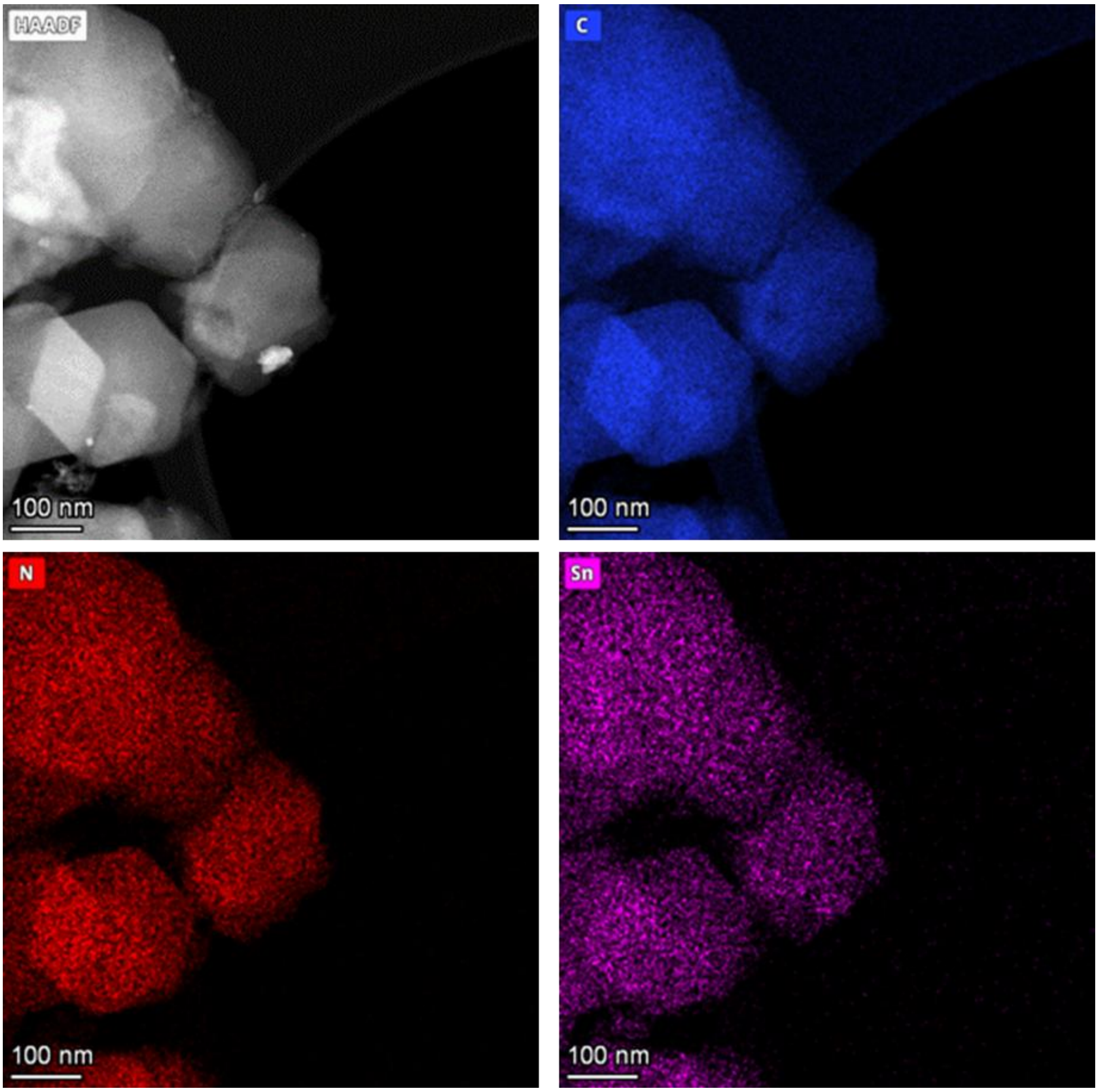

Figure S8. HAADF-STEM image and the corresponding C, N, and Sn EDS elemental mappings of **Sn/N-C-D**.

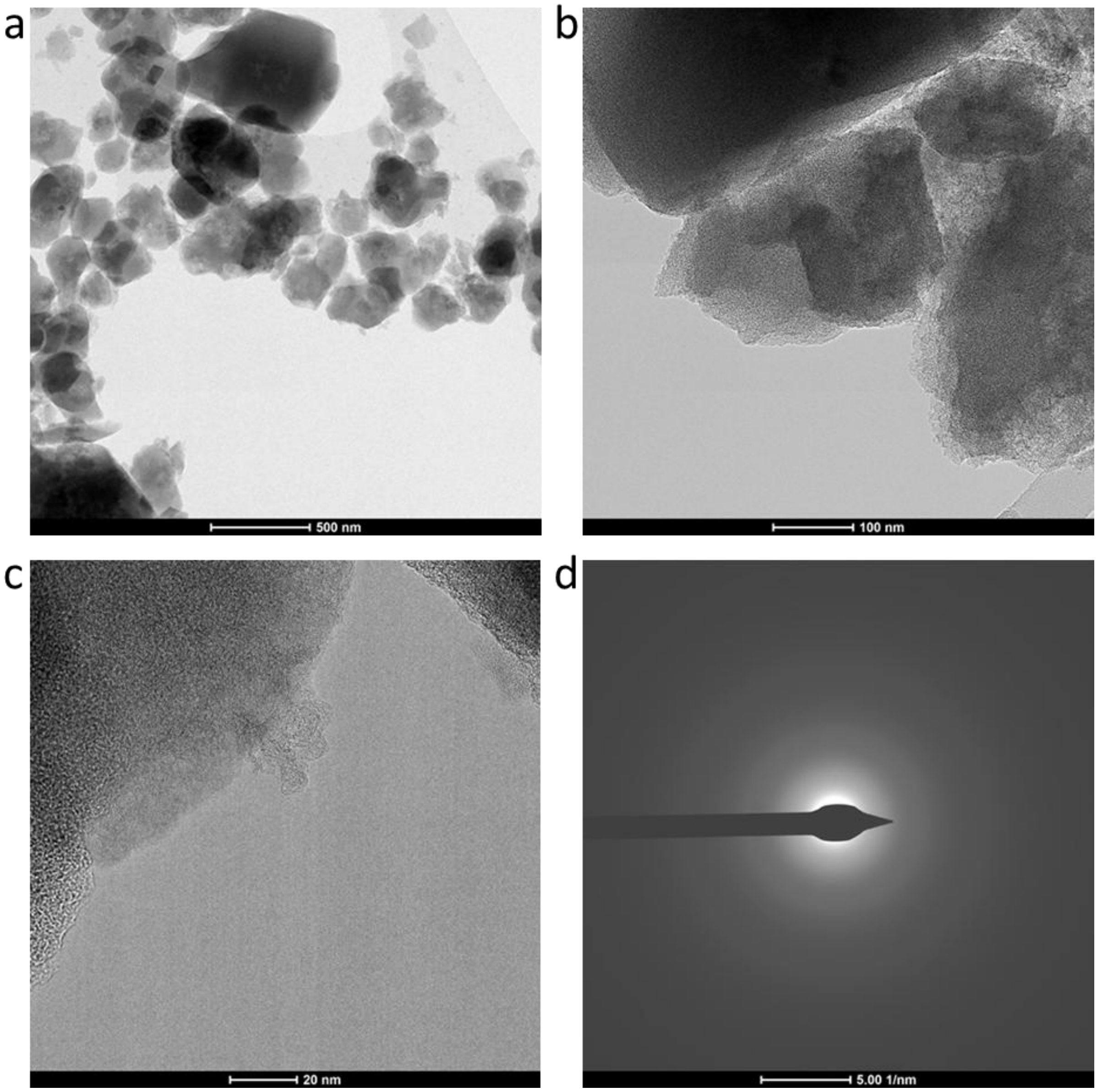

Figure S9. (a) TEM image overview and (b,c) HR-TEM images of the **Sn/N-C-T** at different magnifications together with a SAED pattern (d).

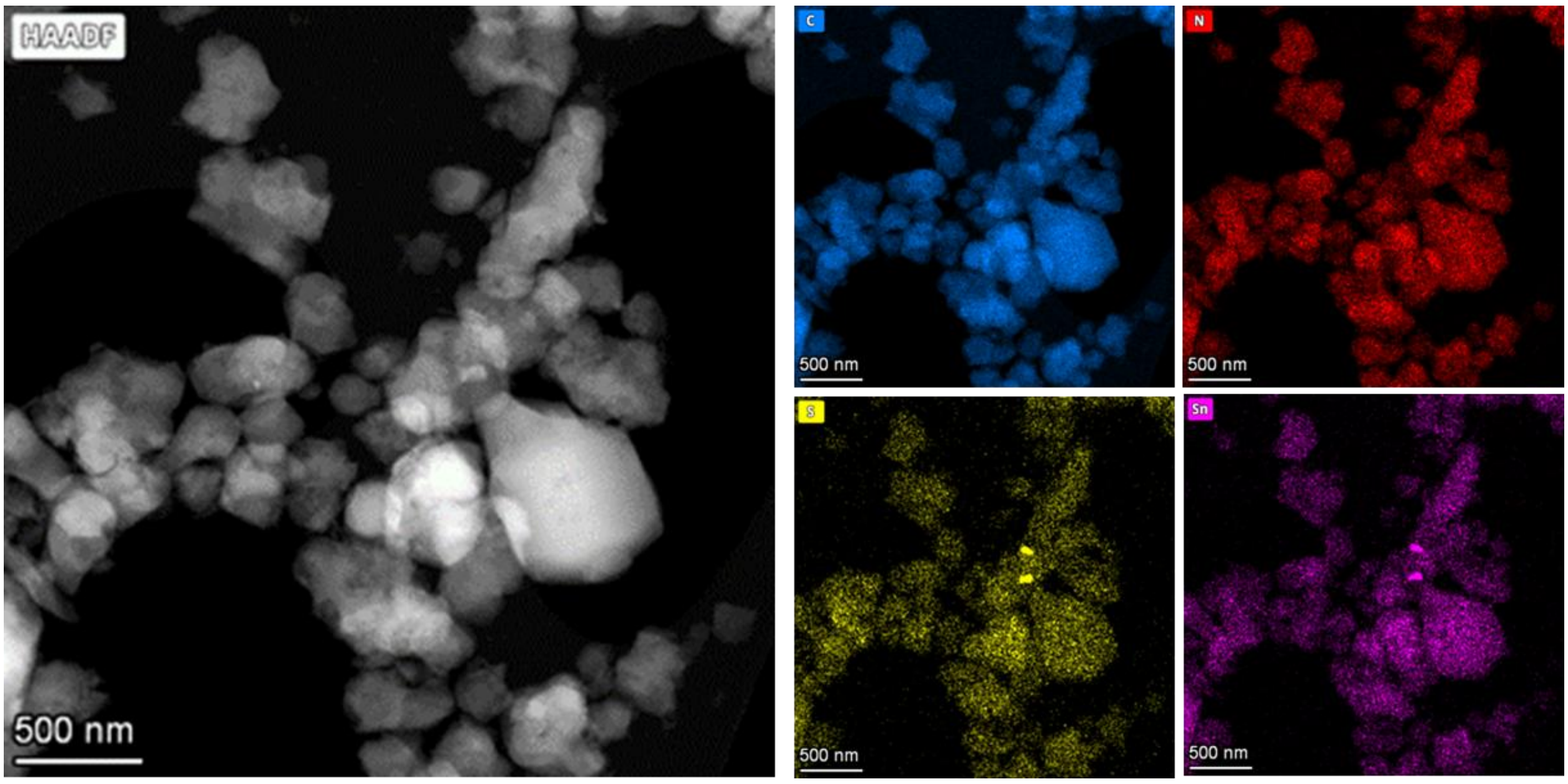

Figure S10. HAADF-STEM image and the corresponding C, N, S, and Sn EDS elemental mappings of **Sn/N-C-T**.

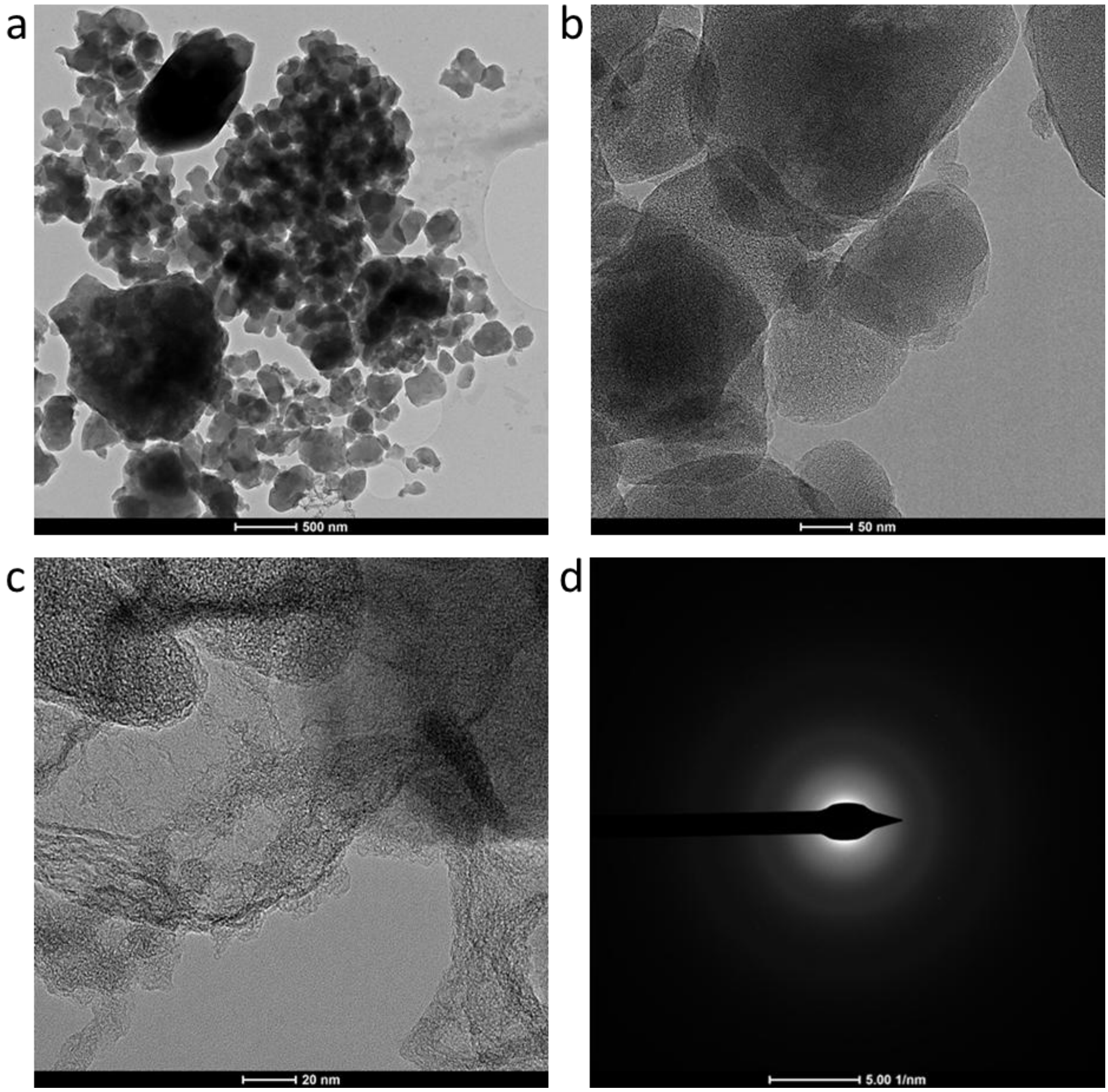

Figure S11. (a) Overview TEM image (b,c) HR-TEM images of the **Sn/N-C** at different magnifications together with a SAED pattern (d).

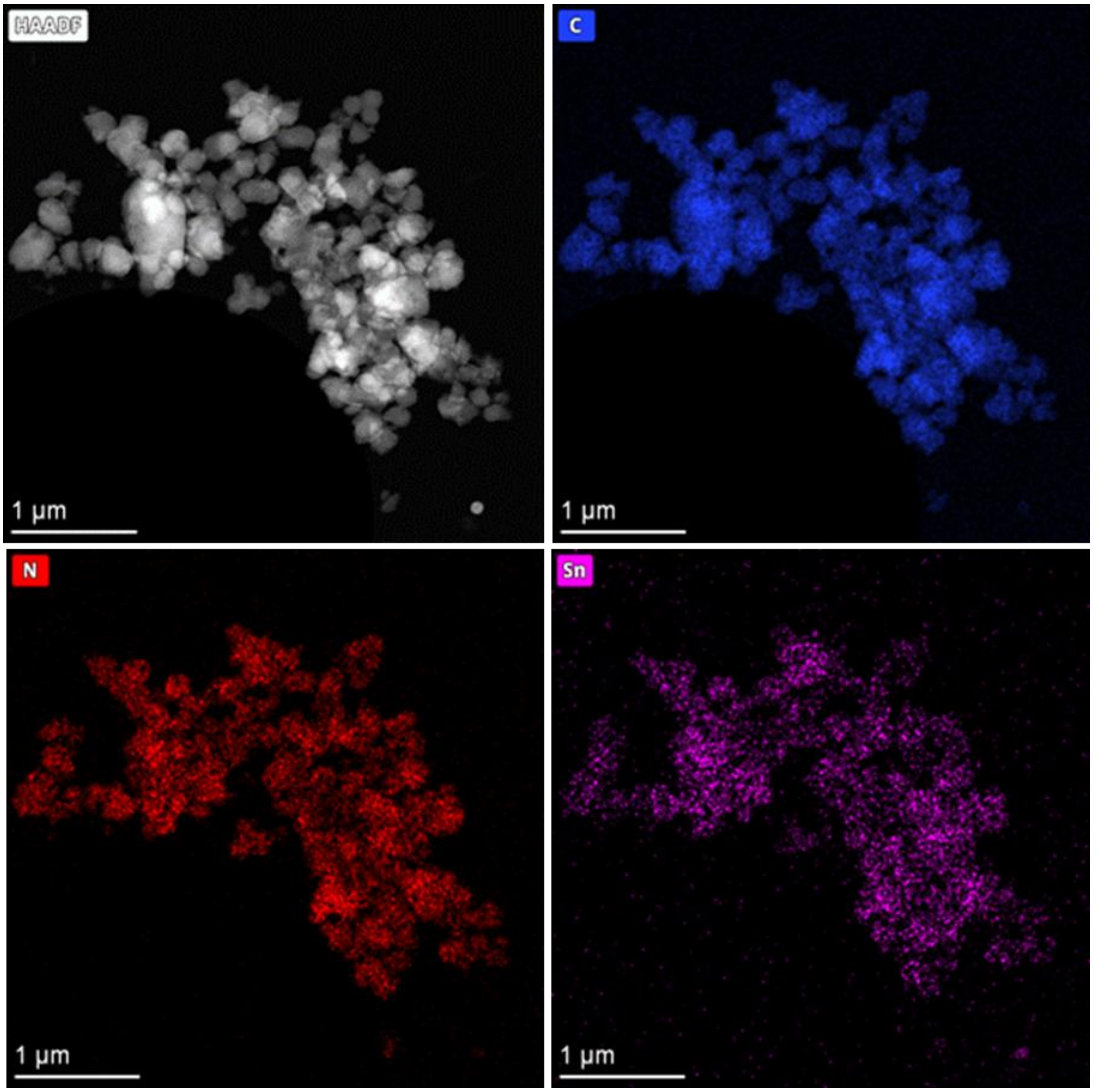

Figure S12. HAADF-STEM image and the corresponding C, N, and Sn EDS elemental mappings of **Sn/N-C**.

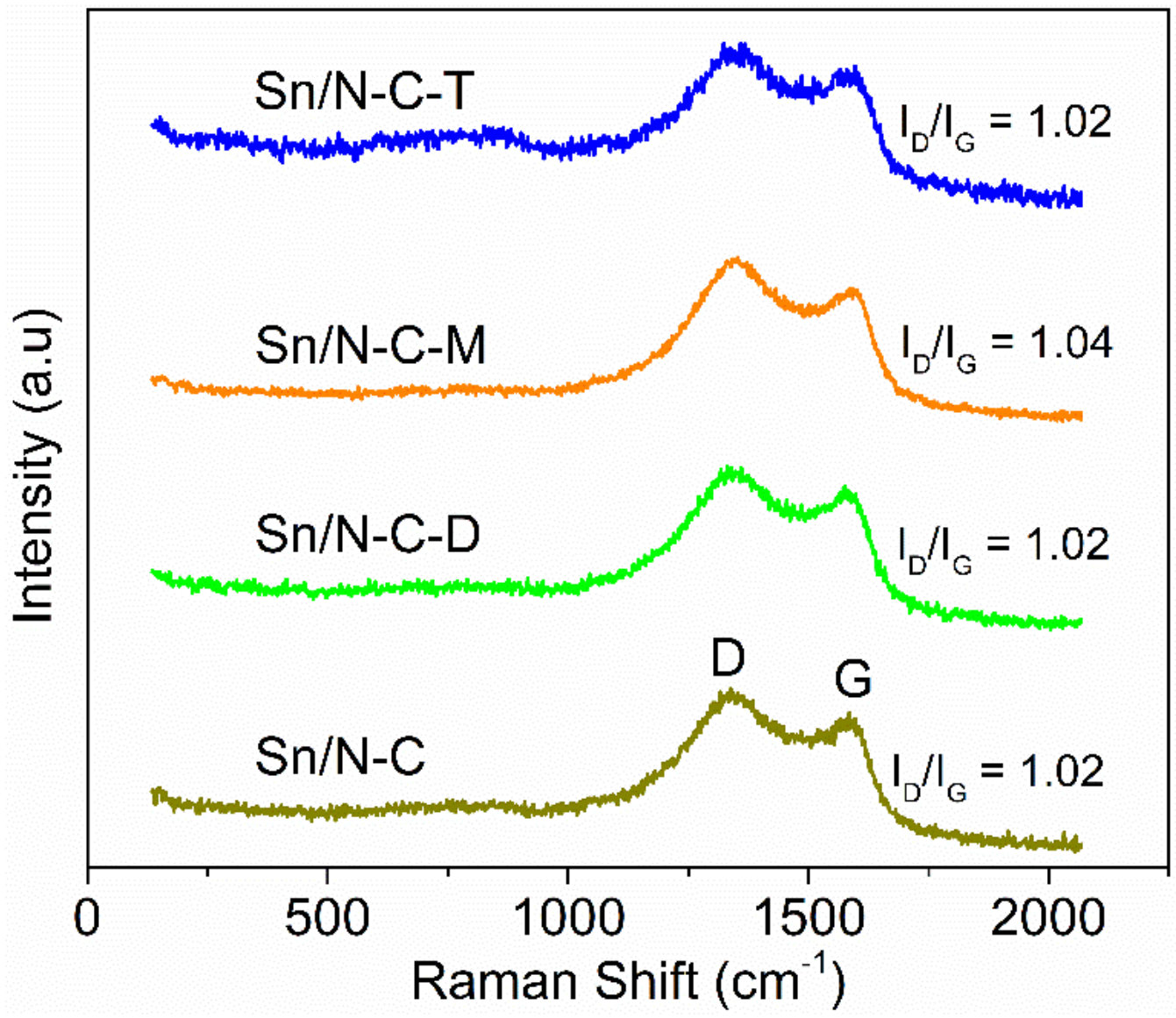

Figure S13. Raman spectra of **Sn/N-C**, **Sn/N-C-D**, **Sn/N-C-M**, and **Sn/N-C-T** catalysts.

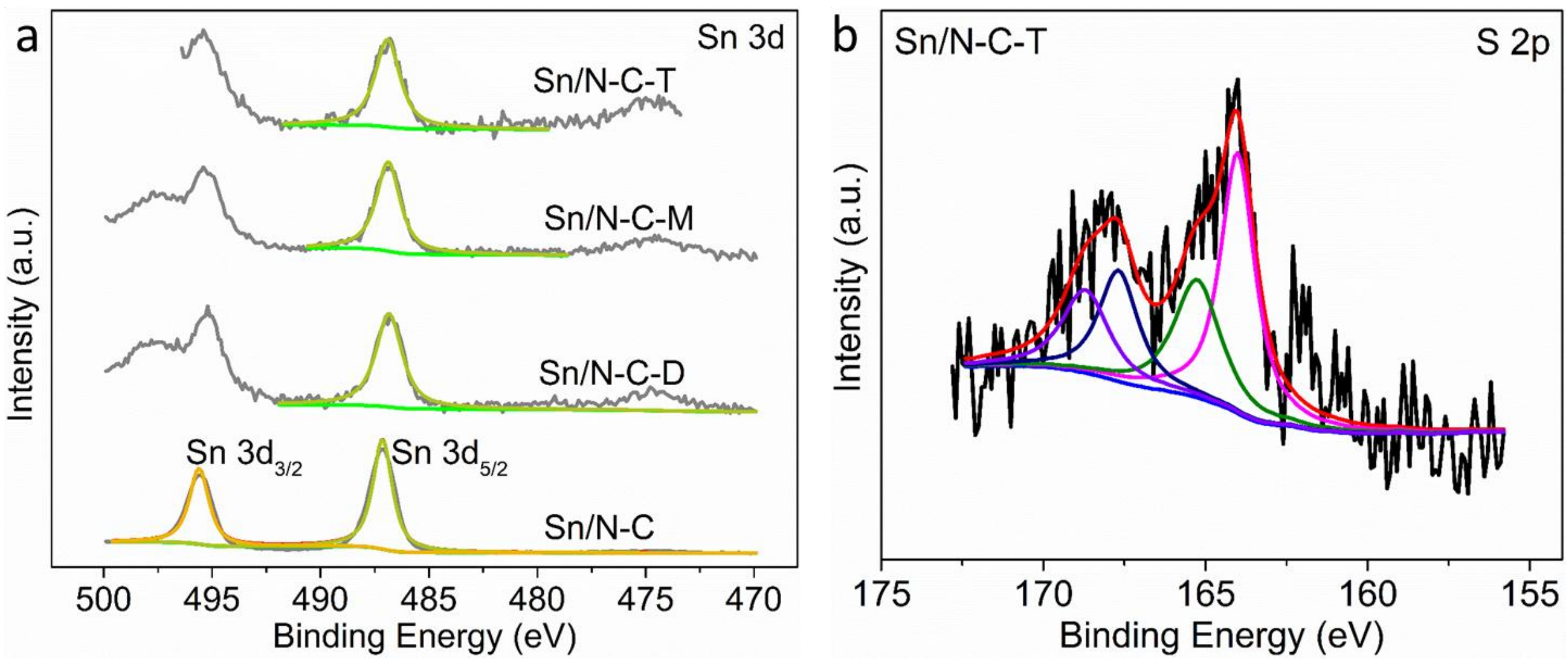

Figure S14. Deconvoluted XPS spectra: (a) Sn 3d XPS spectra for **Sn/N-C**, **Sn/N-C-D**, **Sn/N-C-M**, and **Sn/N-C-T** catalysts. (b) S 2p XPS spectra for **Sn/N-C-T** catalyst.

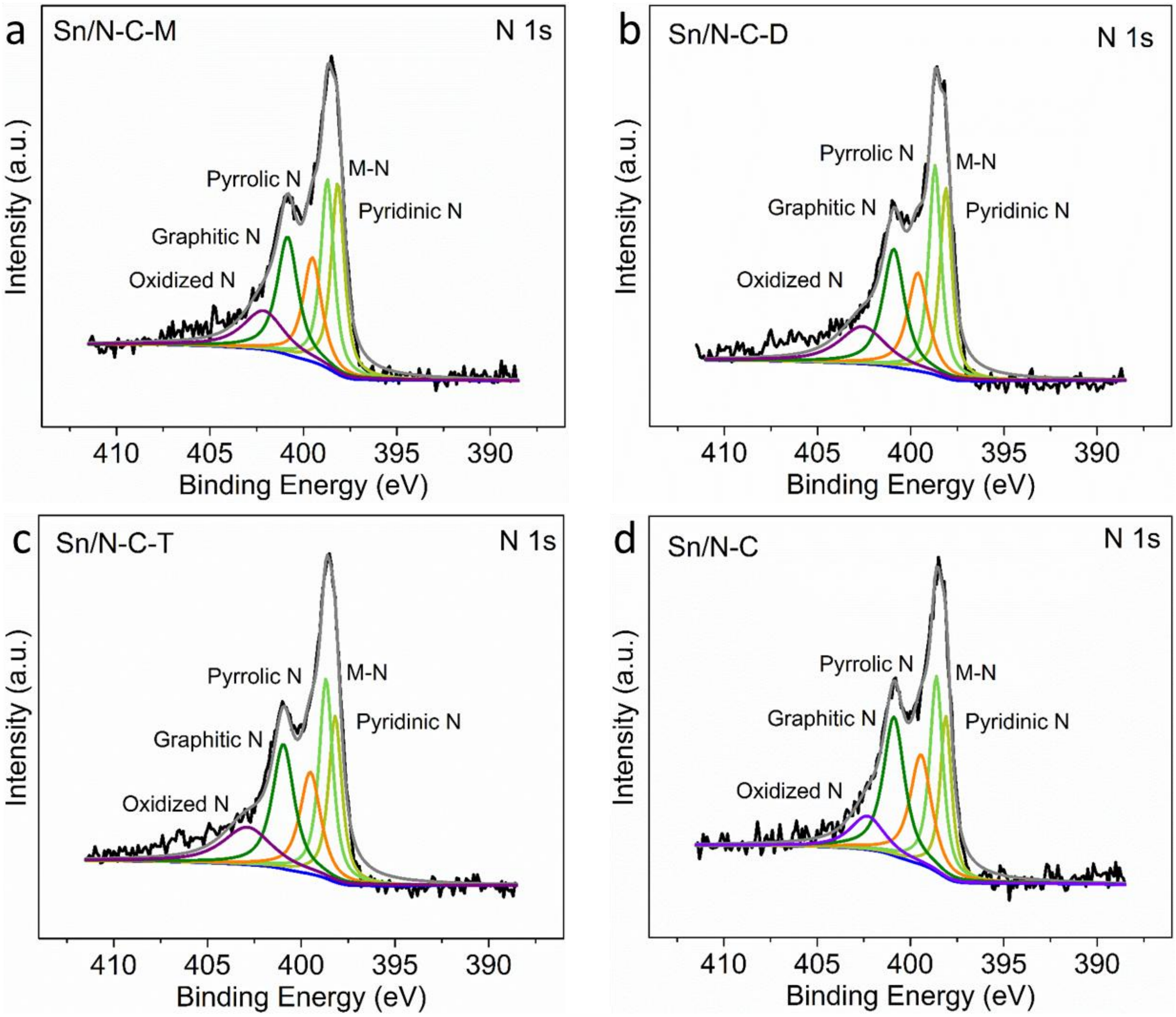

Figure S15. Deconvoluted XPS spectra: N 1s XPS spectra for (a) **Sn/N-C-M**, (b) **Sn/N-C-D**, (c) **Sn/N-C-T**, and (d) **Sn/N-C** catalysts.

Table S1. Quantitative analysis of N species in Sn/N-C-M, Sn/N-C-D, Sn/N-C-T, and Sn/N-C catalysts obtained from N 1s XPS peak deconvolution.

Sample	Pyridinic N (%)	Pyrrolic N (%)	Graphitic N (%)	Oxidized N (%)	Sn-N associated (%)
Sn/N-C-M	24.3	19.9	18.1	14.8	22.9
Sn/N-C-D	22.2	19.7	20.4	16.1	21.6
Sn/N-C-T	21.9	19.9	20.6	16.8	20.8
Sn/N-C	20.1	19.4	22.3	16.3	21.9

The N 1s spectra were fitted using a Shirley background and Gaussian–Lorentzian peak shapes. The binding energy positions and full width at half maximum (FWHM) values were constrained within reasonable ranges and identical fitting criteria were applied to all samples. The relative contents of pyridinic N, pyrrolic N, graphitic N, oxidized N, and Sn-N associated

values were calculated from the integrated areas of the corresponding fitted components after background subtraction. The same fitting procedure, peak constraints, and line-shape parameters were applied to all samples

Supplementary Note

In the initial step, ZIF-8 was enriched with a Zn content of approximately 19.22 wt.%, as determined by ICP-OES (Table S2). After pyrolysis, the resulting nitrogen-doped carbon matrix (N-C) retained a Zn content of about 8.02 wt.%, indicating partial removal of Zn during high-temperature treatment. However, a significant reduction in Zn content to ~1.03 wt.% was achieved after acid leaching under aggressive reflux conditions. This demonstrates that while pyrolysis can remove loosely bound Zn species, the coordinated Zn sites are thermally stable and largely retained. In contrast, aggressive acid leaching is far more effective in eliminating residual Zn, highlighting its critical role in purifying the N-C matrix.

Table S2. Elemental composition of different catalysts determined by ICP-OES

Samples	Sn (wt.%)	Zn (wt.%)
Sn/N-C	8.20	0.56
Sn/N-C-M	7.51	0.54
Sn/N-C-D	7.49	0.51
Sn/N-C-T	7.71	0.53
N-C after acid leaching	-	1.03
N-C before acid leaching	-	8.02
ZIF-8	-	19.22

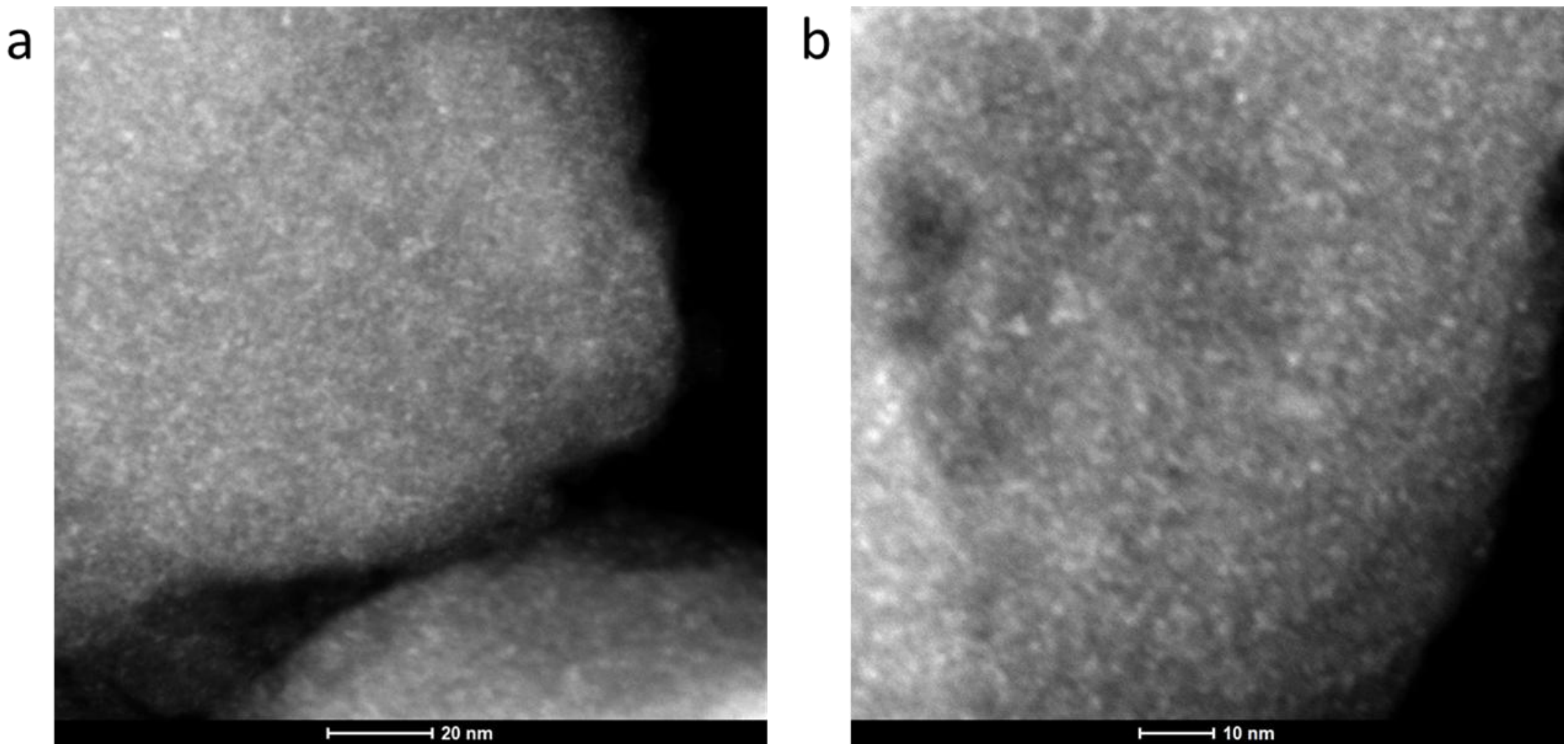

Figure S16. Aberration-corrected HAADF-STEM images of **Sn/N-C-D** catalyst.

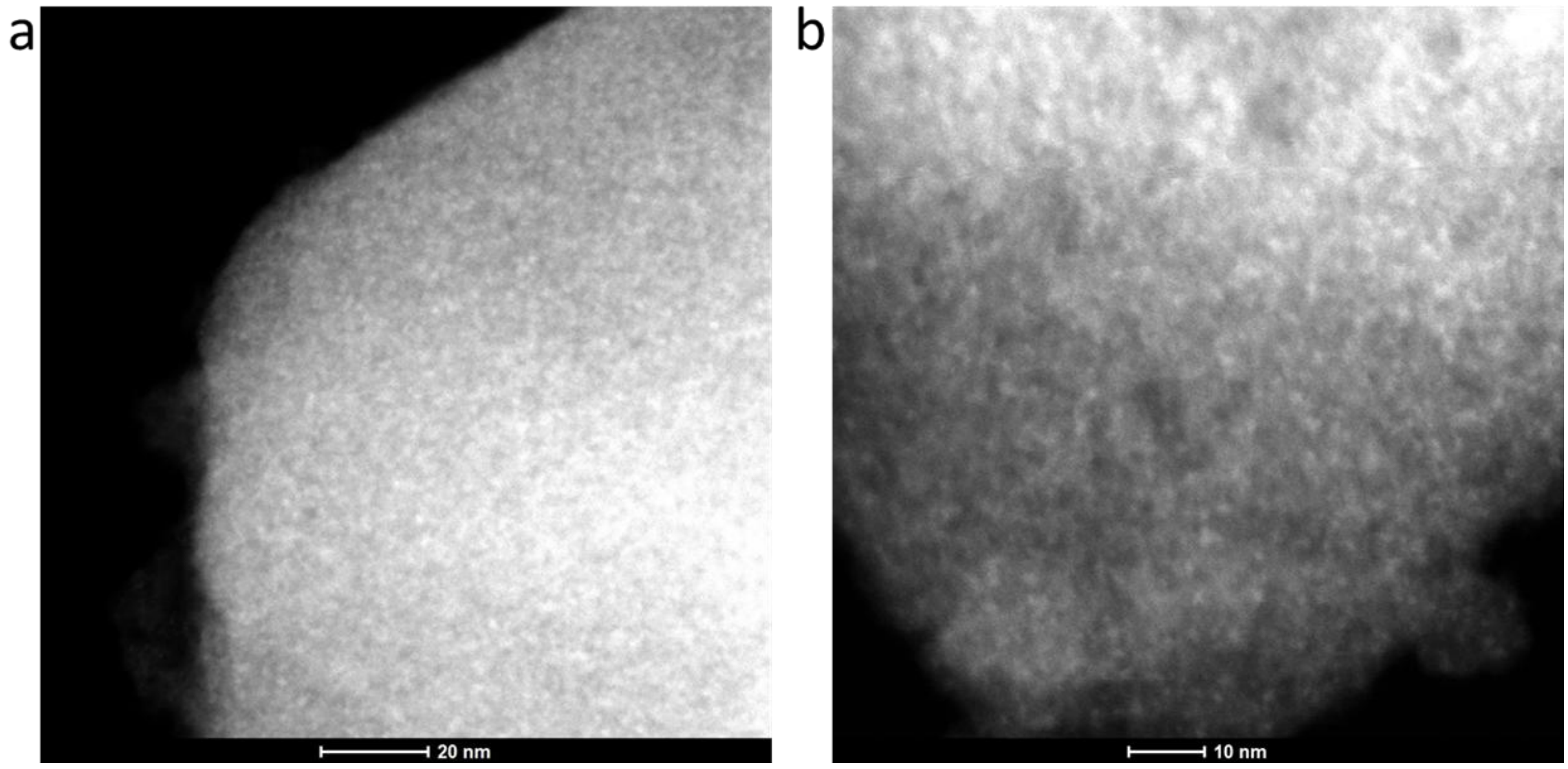

Figure S17. Aberration-corrected HAADF-STEM images of **Sn/N-C-T** catalyst.

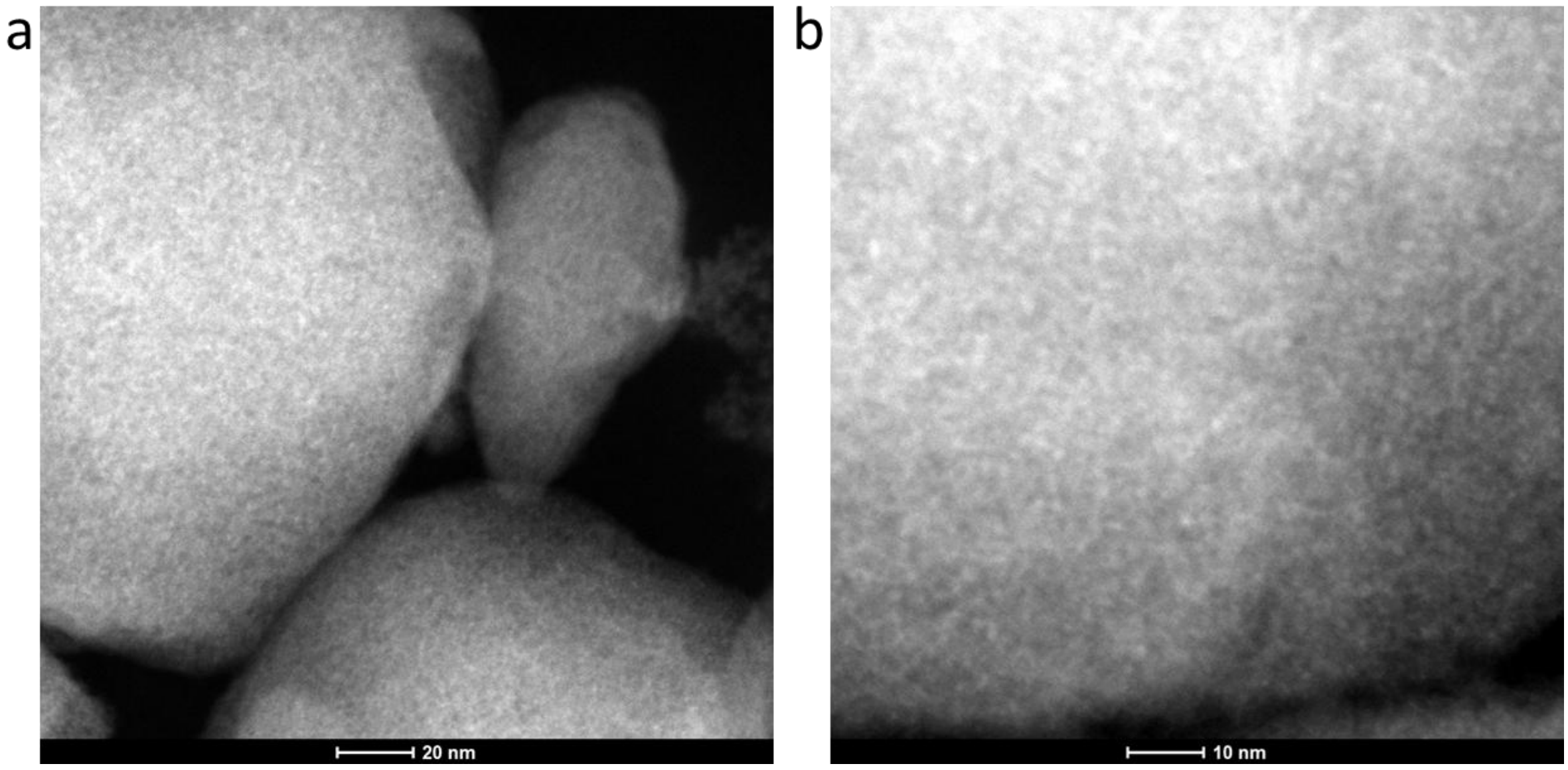

Figure S18. Aberration-corrected HAADF-STEM images of **Sn/N-C** catalyst.

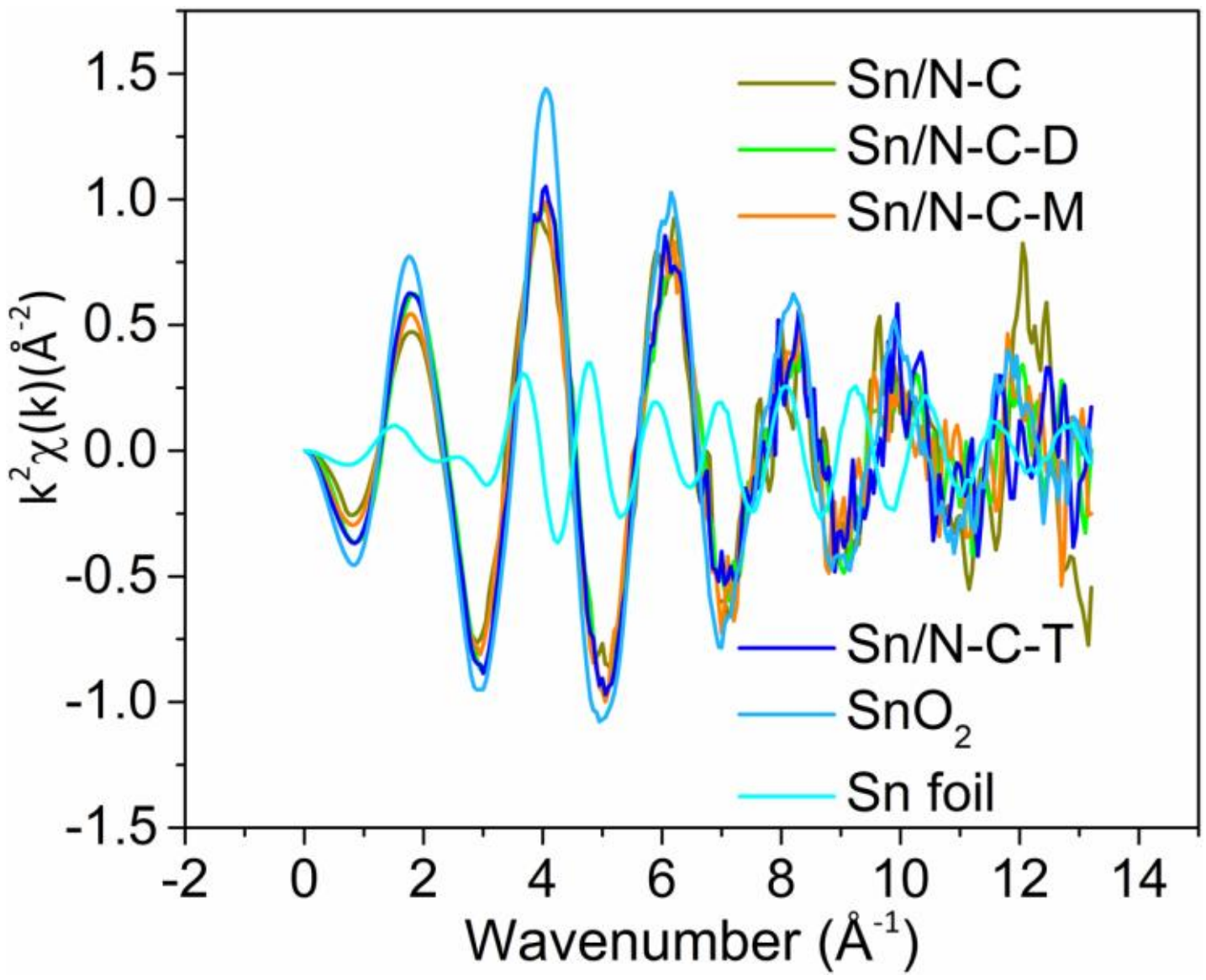

Figure S19. The XANES spectra of extended oscillation functions $k^2\chi(k)$ for Sn K-edge of **Sn/N-C**, **Sn/N-C-D**, **Sn/N-C-M**, and **Sn/N-C-T** catalysts.

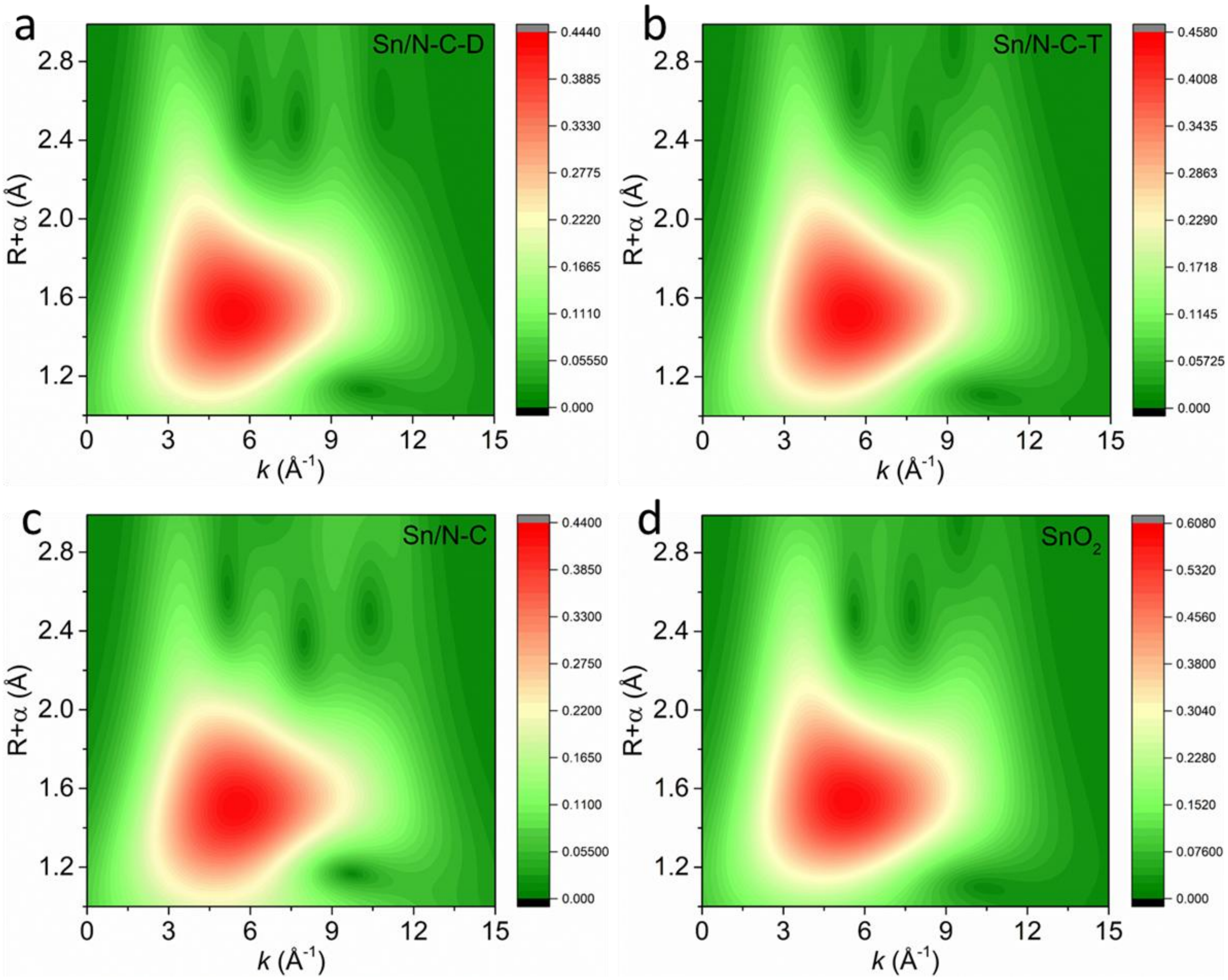

Figure S20. Wavelet transform (WT) EXAFS of the k^3-weighted *k*-space for (a) **Sn/N-C-D**, (b) S**n/N-C-T**, (c) **Sn/N-C** catalysts and (d) reference SnO_2.

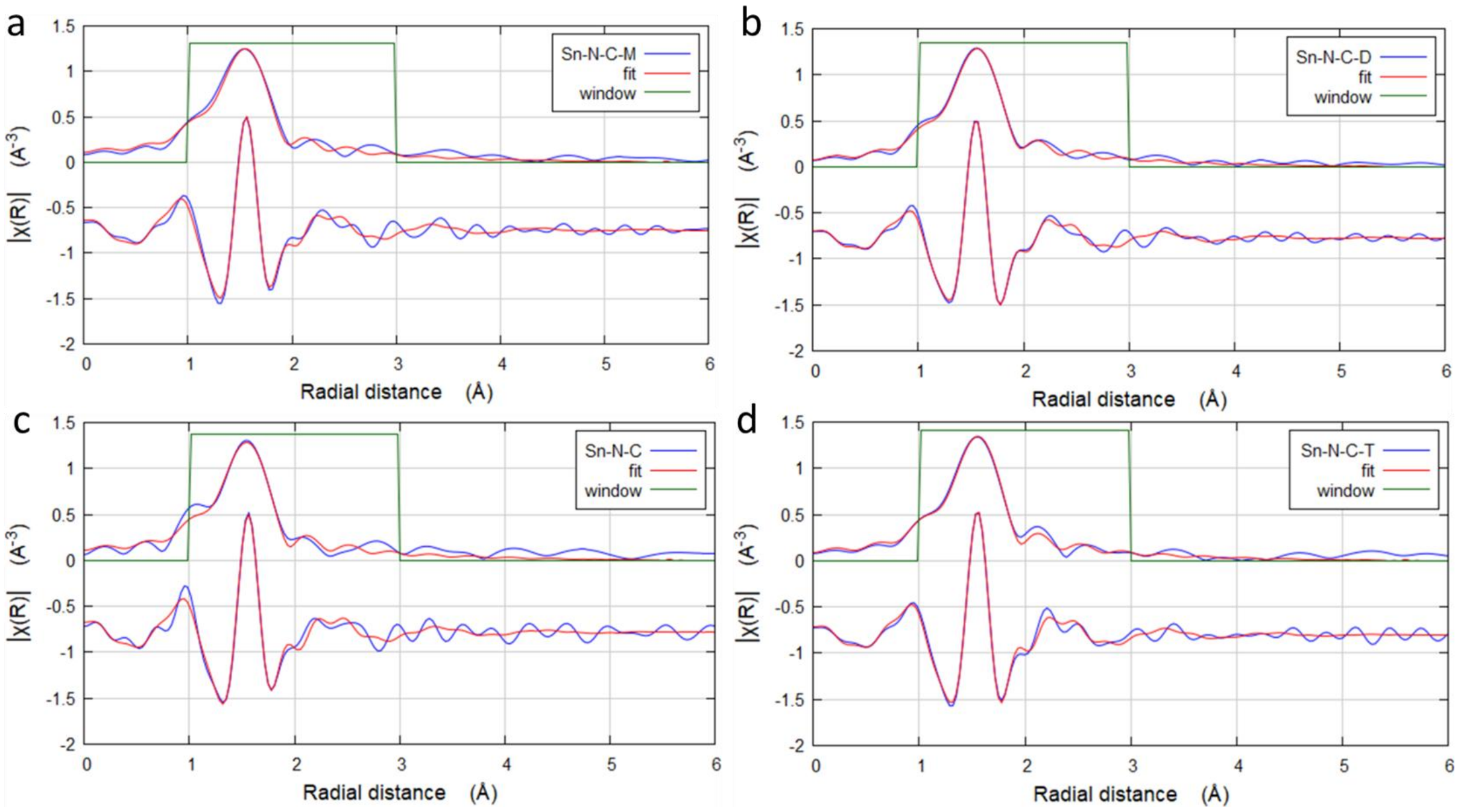

Figure S21. Fitting analysis of the Fourier-transformed magnitude of EXAFS spectra in R-space and k-space of Sn K-edge for (a) **Sn/N-C-M**, (b) **Sn/N-C-D**, (c) **Sn/N-C**, and (d) **Sn/N-C-T** samples.

Table S3. Local structure parameters of Sn-N for **Sn/N-C-M, Sn/N-C-D, Sn/N-C** and Sn-N/S for **Sn/N-C-T** derived from EXAFS analysis; R: bond distance in R-space; E_0: edge energy shift; σ^2: Debye Waller factor.

Sample	Scattering Path	Coordination Number	R (Å)	ΔE_0 (eV)	σ^2 (Å^2)	R-factor
**Sn/N-C-M**	Sn-N	4.0 ± 0.4	2.05 ± 0.01	4.3	0.005 ± 0.002	0.005
	Sn-Sn	-	-			
**Sn/N-C-D**	Sn-N	4.0 ± 0.1	2.05 ± 0.01	6.1	0.004 ± 0.002	0.005
	Sn-Sn	-	-			
**Sn/N-C**	Sn-N	3.9 ± 0.5	2.05 ± 0.02	3.4	0.004 ± 0.003	0.005
	Sn-Sn	-	-			
**Sn/N-C-T**	Sn-N	3.6 ± 0.3	2.05 ± 0.01	5.3	0.003 ± 0.002	0.005
	Sn-S	0.7 ± 0.2	2.25 ± 0.01		0.003 ± 0.002	
	Sn-Sn	-	-			

EXAFS fitting procedure. The first coordination shell of the Sn K-edge EXAFS spectra was evaluated using several plausible structural models, including Sn-N, Sn-O, and Sn-C coordination environments, as well as mixed coordination models where appropriate. The fitting was performed over the same *k*- and *R*-ranges using identical fitting parameters and constraints to enable direct comparison between models. Among the models considered, the Sn-N coordination model consistently provided the best agreement with the experimental spectra, as evidenced by the lowest fitting residuals (R-factor) and physically reasonable coordination numbers and bond distances. We note that EXAFS alone cannot unambiguously distinguish between N, O, and C first-shell scatterers because of their similar atomic numbers and backscattering amplitudes. Therefore, the assignment of predominantly Sn-N coordination is based on the superior fitting quality of the Sn-N model together with complementary characterization, including the nitrogen-rich synthesis, XPS analysis, HAADF-STEM observations, and the absence of Sn-Sn scattering in both EXAFS and WT-EXAFS analyses.

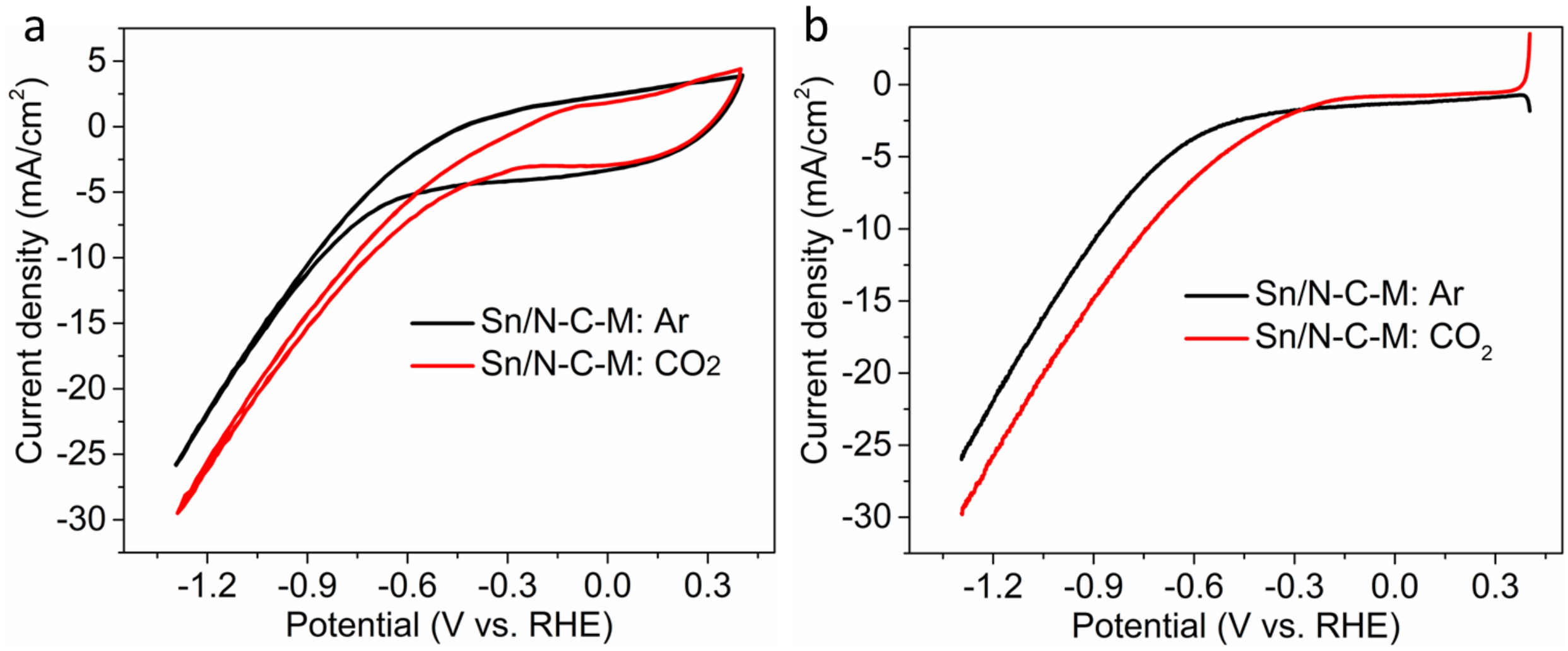

Figure S22. (a) CV (b) LSV curves over of **Sn/N-C-M** catalyst in Ar- and CO_2-saturated 0.1 M $KHCO_3$ electrolyte. (Scan rate: 50 mV s^{-1} for CV and 5 mV s^{-1} for LSV).

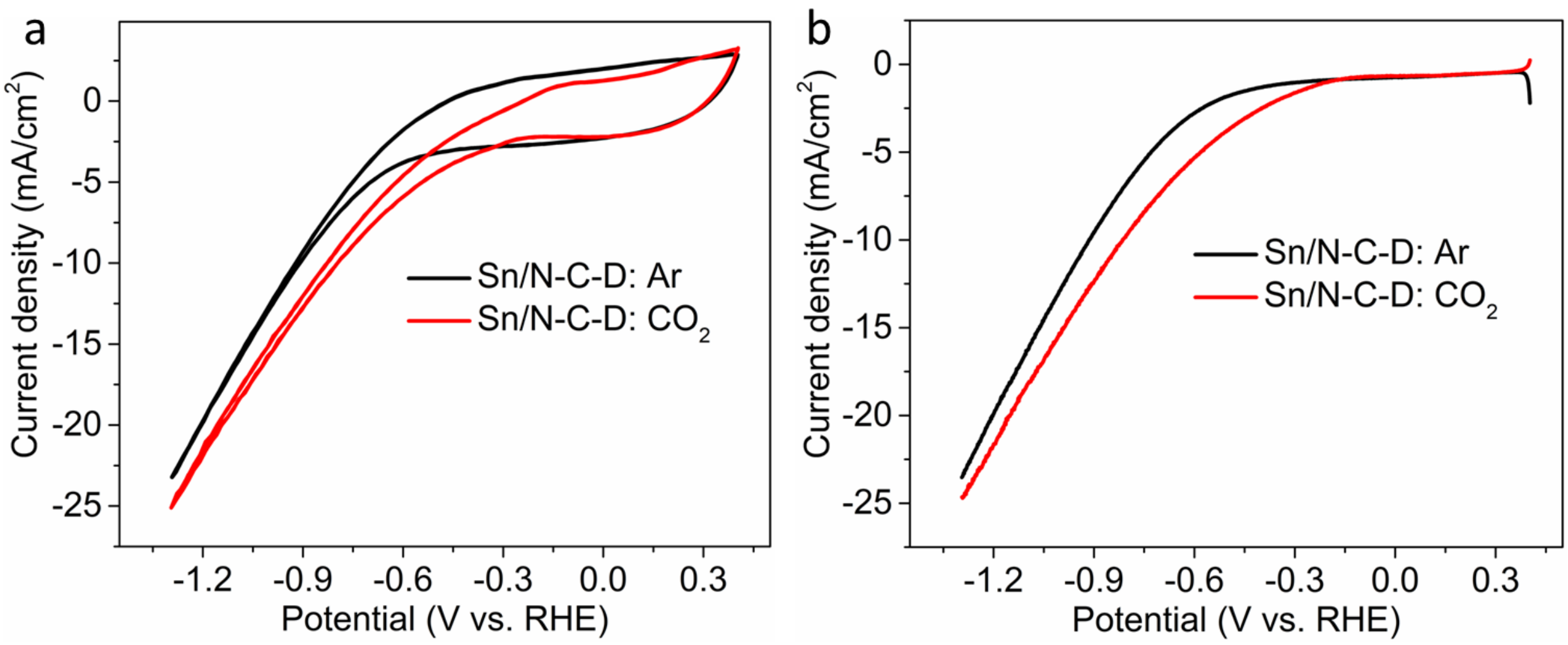

Figure S23. (a) CV (b) LSV curves over **Sn/N-C-D** catalyst in Ar- and CO_2-saturated 0.1 M $KHCO_3$ electrolyte. (Scan rate: 50 mV s^{-1} for CV and 5 mV s^{-1} for LSV).

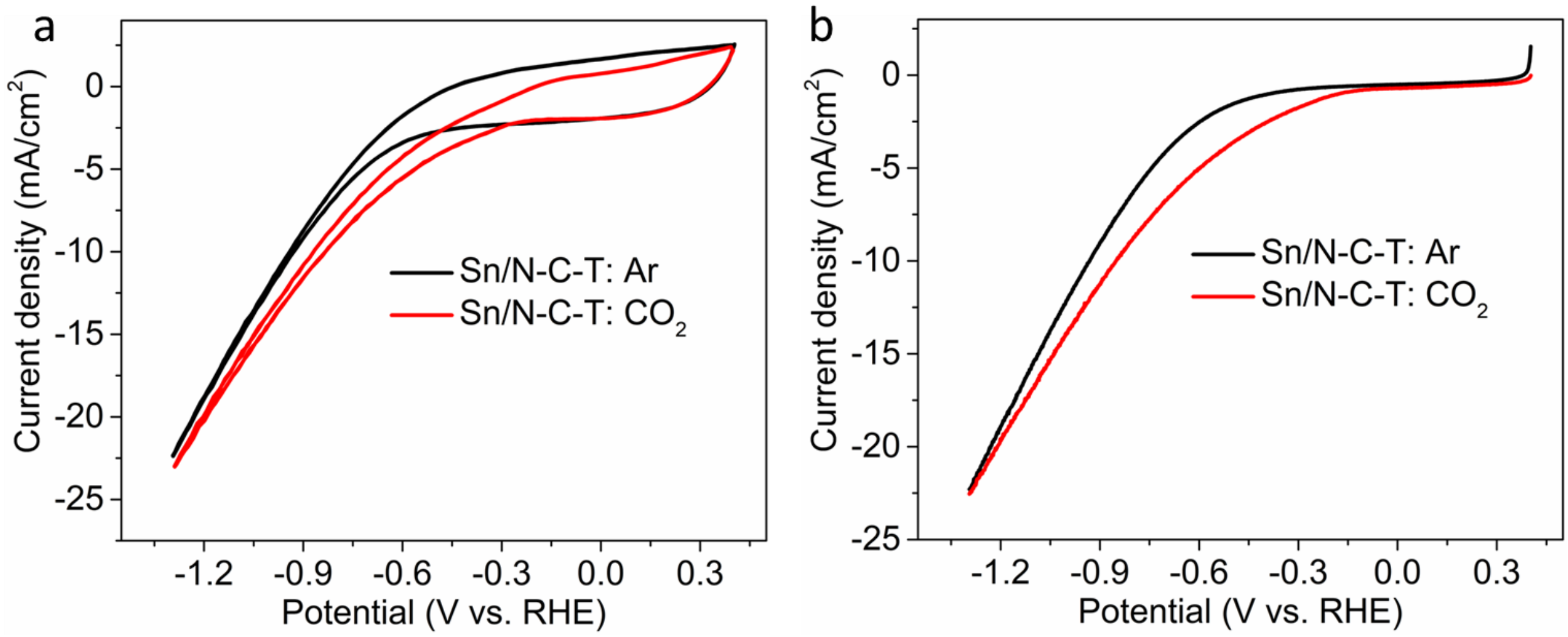

Figure S24. (a) CV (b) LSV curves over **Sn/N-C-T** catalyst in Ar- and CO_2-saturated 0.1 M $KHCO_3$ electrolyte. (Scan rate: 50 mV s^{-1} for CV and 5 mV s^{-1} for LSV).

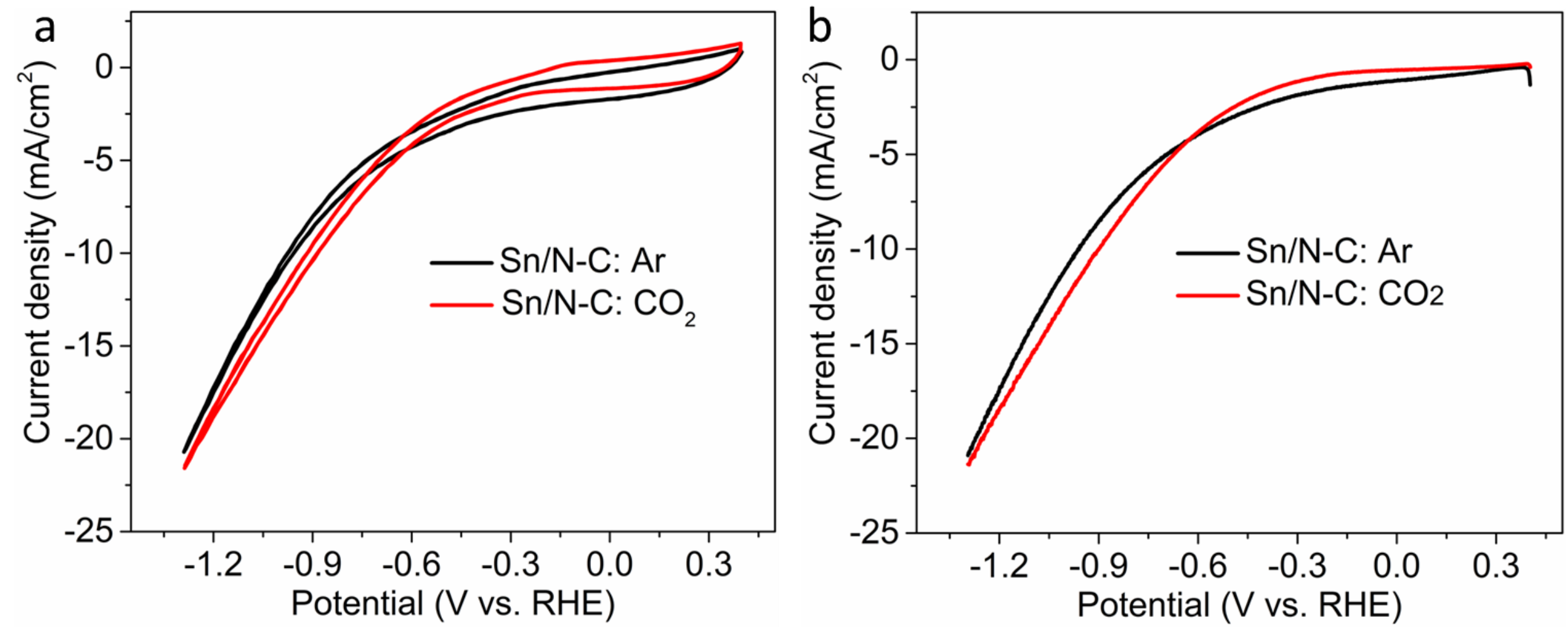

Figure S25. (a) CV (b) LSV curves over **Sn/N-C** catalyst in Ar- and CO_2-saturated 0.1 M $KHCO_3$ electrolyte. (Scan rate: 50 mV s^{-1} for CV and 5 mV s^{-1} for LSV).

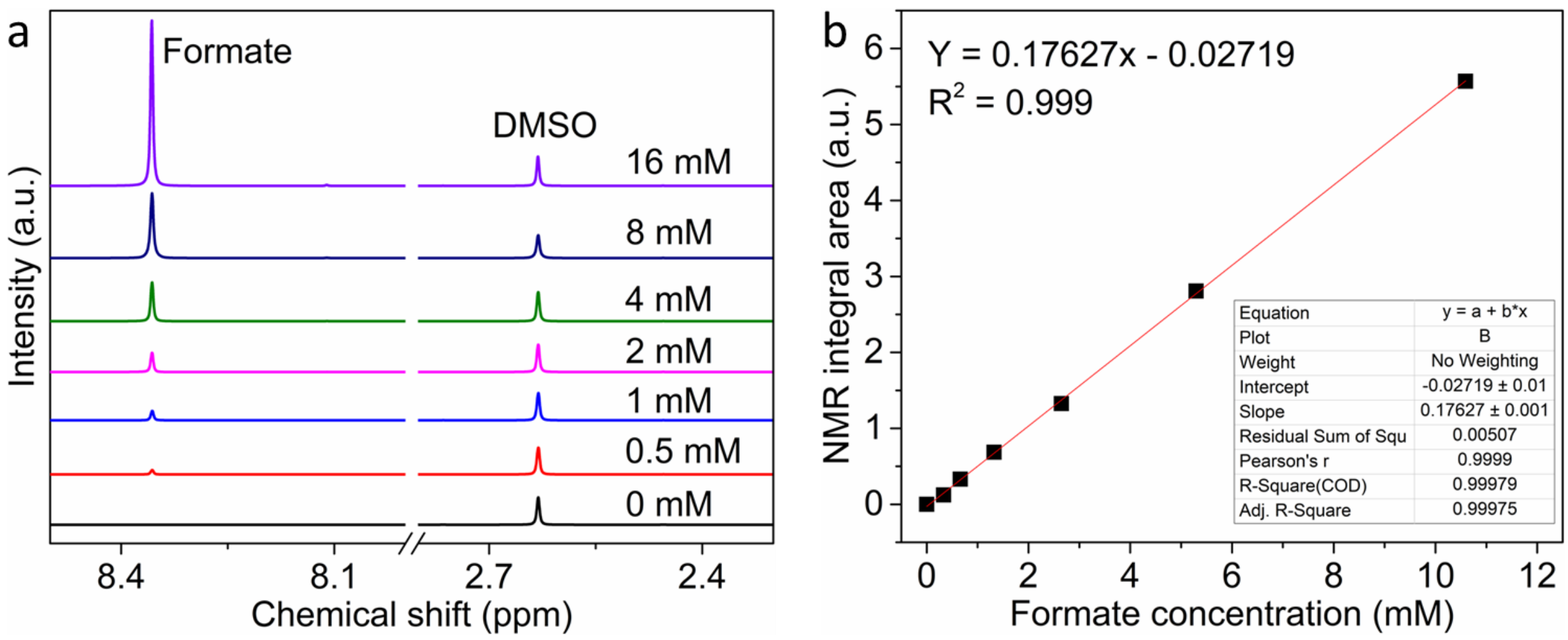

Figure S26. ^{1}H-NMR spectrum of the standard formate samples and calibration curves based on the area of the formate peak in the NMR spectra.

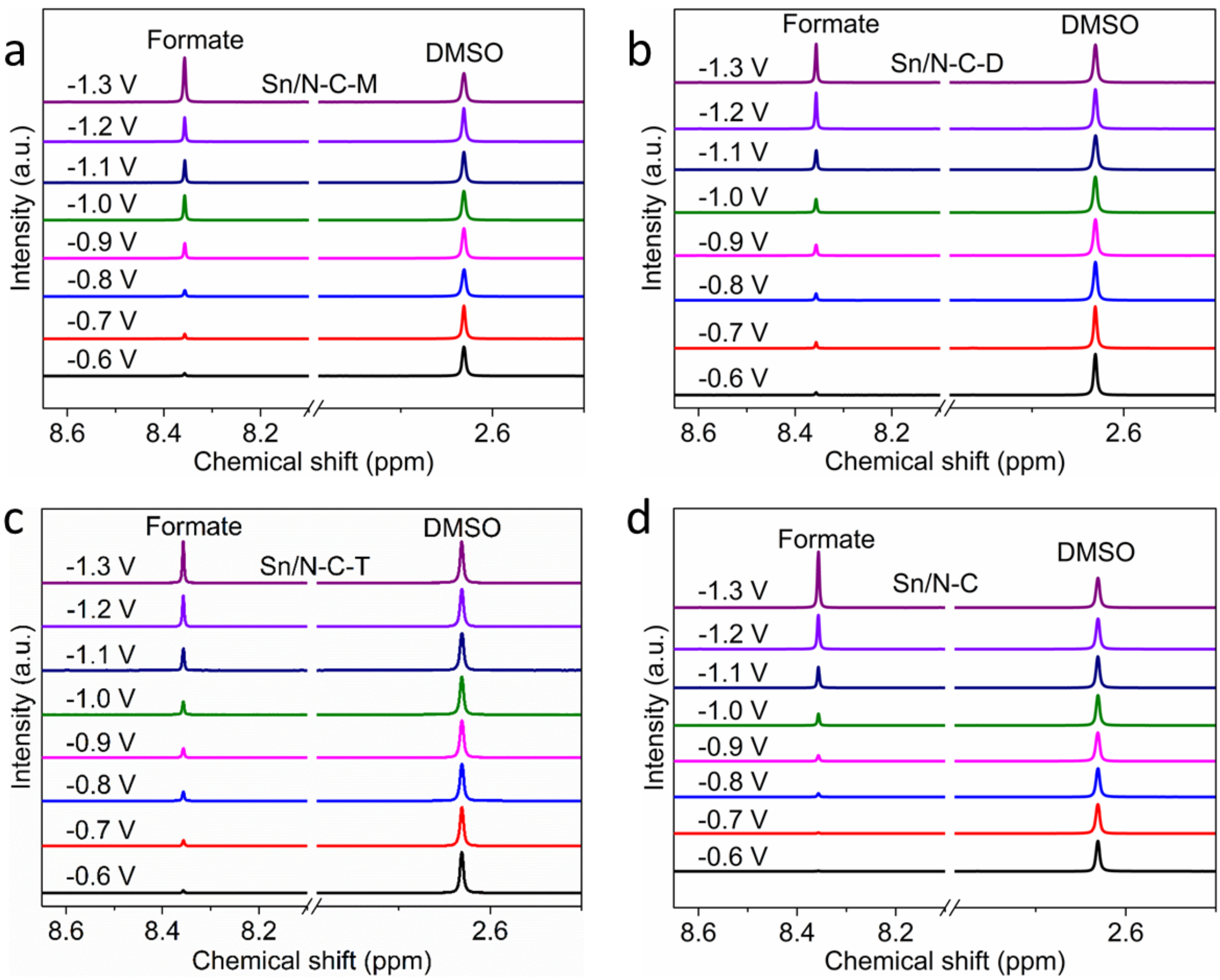

Figure S27. Representative ^{1}H-NMR spectrum of liquid products collected from the cathode side electrolyte after 3 h electrocatalysis at different potentials versus RHE over (a) **Sn/N-C-M,** (b) **Sn/N-C-D,** (c) **Sn/N-C-T,** and (d) **Sn/N-C** catalysts.

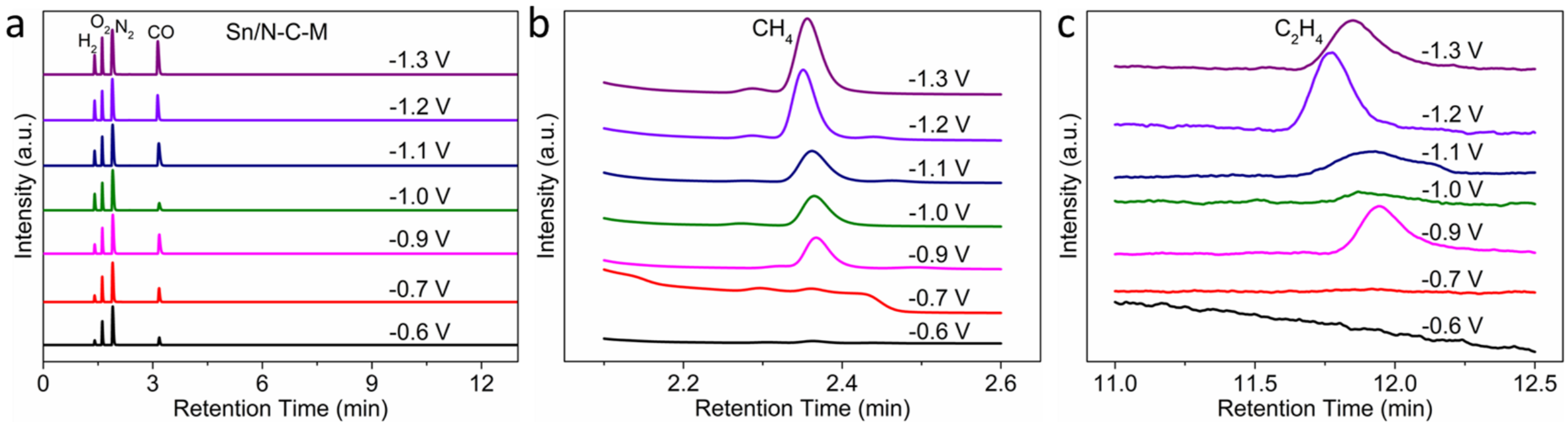

Figure S28. Representative GC spectra of gas products collected from the cathode headspace after 3 h electrocatalysis at different potentials versus RHE over **Sn/N-C-M** catalyst.

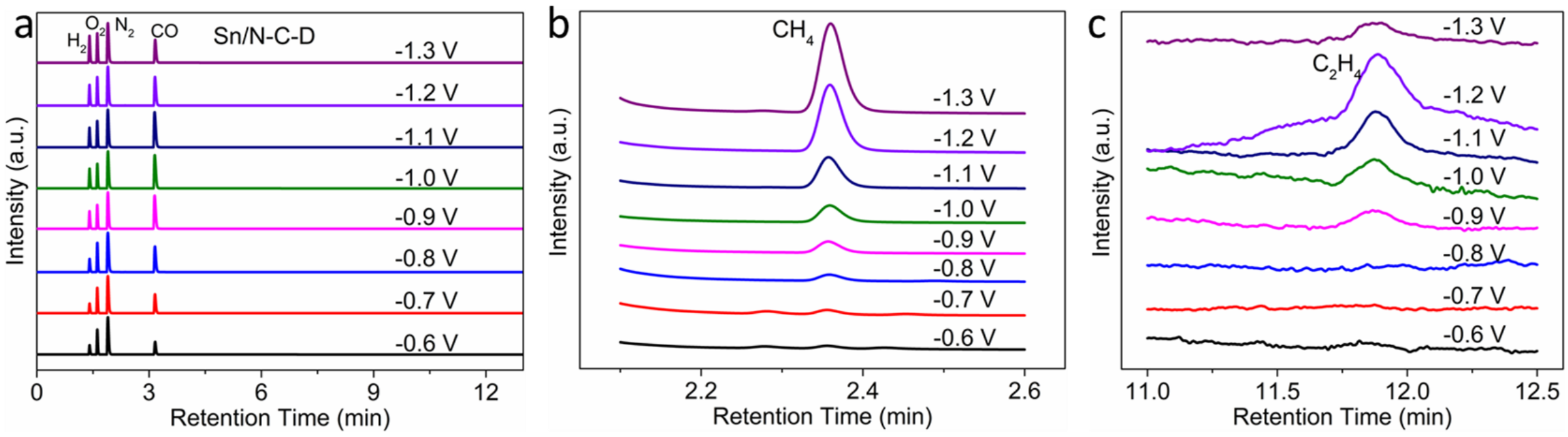

Figure S29. Representative GC spectra of gas products collected from the cathode headspace after 3 h electrocatalysis at different potentials versus RHE over **Sn/N-C-D** catalyst.

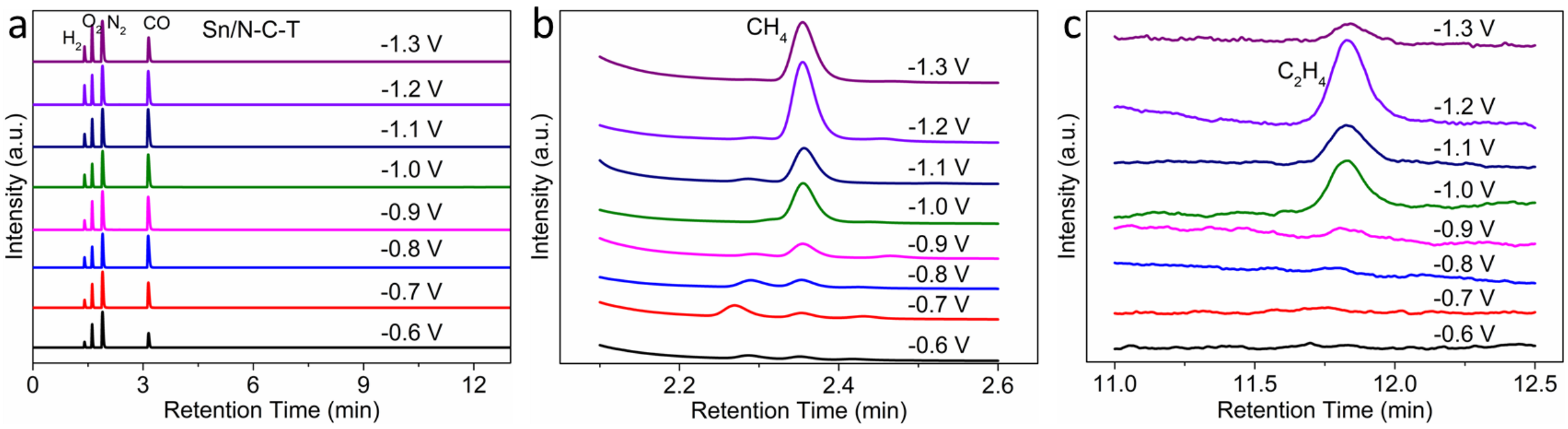

Figure S30. Representative GC spectra of gas products collected from the cathode headspace after 3 h electrocatalysis at different potentials versus RHE over **Sn/N-C-T** catalyst.

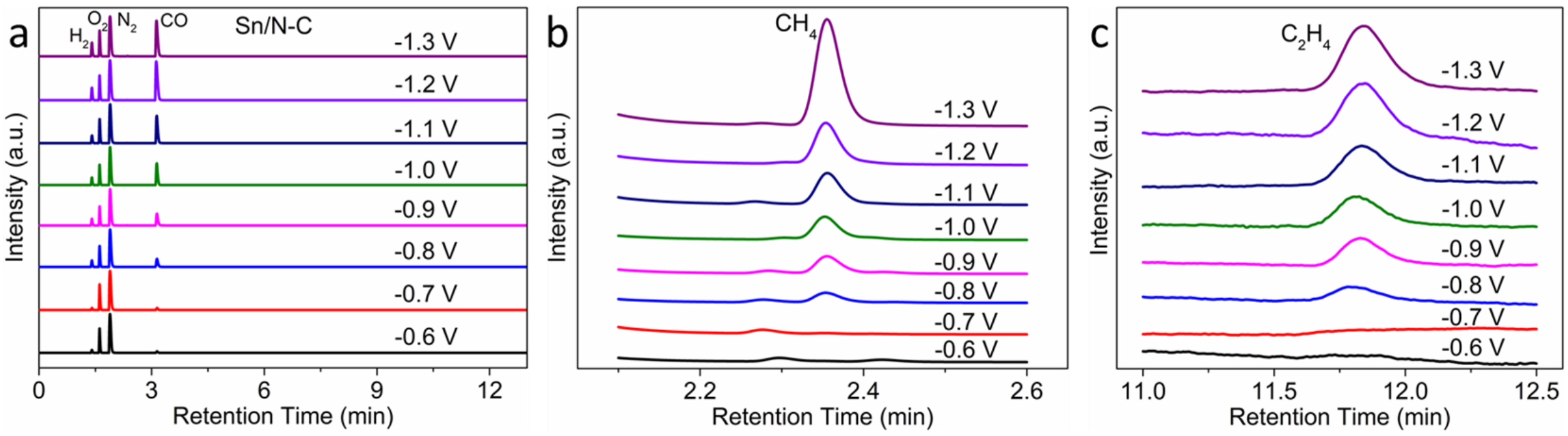

Figure S31. Representative GC spectra of gas products collected from the cathode headspace after 3 h electrocatalysis at different potentials versus RHE over **Sn/N-C** catalyst.

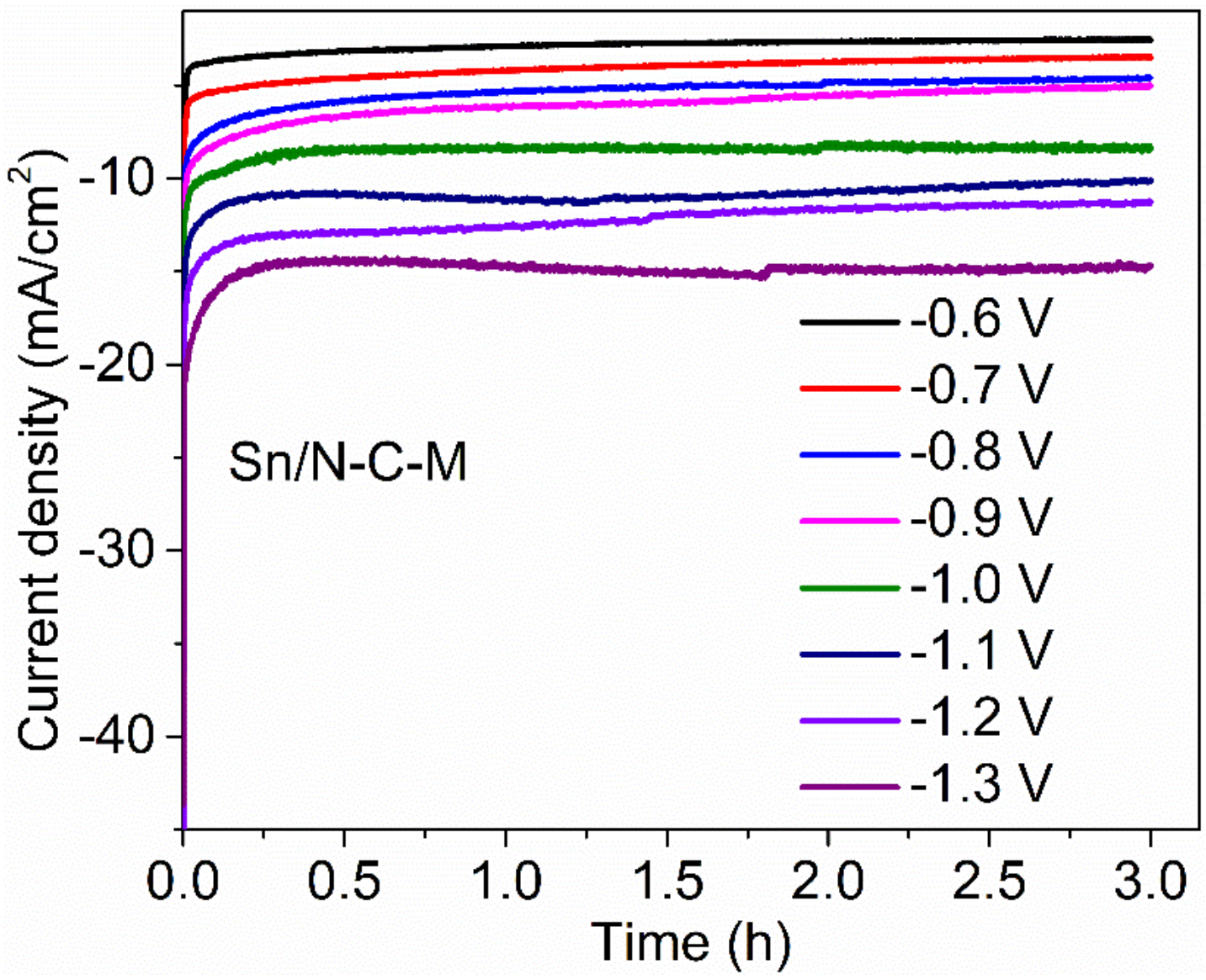

Figure S32. (a) Chronoamperometry measurements at different applied potentials over **Sn/N-C-M** catalyst.

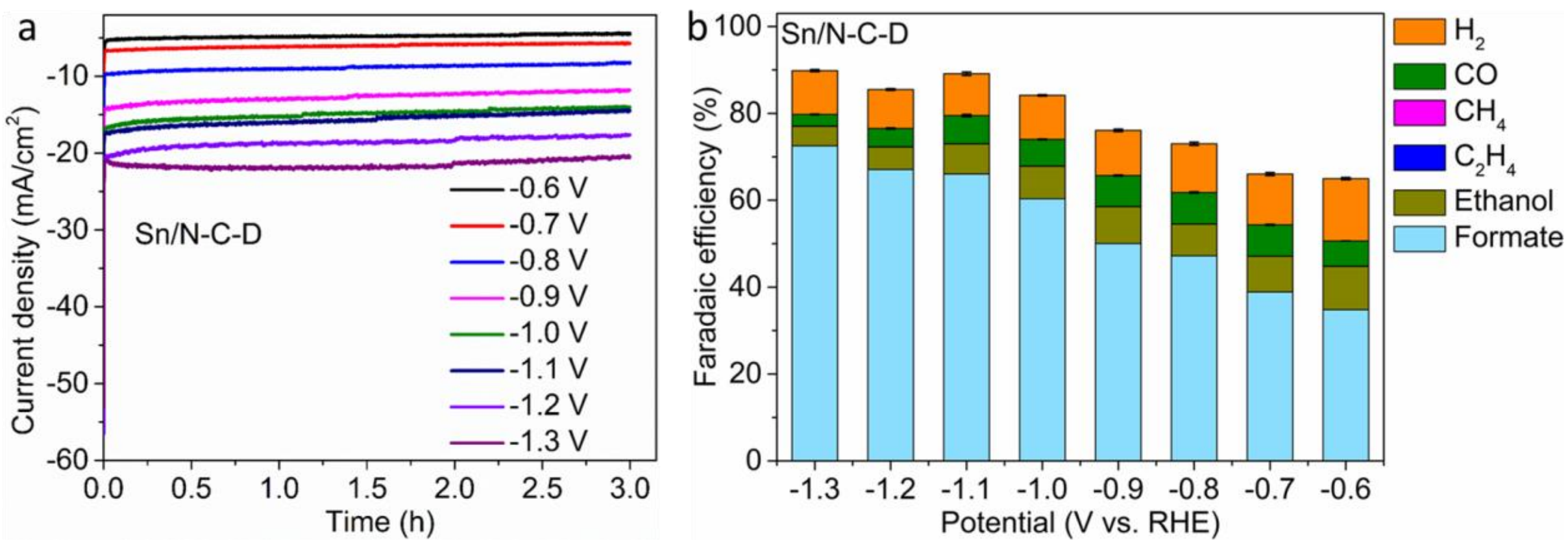

Figure S33. (a) Chronoamperometry measurements at different applied potentials for **Sn/N-C-D** catalyst. (b) FE and product distribution of electroreduction of CO_2 over **Sn/N-C-D** catalyst at different applied polarization potentials.

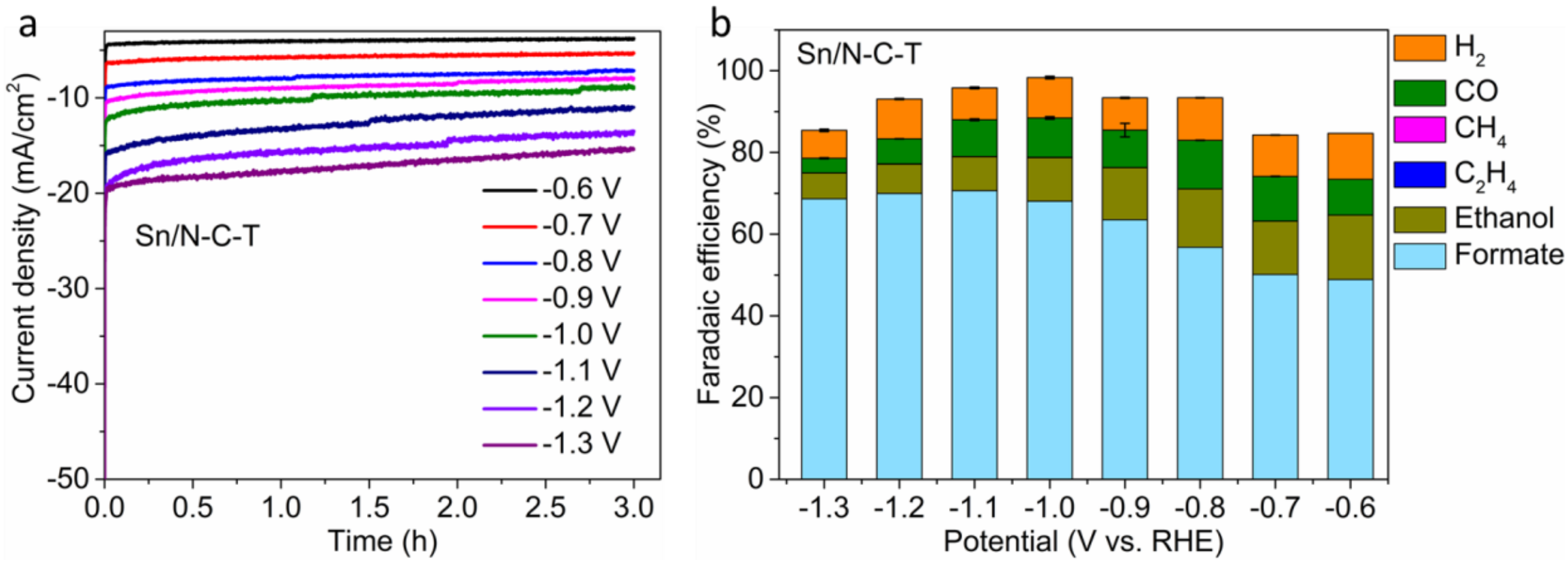

Figure S34. (a) Chronoamperometry measurements at different applied potentials for **Sn/N-C-T** catalyst. (b) FE and product distribution of electroreduction of CO_2 over **Sn/N-C-T** catalyst at different applied potentials.

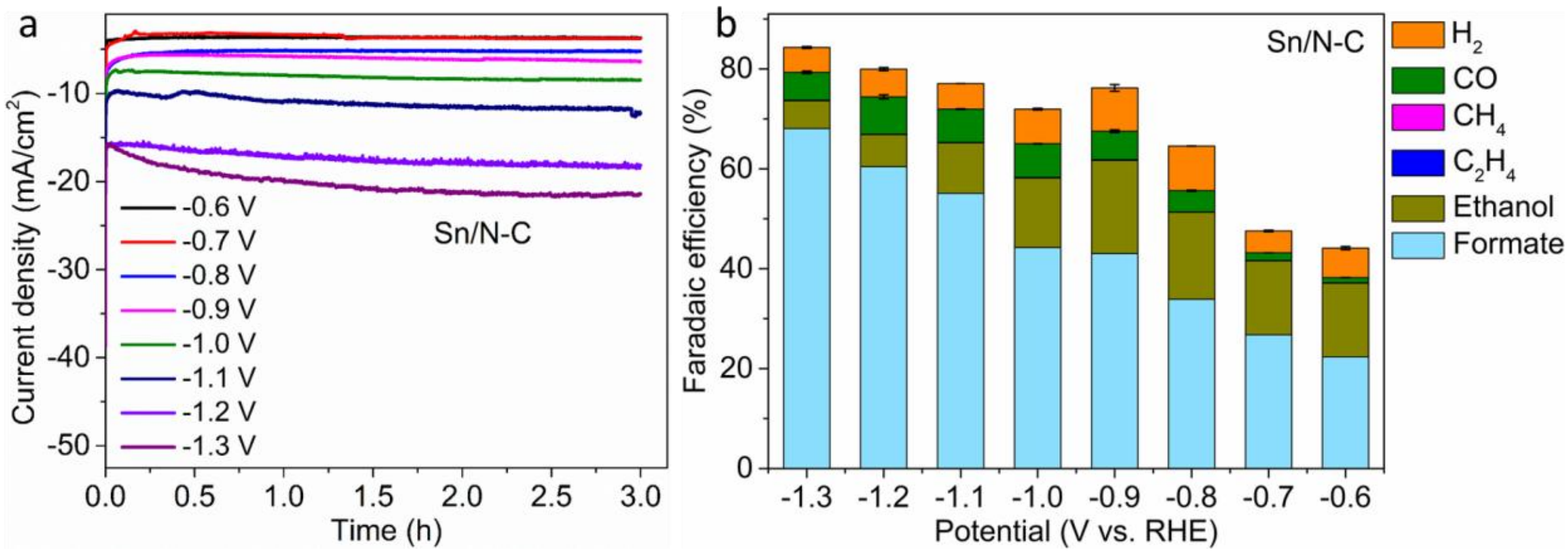

Figure S35. (a) Chronoamperometry measurements at different applied potentials for **Sn/N-C** catalyst. (b) FE and product distribution of electroreduction of CO_2 over **Sn/N-C** catalyst at different applied potentials.

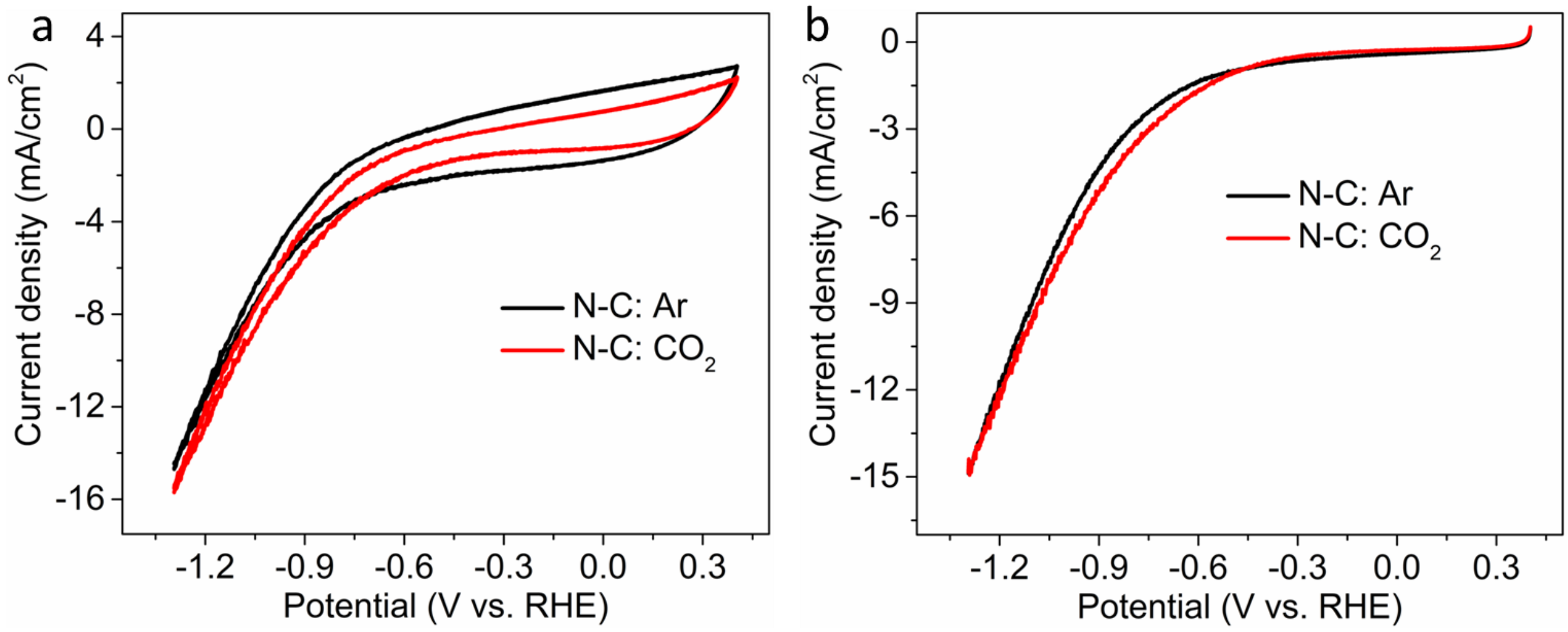

Figure S36. (a) CV (b) LSV curves over **N-C** support in Ar- and CO_2-saturated 0.1 M $KHCO_3$ electrolyte. (Scan rate: 50 mV s^{-1} for CV and 5 mV s^{-1} for LSV). The N-C used here is after acid leaching.

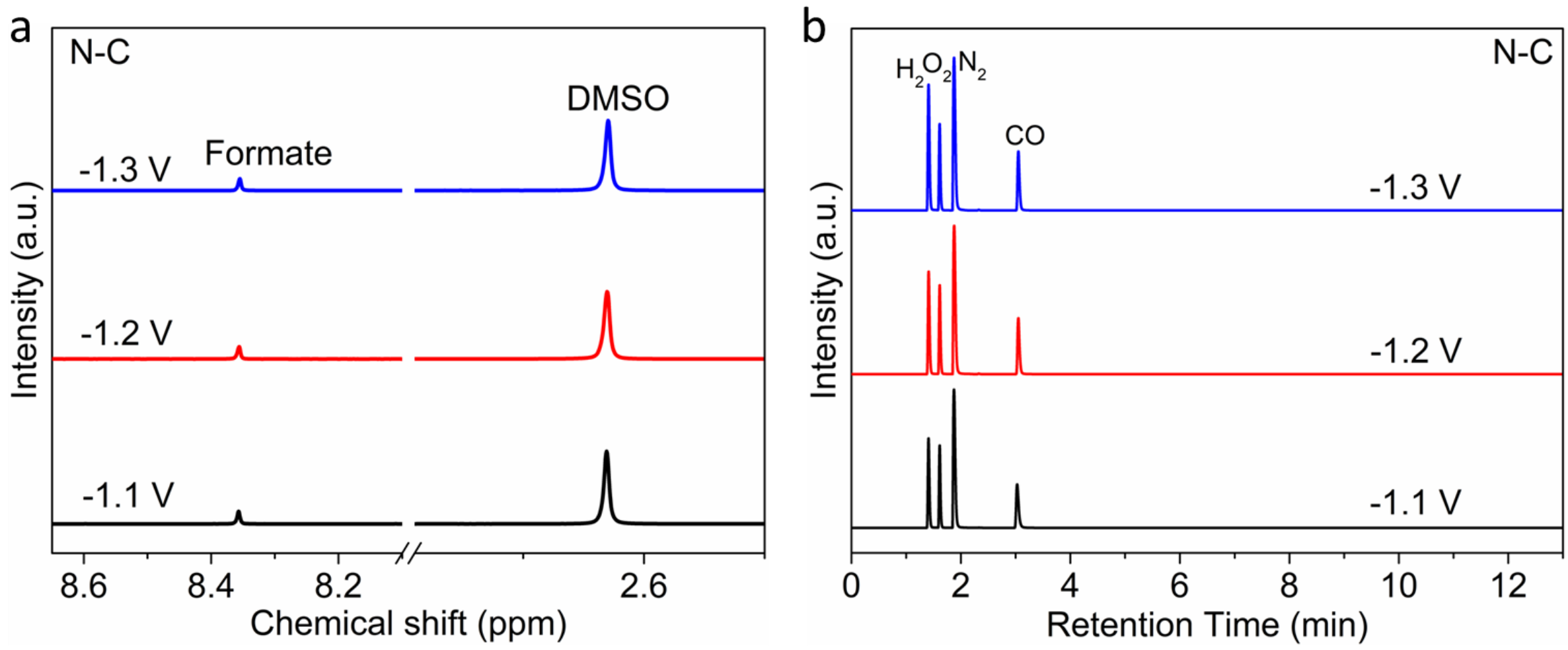

Figure S37. (a) ^{1}H-NMR spectrum of liquid products collected from the cathode side electrolyte after 3 h electrocatalysis at different potentials versus RHE over **N-C** support. (b) GC spectra of gas products collected from the cathode headspace after 3 h electrocatalysis at different potentials versus RHE over **N-C** support.

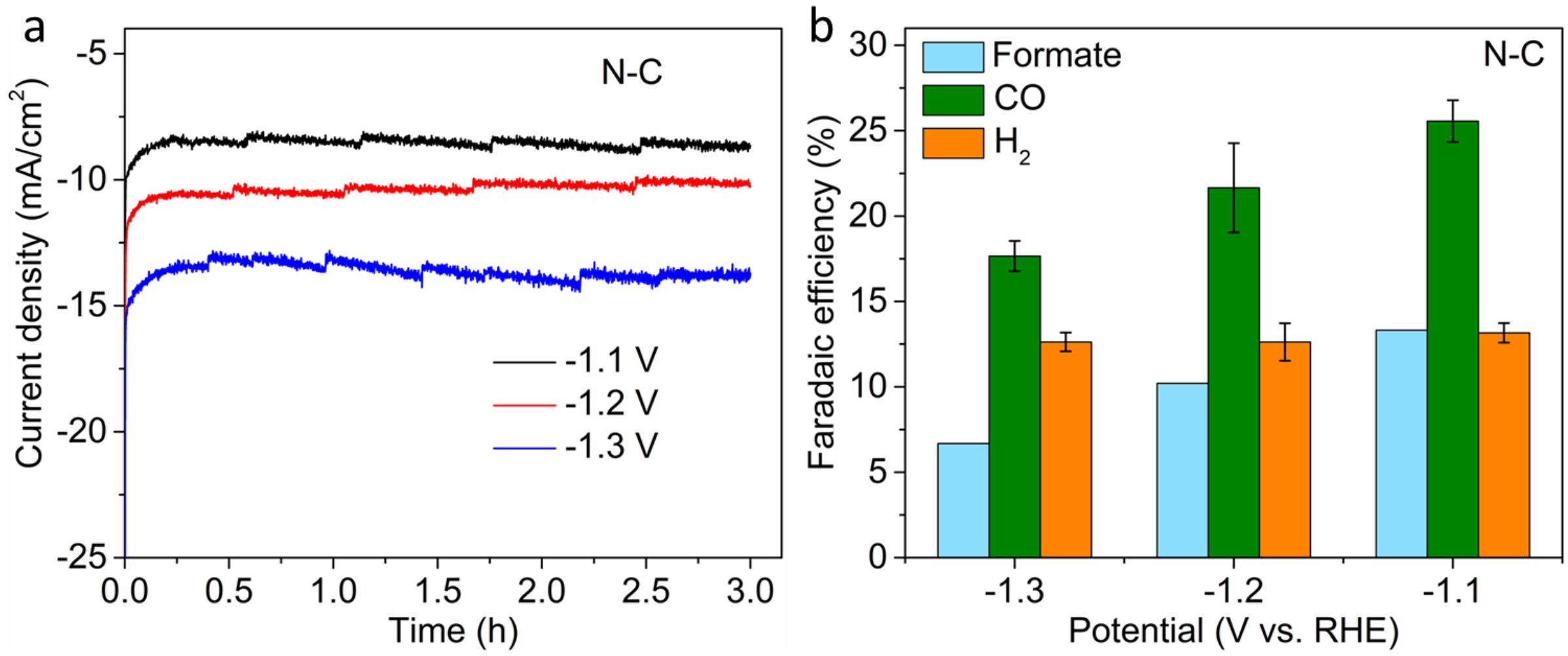

Figure S38. (a) Chronoamperometry measurements at different applied potentials for **N-C** support. (b) FE and product distribution of electroreduction of CO_2 over **N-C** support at different applied potentials.

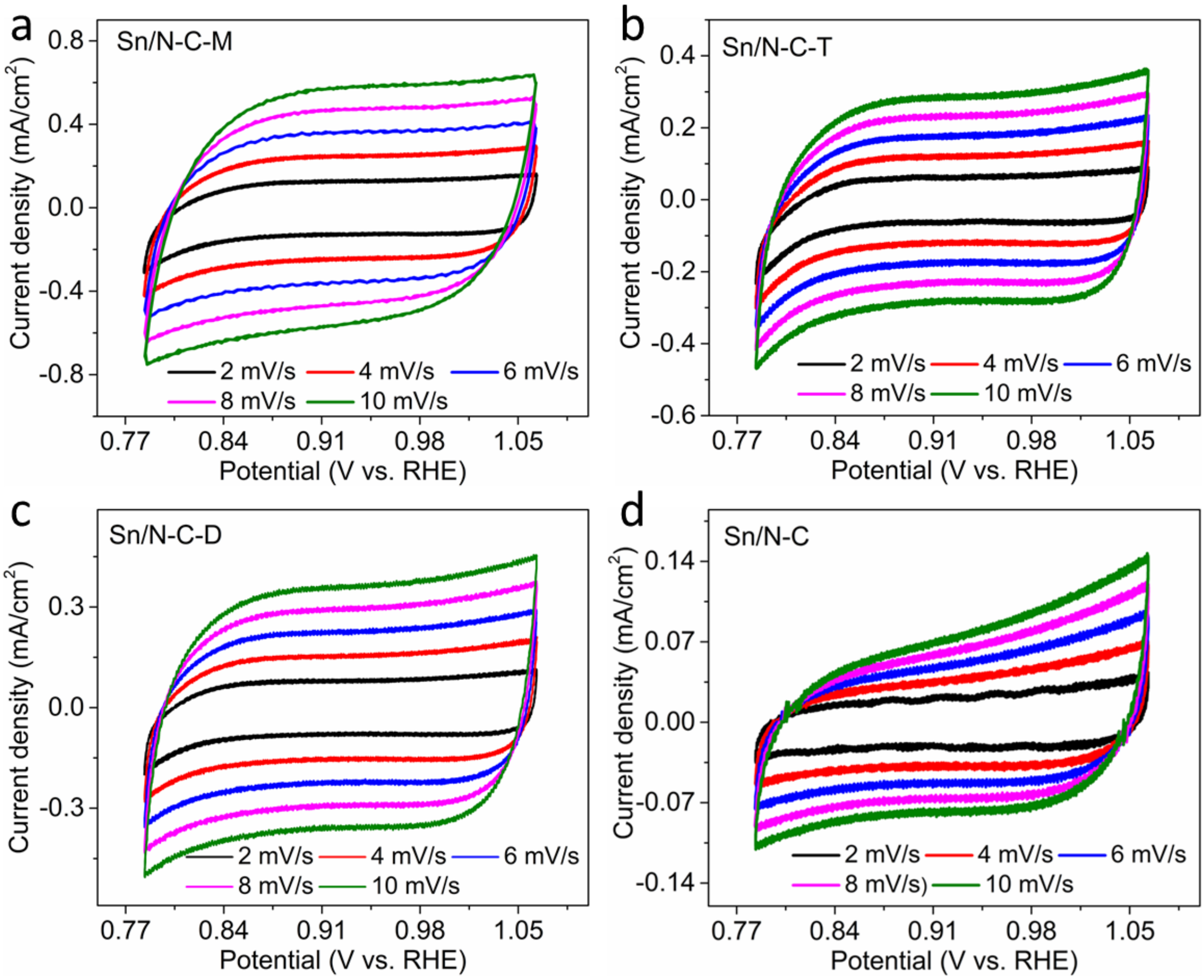

Figure S39. Electrochemical double layer capacitance (C_{DL}) measurement to estimate the electrochemical active surface area (ECSA) of the catalysts. Typical CV curves of the (a) **Sn/N-**

C-M, (b) **Sn/N-C-T**, (c) **Sn/N-C-D**, and (d) **Sn/N-C** in a region with minimal Faradaic current at different scan rates (2-10 mV s^{-1}).

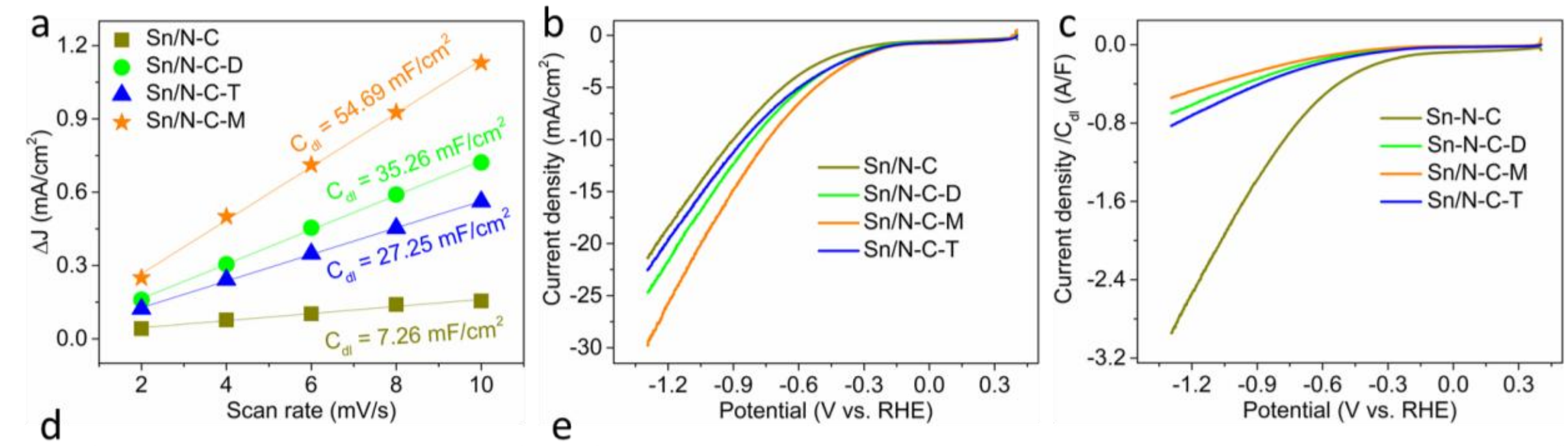

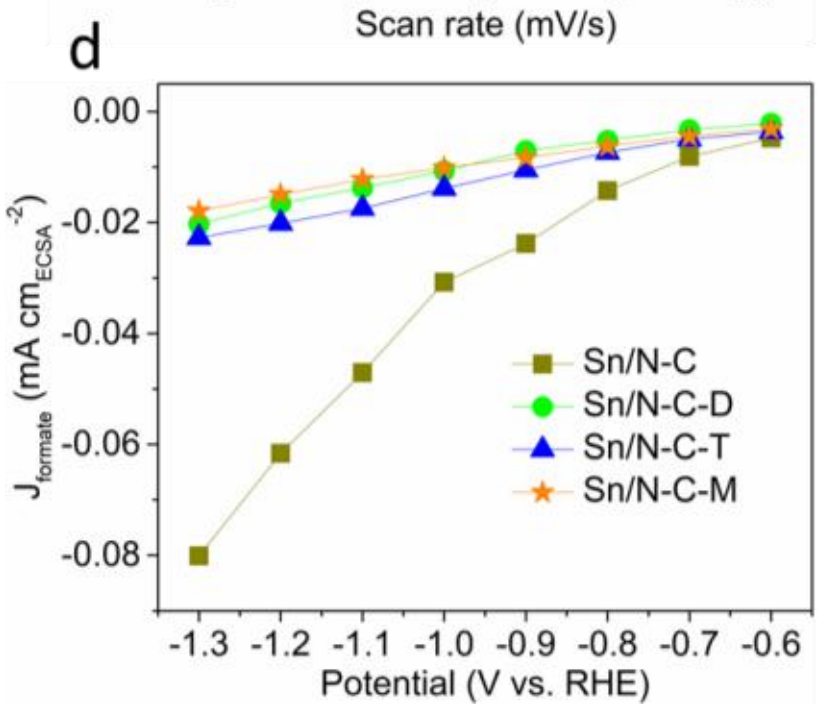

e

Catalysts	Apparent TOF (h^{-1})	Apparent TOF normalized by ECSA (h^{-1} cm^{-2})
Sn/N-C	860	4.8
Sn/N-C-T	915	1.3
Sn/N-C-D	1057	1.2
Sn/N-C-M	1450	1.1

Figure S40. (a) Capacitive current *($\Delta J = J_a - J_c$)* plotted against scan rates to estimate double layer capacitance (C_{dl}) for **Sn/N-C-M**, **Sn/N-C-T**, **Sn/N-C-D**, **Sn/N-C** catalysts at 0.924 V vs. RHE. (b, c) LSV curves over **Sn/N-C-M**, **Sn/N-C-T**, **Sn/N-C-D**, **Sn/N-C** catalysts in CO_2-saturated 0.1 M $KHCO_3$ electrolyte before and after normalization with C_{dl}, respectively. (d) ECSA-normalized partial current densities for formate production of **Sn/N-C-M**, **Sn/N-C-T**, **Sn/N-C-D**, and **Sn/N-C** catalysts. (e) Comparison of the apparent TOFs and ECSA-normalized apparent TOFs of the four catalysts at -1.3 V vs. RHE.

The electrochemically active surface area (ECSA) was calculated using the equation ECSA = $\frac{C_{DL}}{C_S}$, where C_s is the specific capacitance of a smooth surface, taken to be 40 μF cm^{-2}.[8] Since the measurements were performed on electrodes with a surface area of 1 cm^2, the measured C_{DL} values directly reflect the capacitance of the catalyst-coated electrodes ($C_{DL} = C_{DL\ total} \times S$), where S represents the surface area of the electrode, 1 cm^2. Based on this relationship, the estimated ECSA values for the **Sn/N-C-M**, **Sn/N-C-D**, **Sn/N-C-T**, and **Sn/N-C** catalysts were calculated to be 1,367 cm^2, 881 cm^2, 681 cm^2, and 181 cm^2, respectively.

Note: ECSA normalization was performed to distinguish the contribution of electrochemically accessible active-site density (approximated by ECSA) from the apparent activity of the accessible sites. As shown in figures (d) and (e), the ECSA-normalized partial current density and apparent TOF reveal the activity contribution per accessible surface area, whereas the apparent TOF reflects the combined effect of accessible site density and site activity. Although **Sn/N-C-M** exhibits a lower ECSA-normalized activity, its significantly larger ECSA provides a

substantially higher density of electrochemically accessible active sites, resulting in the highest overall catalytic performance among the investigated catalysts.

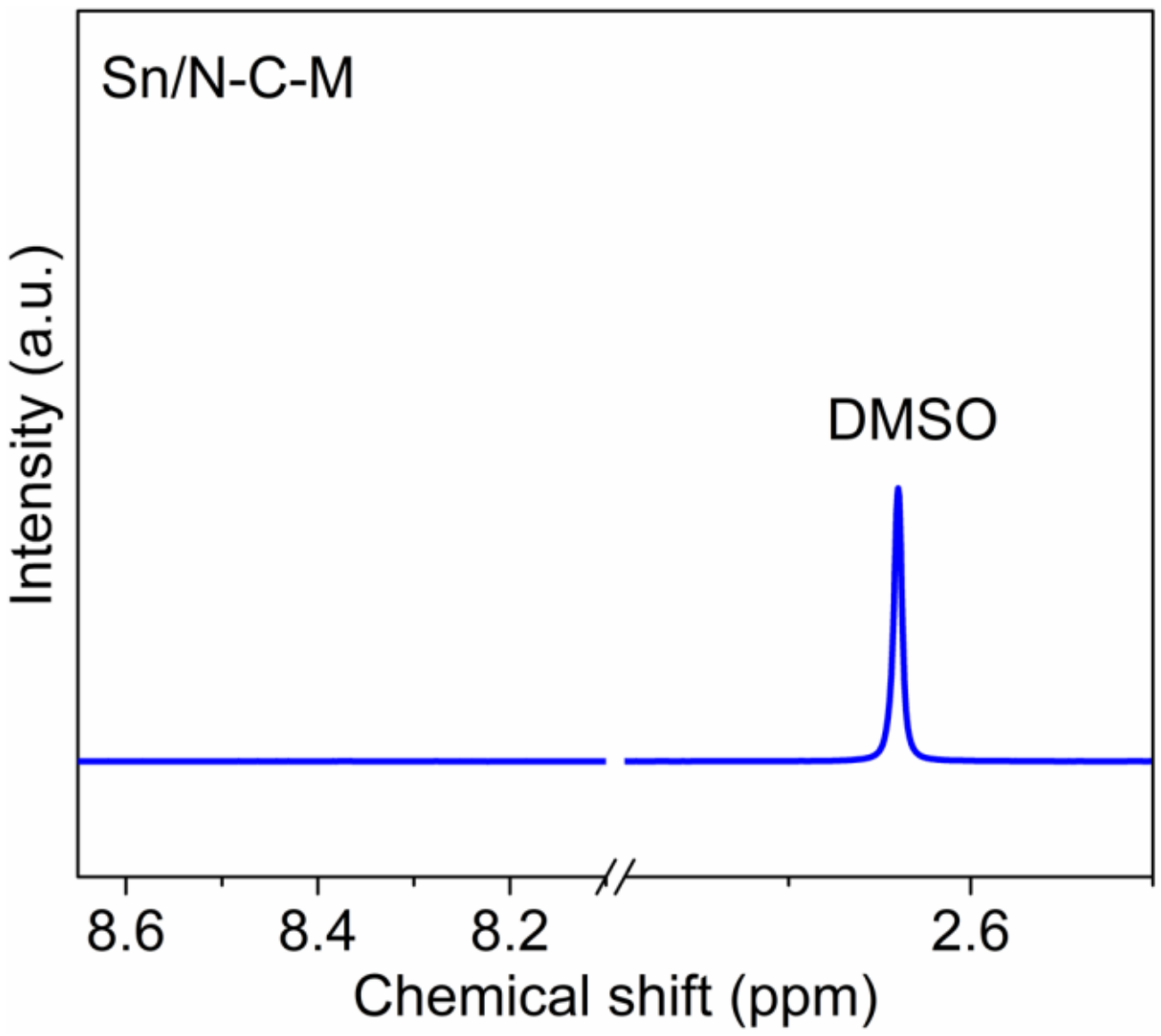

Figure S41. ^{1}H-NMR spectrum of the liquid sample collected from the cathode side after 3h of electrocatalysis in Ar-saturated 0.1 M $KHCO_3$ over **Sn/N-C-M** at -1.3 V vs. RHE.

3. Post-stability characterization

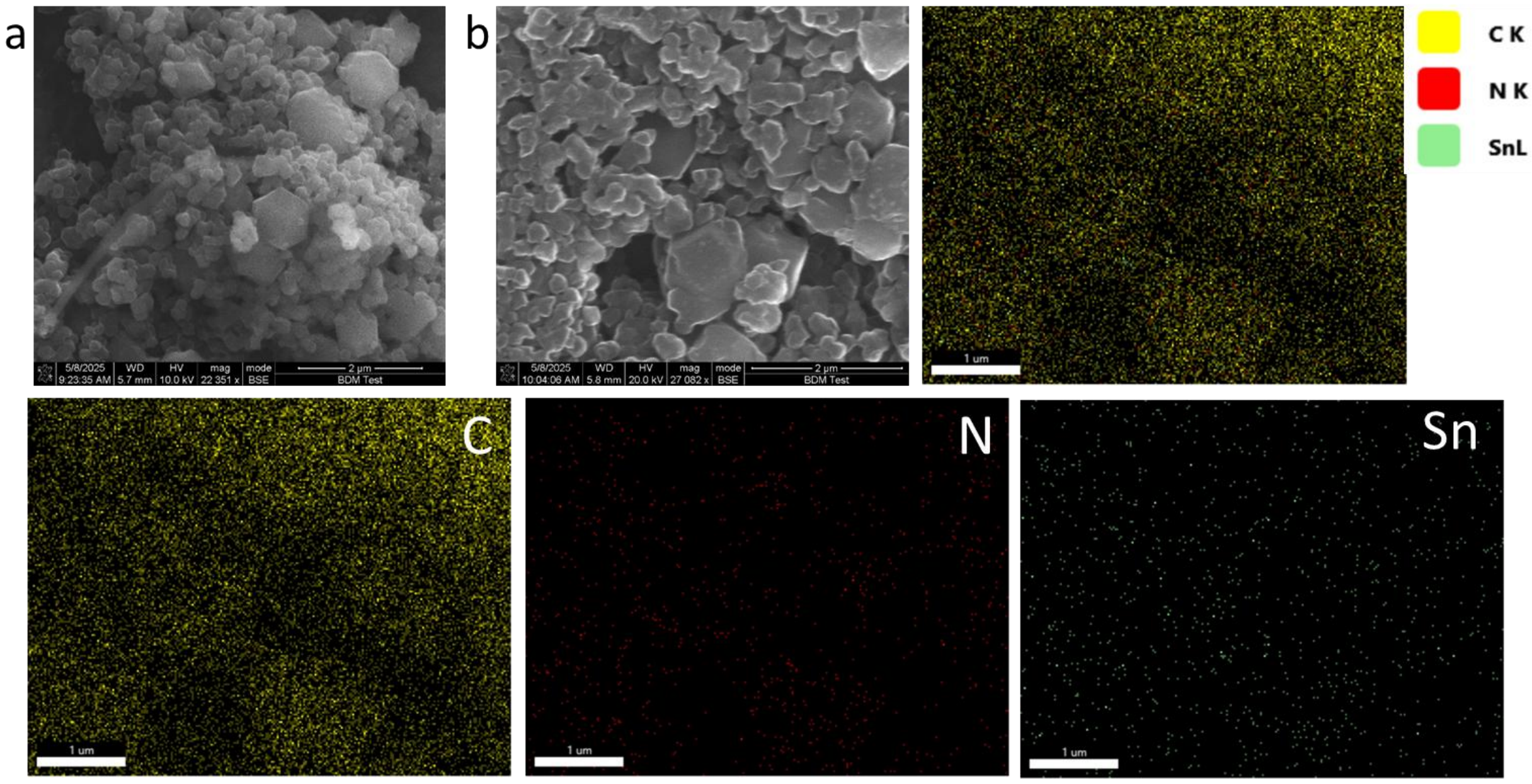

Figure S42. SEM image and the corresponding C, N, and Sn EDS elemental mappings of single-site **Sn/N-C-M** catalyst after the stability test for 27 hours.

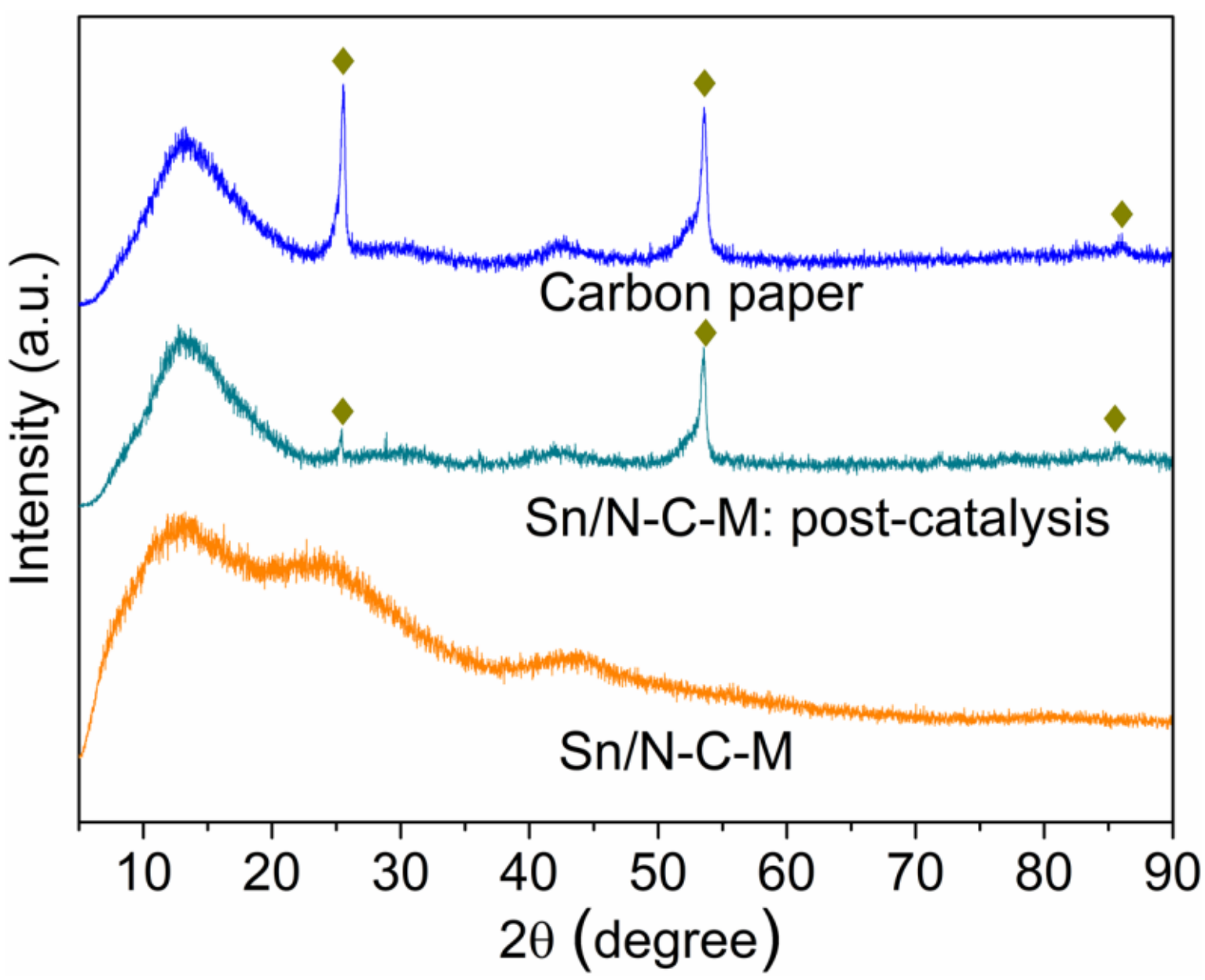

Figure S43. XRD patterns of the **Sn/N-C-M** catalyst before and after the stability test for 27 hours and the substrate carbon paper.

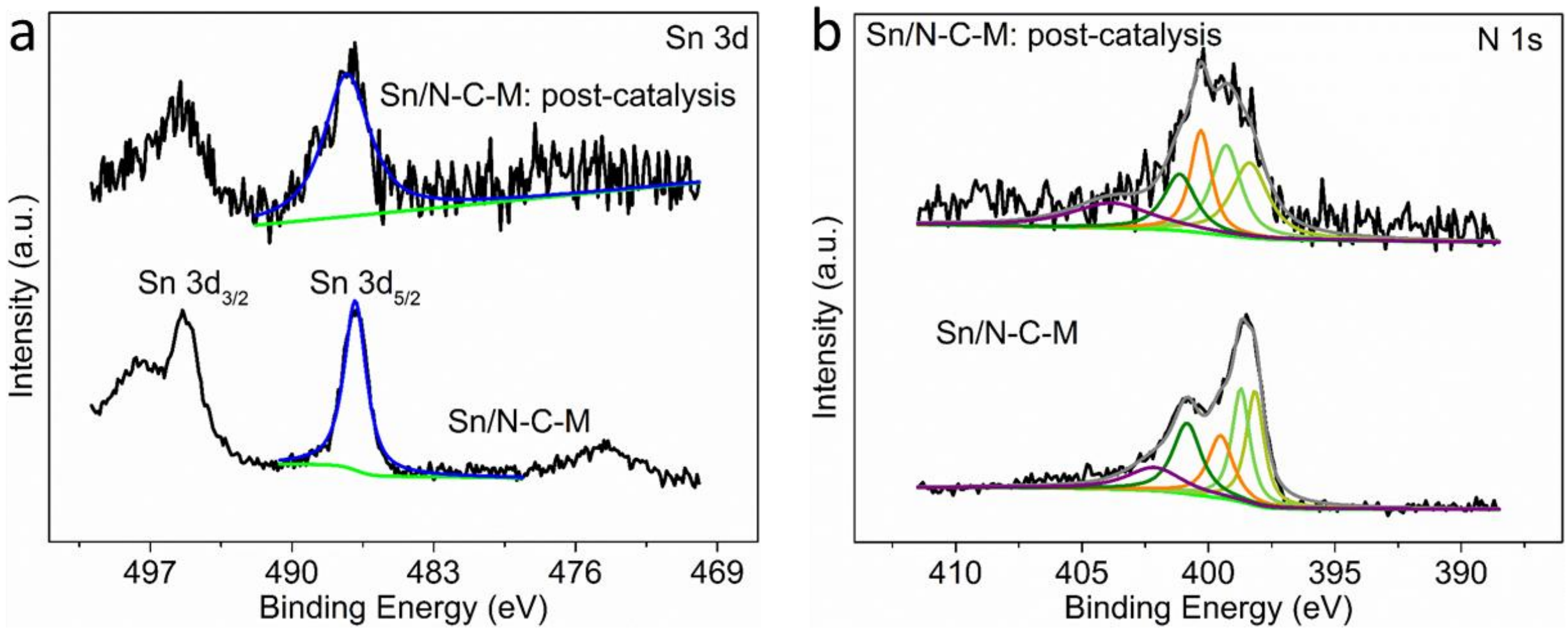

Figure S44. Deconvoluted XPS spectra: (a) Sn 3d XPS spectra (b) N 1s XPS spectra for **Sn/N-C**, **Sn/N-C-M** catalyst before and after the stability test for 27 hours.

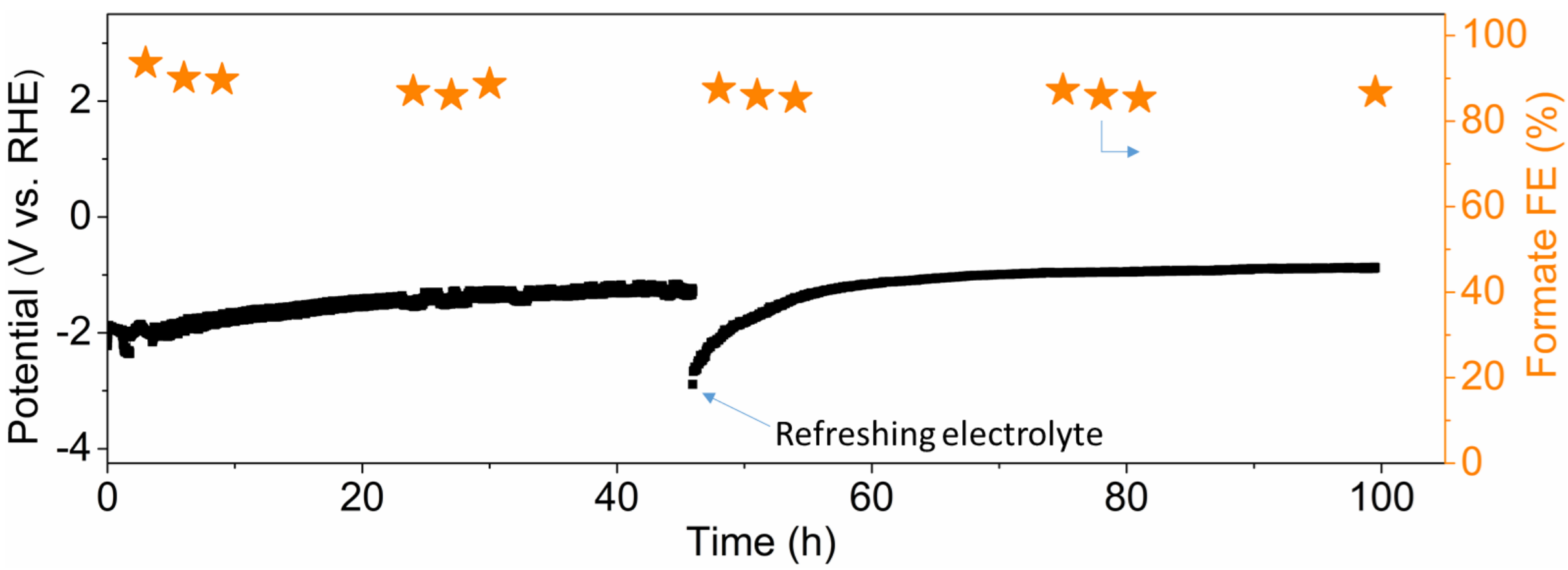

Figure S45. The stability test by chronopotentiometric measurement (black curve) and the corresponding FE of formate (orange star) using **Sn/N-C-M** catalyst at 100 mA cm^{-2} in a flow electrolytic cell.

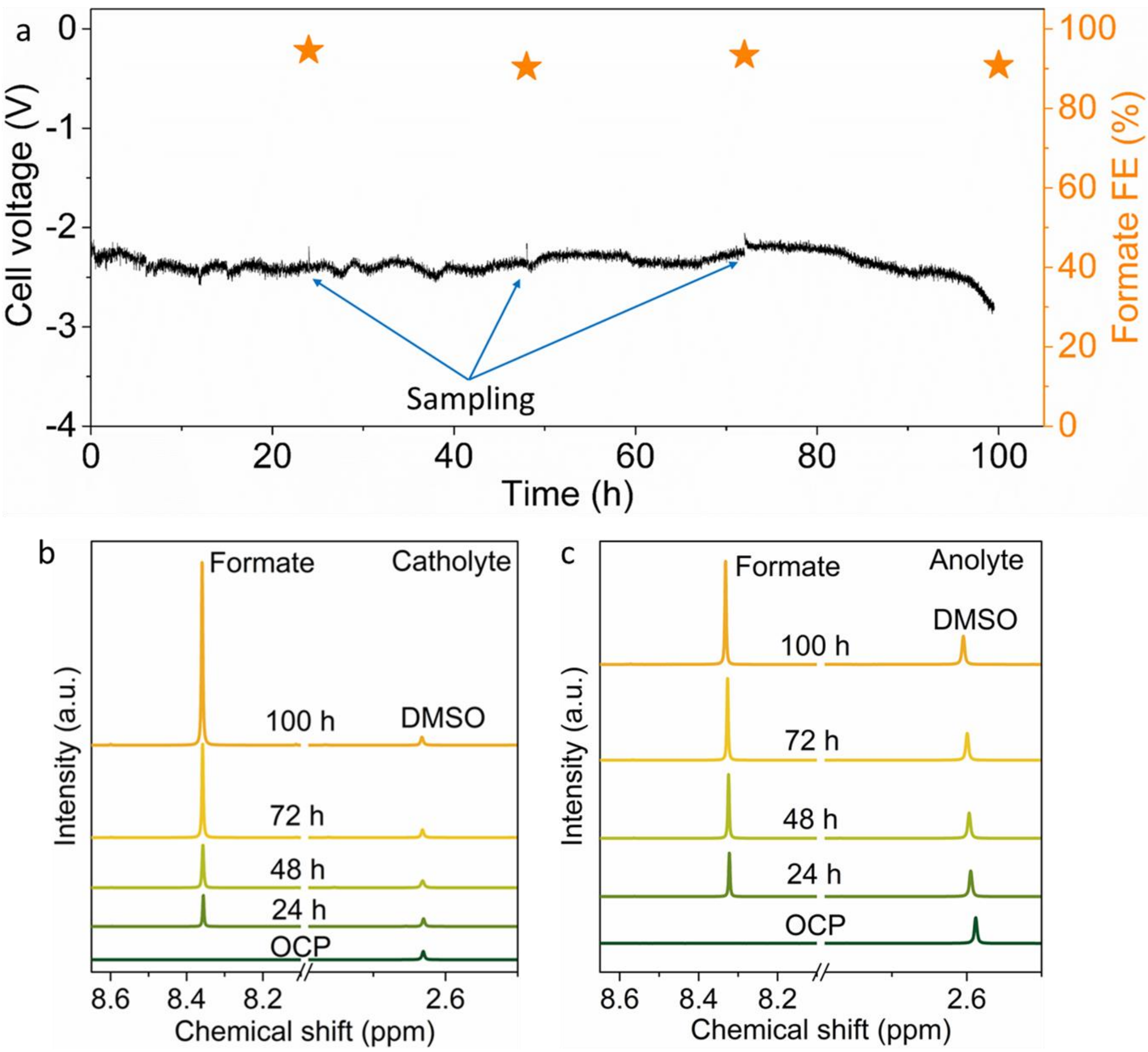

Figure S46. Long-term full-cell CO_2 electrolysis performance of the **Sn/N-C-M** catalyst in a flow electrolyzer equipped with a bipolar membrane. (a) Cell voltage and FE for formate during 100 h galvanostatic electrolysis using **Sn/N-C-M** deposited on a GDE as the cathode and commercial IrO_2 as the anodic oxygen evolution catalyst. Electrolyte samples were collected periodically during electrolysis, as indicated by the arrows. ^{1}H-NMR spectra of the catholyte (b) and anolyte (c) collected at different electrolysis times, showing the formate signal with DMSO used as the internal standard. The total FE for formate was calculated using the combined amount of formate detected in both catholyte and anolyte compartments to account for crossover through the bipolar membrane.

Table S4. Comparison of electrochemical performance metrics of **Sn/N-C-M** catalyst in half-cell and full-cell flow-cell configurations at 100 mA cm^{-2} for 100 h of CO_2 electroreduction, including formate current density, energy efficiency, and formate crossover.

Performance Metrics	Half-cell cathodic	Full-cell
Formate crossover in the anolyte (%)	-	13.7
Energy efficiency (%)	58.6	45.8
$J_{Formate}$ (mA cm^{-2})	89.69	90.8

In the half-cell configuration, a Nafion 117 membrane was employed, and no crossover products were detected. In the full-cell configuration, a bipolar membrane (BPM) was used, and formate crossover from the cathode to the anode compartment was observed and quantified. For the flow electrolyzer, the EE was evaluated during continuous electrolysis at a current density of 100 mA cm^{-2} for 100 h, using the corresponding operating potentials. The half-cell cathodic EE was calculated using an applied cathode potential of −0.88 V vs. RHE, whereas the full-cell EE was calculated using the measured operating cell voltage of 2.83 V (absolute value of the applied cell voltage).

Table S5. Summary of *in-situ* ATR-FTIR spectral assignments for CO_2 electroreduction on **Sn/N-C-M**

Wavenumber (cm^{-1})	Spectral Assignment	Proposed Intermediate / Species	Basis for Assignment	Supporting References
1436	*v*s(OCO) vibration	*OCHO intermediate	Symmetric OCO stretching	[9]
1500	*v*as(OCO) vibration	$*HCOO^-$ species	Asymmetric OCO stretching	[10]
1615	H−O−H bending mode	Adsorbed H_2O	Interfacial water vibration	[11]
1800-1900	C≡O stretching vibration	*CO intermediate	Adsorbed *CO species	[12]
3300-3600	O−H stretching band	Interfacial water / hydrogen-bonded water network	Hydrogen-bonded O–H stretching	[13]

Peak assignments are based on comparison with previously reported *in-situ* ATR-FTIR studies of Sn-based CO_2 reduction catalysts and electrochemical interfaces. ATR-FTIR peak intensities were quantified by integrating the areas of the characteristic *OCHO (1436 cm^{-1}) and $*HCOO^-$ (1500 cm^{-1}) bands after linear baseline subtraction. The baseline was defined using local anchor points adjacent to each vibrational band, and the integrated peak areas were used for comparison of signal evolution with applied potential.

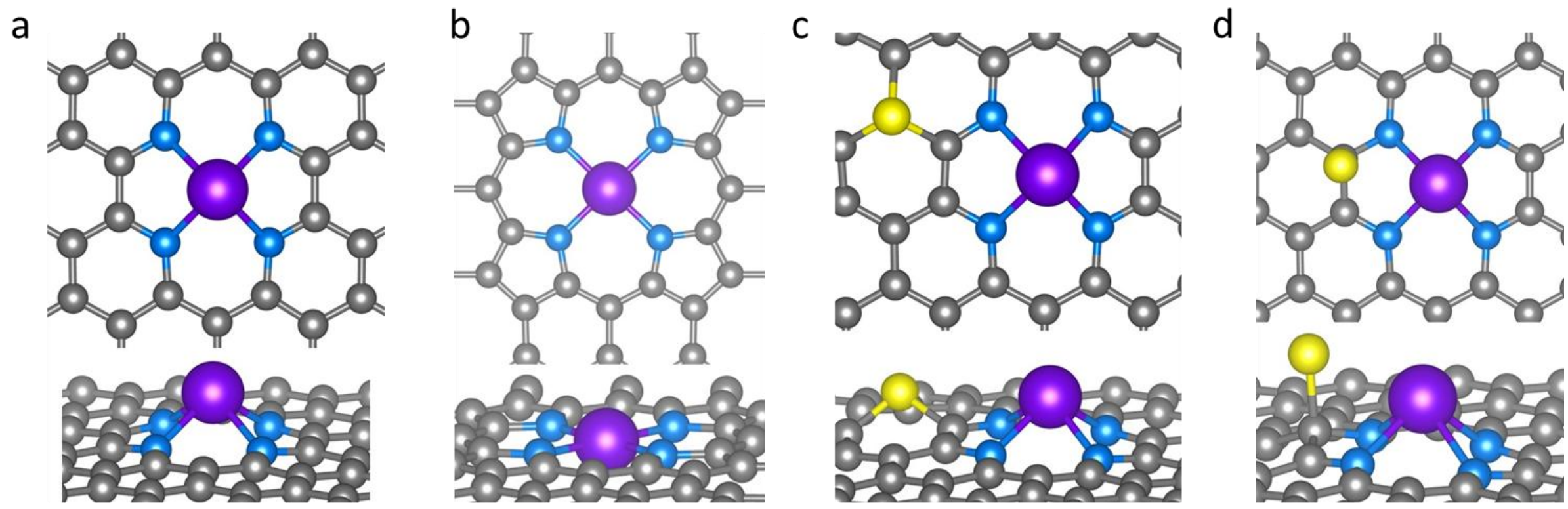

Figure S47. DFT-optimized structures of representative Sn single-site catalyst models: (a) pyridinic-N (Sn-N_4-Py), (b) pyrrolic-N (Sn-N_4-Pr), and S-modified environments, including (c) graphitic-S (Sn-N_4-gS) and (d) curved-S (Sn-N_4-cS). These models were employed to represent the range of local coordination environments relevant to the investigated catalysts and to provide qualitative insight into the experimentally observed reactivity trends.

Table S6 | Calculated Gibbs free-energy changes (ΔG) for key CO_2RR intermediates on Sn single-site models.

Systems	ΔG_1	ΔG_2	ΔG_3	ΔG_4
Sn-N_4-Py	1.102	1.114	-0.398	-0.422
Sn-N_4-Pr	-1.001	-1.301	0.954	1.182
Sn-N_4-gS	1.099	0.167	-0.367	0.518
Sn-N_4-cS	0.639	0.003	0.051	0.669
				Energy unit: eV

ΔG_1: *CO_2 → *COOH
ΔG_2: *OCO → *OCHO
ΔG_3: *COOH → *CO
ΔG_4: *OCHO → *CO

Summary of the calculated ΔG values for *CO_2 activation, *OCHO formation (leading to HCOOH), and *COOH formation (leading to CO) on Sn-N_4-Py, Sn-N_4-Pr, Sn-N_4-gS, and Sn-N_4-cS coordination environments. The results show that *OCHO formation is generally more favorable than *COOH formation across the investigated models, except for the Sn-N_4-Py model, indicating an overall thermodynamic preference for the formate pathway.

Table S7. Comparison of the electrocatalytic performance of Sn-based catalysts for the electroreduction of CO_2 to formate from recent literature operated in different aqueous electrolyte.

Samples	Formate FE (%)	J_{geo} (mA cm^{-2})	$J_{Formate}$ (mA cm^{-2})	EE (%)	TOF (h^{-1})	Potential (V vs. RHE)	Electrolyte	Reference
Cu-SnO_2	81	-500	-	60	-	-0.8	0.1 M $KHCO_3$	[1]
CuSn/CeO_{2-x}	98.1	-242.2	-216.8	50.1	4320	-1.2	0.5 M $KHCO_3$	[14]
SnPc-8OCH_3 @CNTs	91.7	-500	-404	-	222644	-1.2	1 M KOH	[9]
NiSn-APC	86.1	-43.7	-20.8	-	4752	-1.02	0.5 M $KHCO_3$	[15]
Single-atom Sn/ N-GO	74.3	-11.3	-	-	11930	-1.6	0.25 M $KHCO_3$	[16]
Sn quantum sheets /GO	89	-21.1	-	-	-	-1.8	0.1 M $NaHCO_3$	[17]
Sn-N-C	48	-43	-11.3	-	-	-1.15	0.5 M $KHCO_3$	[18]
SnS_2–x/NHCS	87.2	-35.3	-30.1	-	-	-1.2	0.5 M $KHCO_3$	[19]
Cu@SnO_2	70	-20	-	-	-	-1.45	0.1 M KHCO3	[20]
SnO_2 nanoparticles	81	-9.4	-8	-	-	-1.2	0.1 M KHCO3	[21]
S-doped atomically dispersed Sn nanoclusters	80	-32	-31	-	-	-1.0	0.5 M $KHCO_3$	[22]
SnPc/CNT-OH[b]	89.4	-	-74.8	-	-	-1.0	1 M KOH	[23]
SnS/GDL	88		-120			-1.3	1 M KOH	[24]
SnS_2	63.3		-			-1.31	0.1 M $KHCO_3$	[25]
Bi-Sn(In)O/CC	83.66		-54.45			-0.95	0.5 M $KHCO_3$	[26]
SnO_2/NC	87.6		-18.2			-1.33	0.5 M $KHCO_3$	[27]
**Sn/N-C-M**	82.5	-30	-23.7	46.5	1450	-1.3	0.1 M $KHCO_3$	**This work**
**Sn/N-C-M**: flow cell	92.5	-100	-92.5	68.4	2730	-0.88	0.1 M $KHCO_3$	**This work**
**Sn/N-C-M**: flow cell	90.2	-500	-451.0	50.2	13284	-1.33	0.1 M $KHCO_3$	**This work**

4. References

1. Jiang, Y.; Shan, J.; Wang, P.; Huang, L.; Zheng, Y.; Qiao, S.-Z., Stabilizing oxidation state of SnO_2 for highly selective CO_2 electroreduction to formate at large current densities. *ACS Catalysis* **2023,** *13*, 3101-3108.
2. Yue, C.; Yang, X.; Zhang, X.; Wang, S.; Xu, W.; Chen, R.; Wang, J.; Yin, J.; Huang, Y.; Li, X., Secondary Coordination Sphere Engineering of Single-Sn-Atom catalyst via P Doping for Efficient CO 2 Electroreduction. *Advanced Energy Materials* **2024,** *14,* 2401448.
3. Kresse, G.; Hafner, J., Ab initio molecular dynamics for liquid metals. *Physical Review B* **1993,** *47*, 558.
4. Perdew, J. P.; Burke, K.; Ernzerhof, M., Generalized gradient approximation made simple. *Physical Review Letters* **1996,** *77*, 3865.
5. Kresse, G.; Joubert, D., From ultrasoft pseudopotentials to the projector augmented-wave method. *Physical Review B* **1999,** *59*, 1758.
6. Grimme, S.; Antony, J.; Ehrlich, S.; Krieg, H., A consistent and accurate ab initio parametrization of density functional dispersion correction (DFT-D) for the 94 elements H-Pu. *The Journal of Chemical Physics* **2010,** *132,* 154104.
7. Grimme, S.; Ehrlich, S.; Goerigk, L., Effect of the damping function in dispersion corrected density functional theory. *Journal of Computational Chemistry* **2011,** *32*, 1456-1465.
8. Lu, P.; Yang, Y.; Yao, J.; Wang, M.; Dipazir, S.; Yuan, M.; Zhang, J.; Wang, X.; Xie, Z.; Zhang, G., Facile synthesis of single-nickel-atomic dispersed N-doped carbon framework for efficient electrochemical CO_2 reduction. *Applied Catalysis B: Environmental* **2019,** *241*, 113-119.
9. Chen, B.; Zou, H.; Gong, L.; Zhang, H.; Li, N.; Pan, H.; Wang, K.; Yang, T.; Liu, Y.; Duan, L., Molecular engineering of dispersed tin phthalocyanine on carbon nanotubes for selective CO_2 reduction to formate. *Applied Catalysis B: Environment and Energy* **2024,** *344*, 123650.
10. Tao, C.; Guopeng, W.; Zhaochi, F.; Gengshen, H.; Weiguang, S.; Pinliang, Y.; Can, L., In situ FT-IR study of photocatalytic decomposition of formic acid to hydrogen on Pt/TiO_2 catalyst. *Chinese Journal of Catalysis* **2008,** *29*, 105-107.
11. Chen, R.; Zhao, J.; Li, Y.; Cui, Y.; Lu, Y.-R.; Hung, S.-F.; Wang, S.; Wang, W.; Huo, G.; Zhao, Y., Operando Mossbauer spectroscopic tracking the metastable state of atomically dispersed tin in copper oxide for selective CO_2 electroreduction. *Journal of the American Chemical Society* **2023,** *145*, 20683-20691.
12. Zhang, L.; Feng, J.; Liu, S.; Tan, X.; Wu, L.; Jia, S.; Xu, L.; Ma, X.; Song, X.; Ma, J., Atomically dispersed Ni–Cu catalysts for pH-universal CO_2 electroreduction. *Advanced Materials* **2023,** *35*, 2209590.
13. Yue, C.; Yang, X.; Zhang, X.; Wang, S.; Xu, W.; Chen, R.; Wang, J.; Yin, J.; Huang, Y.; Li, X., Secondary Coordination Sphere Engineering of Single-Sn-Atom catalyst via P Doping for Efficient CO_2 Electroreduction. *Advanced Energy Materials* **2024,** *14*, 2401448.
14. Wang, H.; Wen, N.; Wang, Y.; Jiao, X.; Xia, Y.; Chen, D., Boosting electrochemical reduction of CO_2 to formate over oxygen vacancy stabilized copper–tin dual single atoms catalysts. *Advanced Functional Materials* **2023,** *33*, 2303473.
15. Xie, W.; Li, H.; Cui, G.; Li, J.; Song, Y.; Li, S.; Zhang, X.; Lee, J. Y.; Shao, M.; Wei, M., NiSn atomic pair on an integrated electrode for synergistic electrocatalytic CO_2 reduction. 7. She, X.; Wang, Y.; Xu, H.; Chi Edman Tsang, S.; Ping Lau, S., Challenges and opportunities in electrocatalytic CO_2 reduction to chemicals and fuels. *Angewandte Chemie International Edition* **2022,** *134*, e202211396. **2021,** *133*, 7458-7464.
16. Zu, X.; Li, X.; Liu, W.; Sun, Y.; Xu, J.; Yao, T.; Yan, W.; Gao, S.; Wang, C.; Wei, S., Efficient and robust carbon dioxide electroreduction enabled by atomically dispersed $Sn^{\delta+}$ sites. *Advanced Materials* **2019,** *31*, 1808135.
17. Lei, F.; Liu, W.; Sun, Y.; Xu, J.; Liu, K.; Liang, L.; Yao, T.; Pan, B.; Wei, S.; Xie, Y., Metallic tin quantum sheets confined in graphene toward high-efficiency carbon dioxide electroreduction. *Nature Communications* **2016,** *7*, 12697.

18. Hursán, D.; Timoshenko, J.; Ortega, E.; Jeon, H. S.; Rüscher, M.; Herzog, A.; Rettenmaier, C.; Chee, S. W.; Martini, A.; Koshy, D., Reversible Structural Evolution of Metal-Nitrogen-Doped Carbon Catalysts During CO2 Electroreduction: An Operando X-ray Absorption Spectroscopy Study. *Advanced Materials* **2024,** *36*, 2307809.
19. Li, Y.; Niu, W.; Chen, T.; Sun, Y.; Yu, M., Integrating vacancy engineering and energy-level adapted coupling of electrocatalyst for enhancement of carbon dioxide conversion. *Applied Catalysis B: Environmental* **2023,** *321*, 122037.
20. Yin, P. F.; Fu, J.; Yun, Q.; Chen, B.; Liu, G.; Li, L.; Huang, Z.; Ge, Y.; Zhang, H., Preparation of Amorphous SnO2-Encapsulated Multiphased Crystalline Cu Heterostructures for Highly Efficient CO2 Reduction. *Advanced Materials* **2022,** *34*, 2201114.
21. Kim, M. K.; Lee, H.; Won, J. H.; Sim, W.; Kang, S. J.; Choi, H.; Sharma, M.; Oh, H. S.; Ringe, S.; Kwon, Y., Design of less than 1 nm scale spaces on SnO2 nanoparticles for high-performance electrochemical CO2 reduction. *Advanced Functional Materials* **2022,** *32*, 2107349.
22. Wang, X.; Li, F.; Yin, W.-J.; Si, Y.; Miao, M.; Wang, X.; Fu, Y., Atomically dispersed Sn modified with trace sulfur species derived from organosulfide complex for electroreduction of CO2. *Applied Catalysis B: Environmental* **2022,** *304*, 120936.
23. Deng, Y.; Zhao, J.; Wang, S.; Chen, R.; Ding, J.; Tsai, H.-J.; Zeng, W.-J.; Hung, S.-F.; Xu, W.; Wang, J., Operando spectroscopic analysis of axial oxygen-coordinated single-Sn-atom sites for electrochemical CO2 reduction. *Journal of the American Chemical Society* **2023,** *145*, 7242-7251.
24. Zou, J.; Lee, C. Y.; Wallace, G. G., Boosting formate production from CO2 at high current densities over a wide electrochemical potential window on a SnS catalyst. *Advanced Science* **2021,** *8*, 2004521.
25. Kawabe, Y.; Ito, Y.; Hori, Y.; Kukunuri, S.; Shiokawa, F.; Nishiuchi, T.; Jeong, S.; Katagiri, K.; Xi, Z.; Li, Z., 1T/1H-SnS2 sheets for electrochemical CO2 reduction to formate. *ACS Nano* **2023,** *17*, 11318-11326.
26. Li, Z.; Li, Z.; Lu, H.; Song, B.; Zhao, X.; Zhou, H.; Wang, L.; Zou, Q.; Yang, Y.; Wei, M., Construction of Bi–Sn (In) O Integrated Electrode for Efficient CO2 Electroreduction to Formate. *ACS Applied Materials & Interfaces* **2025** 17, 38112–38121.
27. Wu, Z.; Jing, H.; Zhao, Y.; Lu, K.; Liu, B.; Yu, J.; Xia, X.; Lei, W.; Hao, Q., Grain boundary and interface interaction Co-regulation promotes SnO2 quantum dots for efficient CO2 reduction. *Chemical Engineering Journal* **2023,** *451*, 138477.
